# Parasitism and host behavior in the context of a changing environment: The Holocene record of the commercially important bivalve *Chamelea gallina*, northern Italy

**DOI:** 10.1371/journal.pone.0247790

**Published:** 2021-04-01

**Authors:** John Warren Huntley, Daniele Scarponi

**Affiliations:** 1 Department of Geological Sciences, University of Missouri, Columbia, Missouri, United States of America; 2 Dipartimento di Scienze Biologiche, Geologiche e Ambientali, University of Bologna, Bologna, Italy; University of California, UNITED STATES

## Abstract

Rapid warming and sea-level rise are predicted to be major driving forces in shaping coastal ecosystems and their services in the next century. Though forecasts of the multiple and complex effects of temperature and sea-level rise on ecological interactions suggest negative impacts on parasite diversity, the effect of long term climate change on parasite dynamics is complex and unresolved. Digenean trematodes are complex life cycle parasites that can induce characteristic traces on their bivalve hosts and hold potential to infer parasite host-dynamics through time and space. Previous work has demonstrated a consistent association between sea level rise and increasing prevalence of trematode traces, but a number of fundamental questions remain unanswered about this paleoecological proxy. Here we examine the relationships of host size, shape, and functional morphology with parasite prevalence and abundance, how parasites are distributed across hosts, and how all of these relationships vary through time, using the bivalve *Chamelea gallina* from a Holocene shallow marine succession in the Po coastal plain. Trematode prevalence increased and decreased in association with the transition from a wave-influenced estuarine system to a wave-dominated deltaic setting. Prevalence and abundance of trematode pits are associated with large host body size, reflecting ontogenetic accumulation of parasites, but temporal trends in median host size do not explain prevalence trends. Ongoing work will test the roles of temperature, salinity, and nutrient availability on trematode parasitism. Parasitized bivalves in one sample were shallower burrowers than their non-parasitized counterparts, suggesting that hosts of trematodes can be more susceptible to their predators, though the effect is ephemeral. Like in living parasite-host systems, trematode-induced malformations are strongly aggregated among hosts, wherein most host individuals harbor very few parasites while a few hosts have many. We interpret trace aggregation to support the assumption that traces are a reliable proxy for trematode parasitism in the fossil record.

## Introduction

Global mean sea surface temperatures are expected to rise a few degrees Celsius before the end of this century (RCP8.5 scenario—[1]). This rapid warming, and related sea level rise along with other anthropogenic impacts, are predicted to be major driving forces in shaping ecological interactions and morphology of coastal areas. In particular, there is a fast-growing body of ecological literature forecasting multiple and complex effects of global change on ecological interactions, especially in coastal environments ([2–7] among many). Among the many concerns deriving from global change’s impacts on wildlife and society, parasites are poorly profiled. This is also true for zoonotic parasites (i.e., pathogens known to infect humans), which are the most monitored in face of possible threats derived by global changes ([8] but see [9]). Notwithstanding the recently compiled literature that suggests potentially negative impacts on parasite diversity due to climate change, the effect of long term climate change on parasite dynamics is complex and still unresolved [10,11]. This, perhaps, is because the response of parasites to climate warming will depend on a variety of complex feedbacks between specific biological traits of both parasites and their hosts (e.g., dispersal capacity; complex life cycle), and interaction of parasite-host systems with environmental features (e.g., habitat fragmentation and vulnerability) (see [12,13]). Recent ecological-based studies on parasite dynamics warned that climate-induced perturbation on ecosystems are likely to alter ecosystem stability and in turn might create opportunities for the origin of new host-parasite interactions and/or to change the regional balance of parasite diversity in a given area (e.g., [9,14]). Hence, in coastal areas, global warming and associated sea-level rise might cause protracted instability. This could lead different parasitic taxa to become ecologically dominant and potentially change ecological dynamics in the affected area over long but still societally relevant time scales (see [15]). However, an empirical evaluation of parasite dynamics in the context of long-term (10^2^−10^3^ y) climate shifts is difficult to assess as adequate datasets spanning long time-series are commonly outside the reach of ecological studies. The fossil record, on the contrary, might offer insights on parasite dynamics over such long but still societally relevant time-spans. Our knowledge about parasite-host interactions in this context can be expanded trough the investigation of the recent fossil record of the latest Quaternary, which shows alternating periods of rapid sea-level rise and subsequent sea-level stabilization. This approach can be viewed as a bridge between ecological and stratigraphic studies. In this context, digenean trematodes might hold untapped potential to infer parasite host-dynamics through time and space [16–20].

The high preservation potential of bivalves coupled with a growth reaction triggered by their trematode parasites yields an unparalleled opportunity to quantitatively study parasite-host interactions in the fossil record without relying on the vagaries of exceptional preservation. In this work we examine the relationships of host size, shape, and interpreted functional morphology with parasite prevalence and abundance, how parasites are distributed across hosts, and how all of these relationships vary through time and changing environments, in the economically valuable bivalve *Chamelea gallina* from cored sediments of the Po coastal plain. Specifically, we address the following research questions:

1. How do trends in prevalence vary in relation to stratal stacking patterns of investigated Holocene sedimentary succession?
2. To what degree are temporal trends in trematode prevalence driven by variations in body size or other factors spurious to a strictly ecological interpretation?
3. It has been suggested that trematodes induce risky behavior in their molluscan hosts to make them more susceptible to their durophagous vertebrate predators, the trematodes’ definitive hosts. Is there a relationship between trematode prevalence/abundance and bivalve burrowing depth? If so, how does this vary through time?
4. In paleobiological studies the presence or absence of trematode-induced pits is the basis for calculating prevalence estimates within targeted assemblages. The abundance of these pits is assumed to be a proxy for the abundance of trematodes, however we know of no work that has directly compared parasite load to traces on living bivalves. Living parasites are generally heterogeneously distributed among their metazoan hosts [21,22] and such a pattern in the fossil record would support the assumption that pits are a reliable proxy for trematode parasitism. Are trematode-induced pits aggregated among fossil bivalves and how does this distribution change through time?

## Background studies

**Fig 1:**
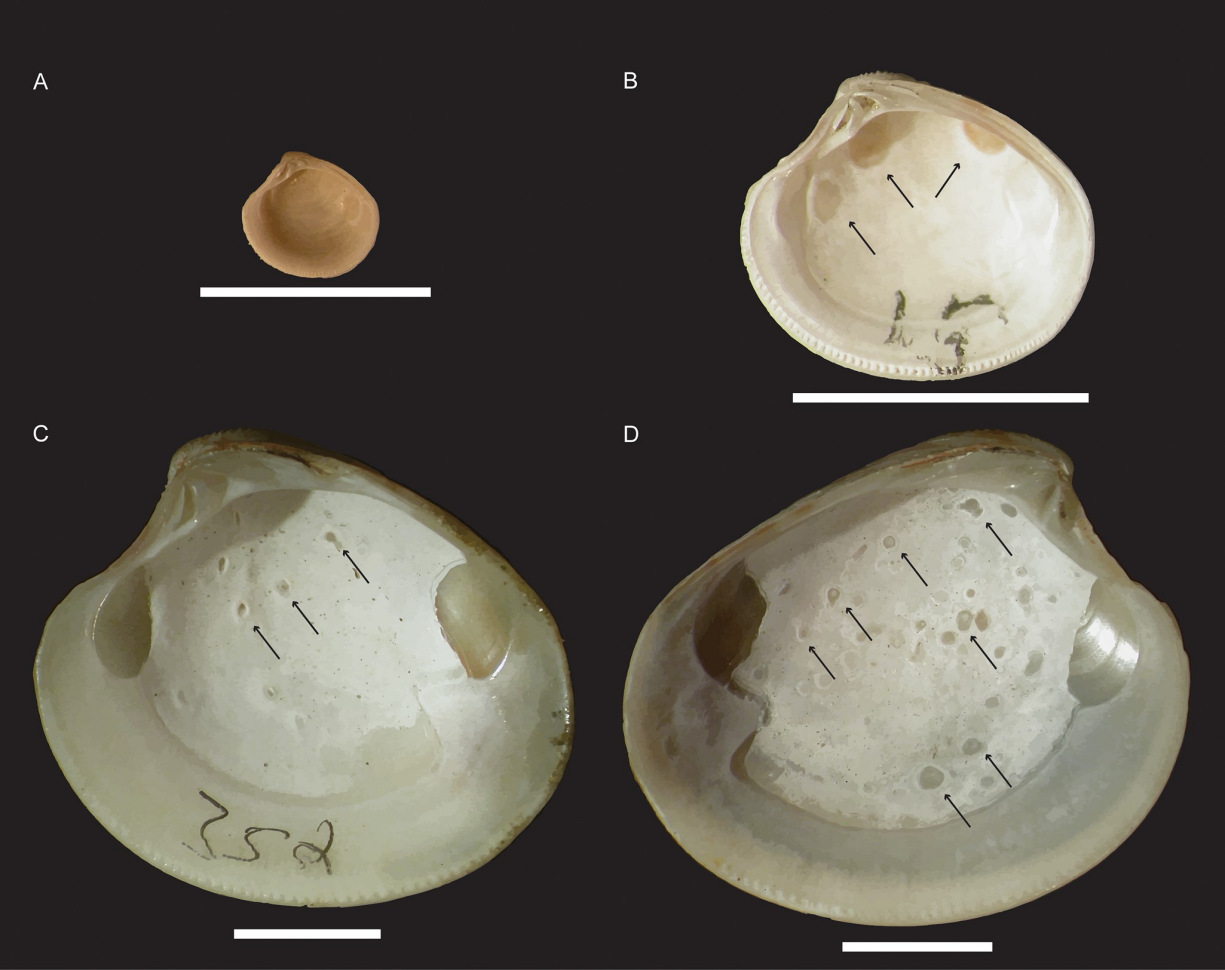
Images of *Chamelea gallina* with trematode pits. A) Specimen number 16 from core 223S5 (Locality 2) 14.9 m depth with no trematode-induced pits. B) Specimen number 15 from core 240S8 (Locality 3) 12.5 m depth with three trematode-induced pits (all indicated by arrows). C) Specimen number 352 from core 240S8 (Locality 3) 13.1 m depth with 11 trematode-induced pits (three indicted by arrows. D) Specimen number 342 from core 240S8 (Locality 3) 13.1 m depth with many trematode-induced pits (seven indicated by arrows). All scale bars equal 5 mm.

Trematodes (Platyhelminthes) are parasites with complex life cycles that have one definitive host and one or more intermediate hosts. In general, the first intermediate host, where the parasite asexually reproduces, is a mollusk. The resulting cercariae can infect a second intermediate host where encysted metacercariae are infective for the definitive host, a vertebrate that preys upon the second intermediate host. It is within the definitive host that the parasite reaches the adult stage and sexually reproduces. While infecting the second intermediate host, some trematodes can induce the growth of characteristic traces on the internal surface of their host’s valves (e.g. gymnophallids, which do not do not encyst but remain active within the bivalve). These traces are most commonly expressed as oval-shaped pits with raised rims (Fig 1), though they can vary in size and regularity of outline [20,23]. Less commonly, trematodes induce the growth of igloo-shaped structures wherein the bivalve host nearly encapsulates the trematode, but the parasite is able to maintain an opening into the interior of its host [24–26]. The oldest certain trematode-induced pits range to the Eocene London Clay [27] and the oldest igloos were recently extended from the Holocene [25] to the upper Cretaceous [26]. Liljedahl (1985) [28] identified features on silicified Silurian bivalves from Gotland that are reminiscent of igloos, but this identification is far from certain. Pits are the most commonly found trematode-induced traces from the fossil record and are the subject of this study.

Trematode-induced pits, as the only testimony of trematodes preserved in the fossil record, provide an important proxy for estimating parasite-host dynamics of these non-biomineralized organisms [29]. Preliminary analyses of trematode-host dynamics conducted at a coarse sampling resolution across the Holocene shallow marine succession of the Po coastal area suggested that trematode prevalence was significantly elevated in fossil bivalves from transgressive deposits (i.e., sediment accumulation < sea level rise) relative to those accumulated during the present high sea level stand [16]. A comparable result was obtained on the early Holocene estuarine deposits of the Pearl River Delta [17,30]. Quantitative based and integrated bio-sedimentary analyses revealed that significant peaks (i.e., above the 95% randomization-defined confidence interval) in gymnophallid prevalence occurred in the lower part of the transgressive systems tract, during the flooding of the Guangdong coastal region in correspondence to Meltwater Pulse 1c, around 9.5 to 9.2ky [31]. Along the same lines, but deeper in time, [26] reported characteristic gymnophallid-induced traces on bivalves retrieved from freshwater deposits accumulated during transgression of the Bearpaw Sea between 76.2–75.2 Ma. At higher stratigraphic resolution, comparable results were obtained within Holocene sedimentary succession of the Po Plain-Adriatic Sea system. Indeed, a recurrent association between significantly elevated prevalence values of trematode-induced traces and flooding events (i.e., surfaces or rapid deepening) was recovered along a core recording repeated alternation of floodplain and brackish deposits [32].

### Parasitism of modern *Chamelea gallina* (Linnaeus, 1758)

Modern *C*. *gallina* are infected by a diverse array of parasitic taxa. Most of the literature that we have been able to locate addresses either protozoan and trematode parasites of lagoonal mollusks [33,34] or trematodes of open marine fish [35], which is problematic as *C*. *gallina* is primarily a marine bivalve species that is also found in outer lagoon settings. Bartoli and Gibson (2007) [34] reviewed the life cycles of trematodes from the northern lagoons of the western Mediterranean and identified two taxa known to infect *C*. *gallina*. The gymnophallid *Gymnophallus rostratus* parasitizes a lucinid bivalve as its first intermediate host, up to nine species of bivalves (including *C*. *gallina*) as its second intermediate host, and the pochard diving duck *Aythia ferina* as its definitive host [34]. The echinostomid *Himasthla quissetensis* infects a nassariid gastropod as its first intermediate host, as many as 13 bivalve species (including *C*. *gallina*) as its second intermediate host, and the gull *Larus cachinnans* as its definitive host. The samples analyzed here were deposited at approximately 6–8 m (±3 m) water depth [36] and the pochard diving duck forages at depths between one and three meters [37,38] and the gull, despite its remarkable flexibility in foraging techniques [39,40], probably does not dive to such depths.

Echinostomids, including *H*. *quissetensis*, are known to encyst within bivalve mantle, gill, and foot tissue and would not likely induce malformations of the host shell [41]. Gymnophallid trematodes are exceptional in that they do not encyst as metacercariae. Rather, they remain active within their bivalve second intermediate hosts [42,43]. *G*. *rostratus* is known to occupy the central extrapallial space of *C*. *gallina* [42] and would be the more likely candidate of the two to induce a growth reaction by the bivalve. Though the specific identity of the trematode(s) that induced traces in our samples remain(s) uncertain, it was likely a gymnophallid.

## Geological setting

**Fig 2:**
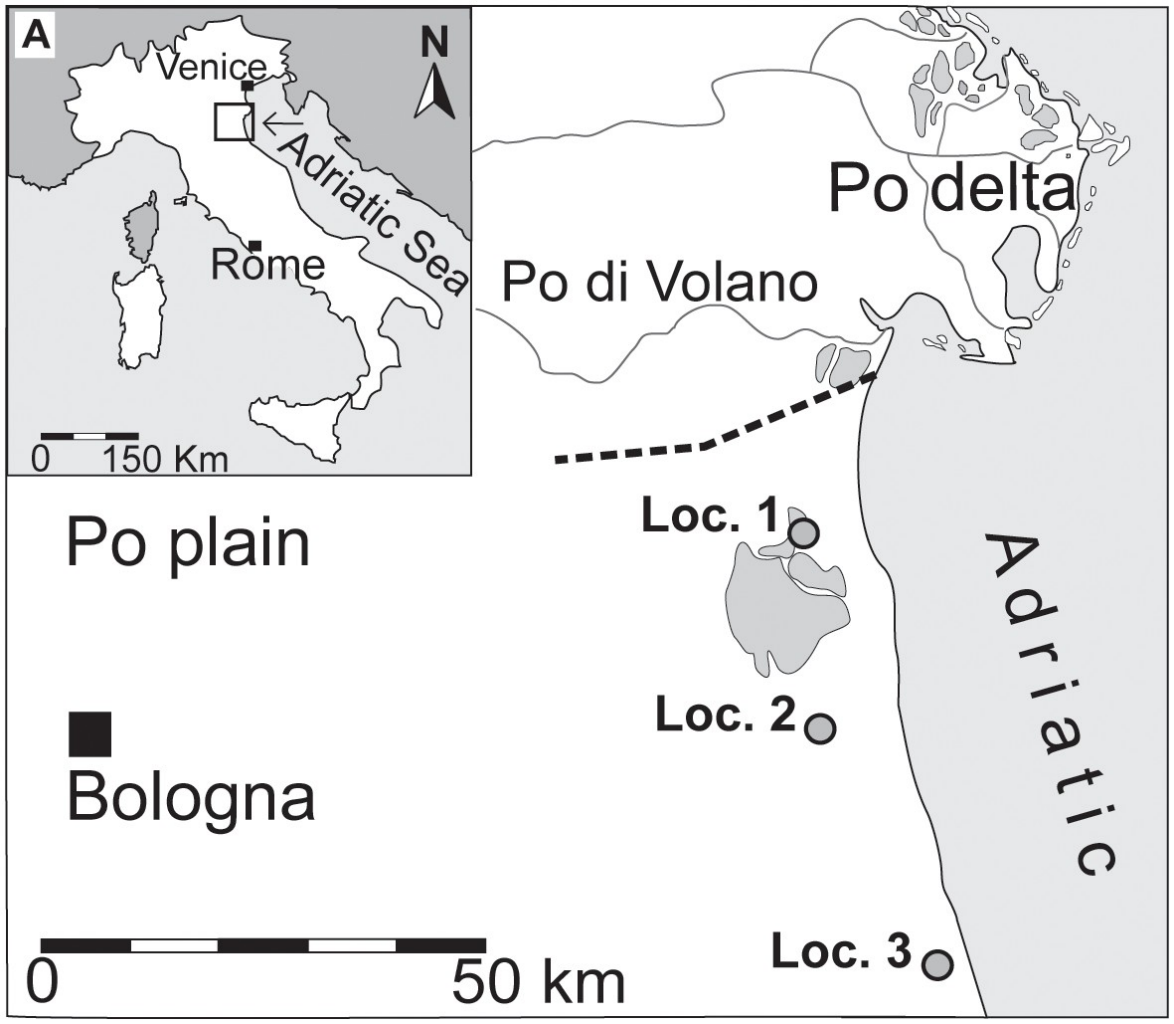
Location map of study (adapted from [16] and for illustrative purposes only).

The sedimentary succession underlying the Po coastal plain (northern Italy, Fig 2) comprises a series of 4^th^ order depositional sequences that record ~100ky glacio-eustatic fluctuations of sea-level during the latest Quaternary, when the north Adriatic shoreline, and the Po Delta, repeatedly moved back and forth for hundreds of kilometers [44,45]. This study targets the topmost and currently forming post-Last Glacial Maximum (LGM) transgressive-regressive cycle, which in the study area is <40 m thick and spans the last ~12 ky; [46]. The sharp transition from alluvial to coastal facies (in the entire study area) highlights the maximum regressive surface (MRS). The MRS, being on top of an inceptisol formed during the Younger Dryas, is a regionally prominent surface and is also easy to detect in cores [47]. The post-LGM, wedge-shaped, T-R cycle has been recently subdivided into a series of centennial to millennial-scale stratigraphic units that depict a three-stage stratal stacking pattern [46]. The lowermost deposits show a distinct, retrogradational trend interpreted as the response of depositional systems to the main pulses of early Holocene eustatic sea-level rise and reflects the landward migration of a wave-dominated estuary and adjacent coastal depositional systems (see also [47] for details). Following the stabilization of eustatic sea-level rise, there was a rapid burial of the estuarine system and the development of a wave-dominated coastal system. This latter development was characterized by longshore sediment-fed beach deposits displaying an overall aggradational to slightly progradational trend. In seaward locations, these beach deposits merged distally (a few km offshore) into a highly reworked, not easily resolvable, and environmentally condensed unit primarily developed within offshore transition settings and defined as the maximum flooding zone. This condensed unit represents several thousand years [32,48], varies in thickness along strike and is the turnaround between transgressive and normal regressive stacking patterns (i.e., TST *vs* HST). Finally, from 2.0 kyr cal BP until the last century, the coastal system experienced diffused coastal progradation, which is stratigraphically expressed by decametric thick stacking of offshore transition/prodelta, delta front/strandplain, and coastal plain facies separated by short-lived phases of muddy deposition linked mainly to autogenic coastal dynamics.

The sequence stratigraphic attribution of the investigated deposits is based on the regional stratigraphic architecture, sedimentary structures supplemented by depth profiles derived by ordinations of fossil abundance, and radiometric age calibrations [49,50]. Within core 240-S8 previously obtained bathymetric profiles encompass the onset of the late Holocene fast coastal progradation phase and ongoing work will establish radiocarbon dates for this transition. As for core 223-S5, previously obtained ^14^C ages on a fossil shell coming from the same horizons returned an age of 3.1 ky cal BP (median prob. age) and ^14^C-calibrated amino acid racemization based on 7 shells estimated median age of 2.7 kyr cal BP. Hence, our samples are within the transition from the phase of overall aggrading to quickly prograding coastal systems of the Po plain [46].

## Materials and methods

**Fig 3:**
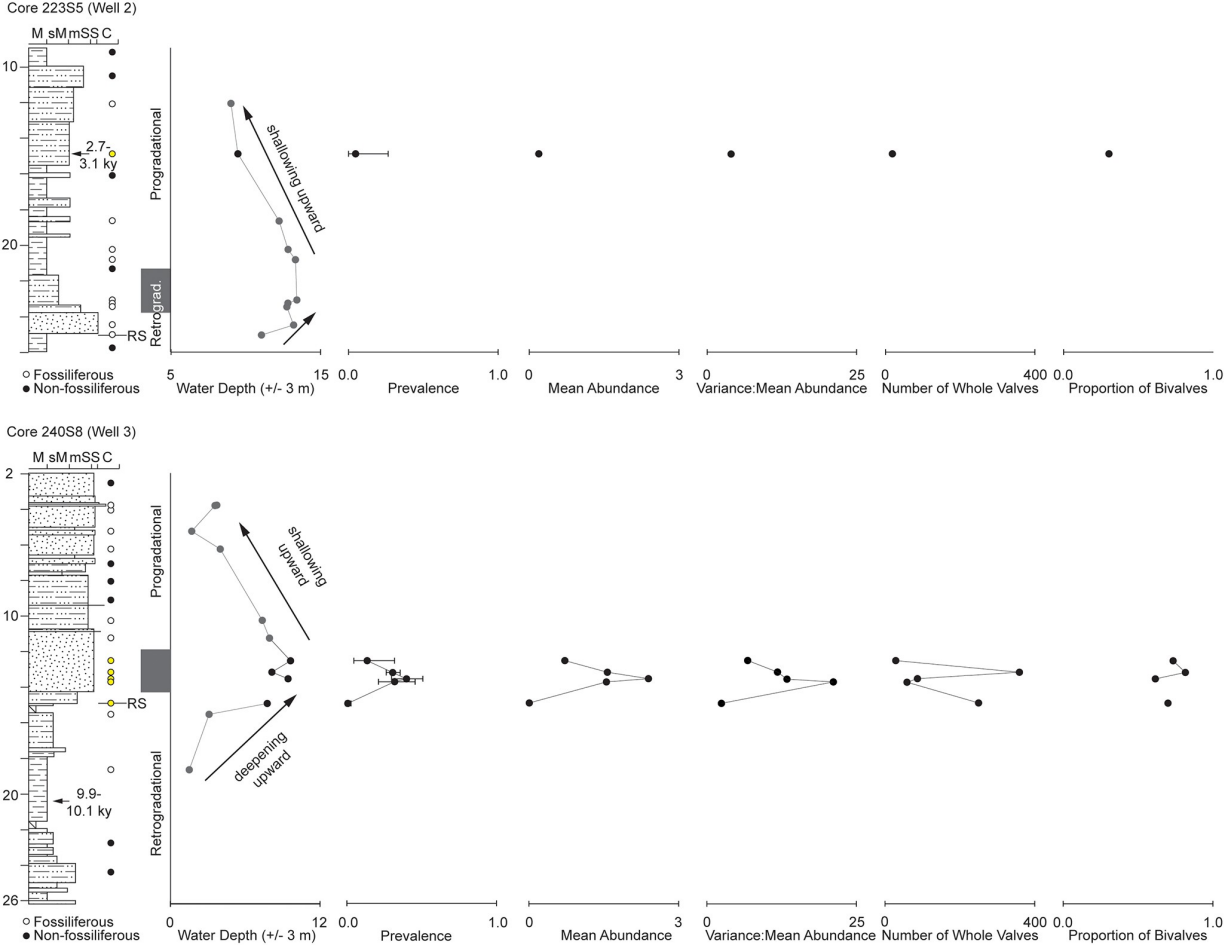
Lithostratigraphy of cores 223S5 (well 2) and 240S8 (well 3), [53]), water depth estimated by multivariate ordination of molluscan fossil counts [36] trematode prevalence and 95% confidence limits, mean trematode abundance, variance to mean abundance ratio, the number of whole valves in the sample, and the proportion of bivalves in a sample made up of *C*. *gallina*. The circles parallel to stratigraphic columns indicate samples collected from the core. Solid black circles were non-fossiliferous, hollow white circles were fossiliferous, and hollow yellow circles had *C*. *gallina* with trematode-induced pits. RS: Ravinement surface. The gray box represents the transition from retrograding outer estuary facies to aggrading/prograding deltaic facies within shoreface water depths.

We focus on the post-LGM depositional succession made of alternating continental to marine sediments, for which high-resolution stratigraphic frameworks have been established. We revisited samples utilized in establishing the stratigraphic paleobiological framework [36] and in an initial analysis of trematode-bivalve parasite-host interactions [16]. These samples were derived from three cores drilled on the southeast margin of the Po plain, 205S10 (Well 1), 223S5 (Well 2), and 240S8 (Well 3; [16,36]. Eighty-nine bulk samples (0.375 liters; Fig 3) were collected from the three cores, dried at 45°C for 24 hours, soaked in 4% H_2_O_2_, and wet-sieved through 1 mm sieves. Forty-eight of these samples contained molluscan remains and were examined for trematode-induced pits [16]. Subsequently, we collected and analyzed another sample from 13.7 m depth of 240S8. For this investigation we focused on all whole valves of *Chamelea gallina* from samples of the post LGM sedimentary succession that displayed trematode-induced pits: one sample from core 223S5 and five samples from 240S8. We counted the number of trematode-induced pits on each valve and made scaled photomicrographs using a digital camera mounted on a dissecting binocular microscope. We used *image-J* [51] to measure valve anterior-posterior length (mm), dorsal-ventral height (mm), depth of pallial sinus (mm), and surface area of valve projected onto a two-dimensional plane (mm^2^). Trematode prevalence was calculated as the proportion of valves within a sample that displayed trematode-induced pits. We calculated 95% confidence intervals of prevalence based on the binomial distribution using the *prop*.*test* function in *R* freeware (version 3.4.1 “Single Candle”; [52]). Abundance was defined as the number of pits on an individual valve and mean abundance was calculated as the total number of trematode-induced pits in a sample divided by the total number of valves in that sample (including valves that did not have trematode pits).

We conducted a Principal Components Analysis (PCA) on log_10_-transformed length, height, pallial sinus depth, and area using *prcomp* in *R* freeware (see S1 and S2 Tables and S1 Appendix for all data and *R* script). We performed Wilcoxon tests for medians to test for differences between infected and uninfected valves using the *wilcox*.*test* function and conducted Pearson and Spearman correlation analyses using the *cor*.*test* function in *R*. We used the following *R* packages to conduct analyses and produce figures: *ggplot2* [54], *factoextra* [55], *plyr* [56], *dplyr* [57], *ggthemes* [58], *viridis* [59], and *lmodel2* [60]. We assumed an alpha value of 0.05 for all analyses.

These cores were drilled for scientific purposes by the Emilia-Romagna Region, which granted us permission to sample the sub-fossil material. The specimens are housed at the Department of Biological, Geological and Environmental Sciences (Bologna, Italy), repository number MGGC 26131. This species is not protected or endangered.

## Results

We examined 801 whole valves of *Chamelea gallina* from six samples taken from two cores (Table 1). Of the total dataset, 172 valves had at least one trematode-induced pit. Sample prevalence values ranged between 0.008 and 0.402 (Table 1; Fig 3). Abundance values ranged from zero to 43. Sample mean abundance values ranged from 0.016 to 2.391 (Table 1; Fig 3). Scarponi and Kowalewski (2004) [36] provided fossil ordination-based estimates of water depth for each of the samples (with the exception of 13.7 m from 240S8; Table 1). Correlation analyses revealed no statistically significant relationships between prevalence and the number of whole *C*. *gallina* valves (R_Spearman_ = +0.143, *p* = 0.80), the proportion of bivalves that are *C*. *gallina* (R_Pearson_ = +0.348, *p* = 0.57), median PC1 values (R_Pearson_ = -0.527, *p* = 0.283), and water depth (R_Pearson_ = -0.226, *p* = 0.71). There is a significant correlation between trematode prevalence and mean abundance (R_Pearson_ = +0.990, *p* = 1.54e-04).

**Table 1:** Summary of *Chamelea gallina* samples in this study.

Sample	Core	n_whole_	n_pits_	Prevalence	Sum Pits	Mean Abundance	Water Depth	Median PC1	Environment
12.5 m	240S8	29	4	0.138	21	0.724	9.0 m	1.352	High energy, marine
13.1 m	240S8	358	111	0.310	563	1.573	7.3 m	0.246	High energy, marine
13.5 m	240S8	87	35	0.402	208	2.391	8.7 m	-0.537	High energy, marine
13.7 m	240S8	59	19	0.322	92	1.559	–	0.161	–
14.8 m	S40S8	248	2	0.008	4	0.016	6.8 m	-0.267	Low energy, marine
14.9 m	223S5	20	1	0.050	4	0.200	12.2 m	2.561	High energy, marine

n_whole_ is the number of whole *C*. *gallina* valves in the sample. n_pits_ is the number of whole valves with at least one trematode-induced pit in the sample. Water depth estimates are +/- 3 m and, along with the environment, are reported from [36].

**Fig 4:**
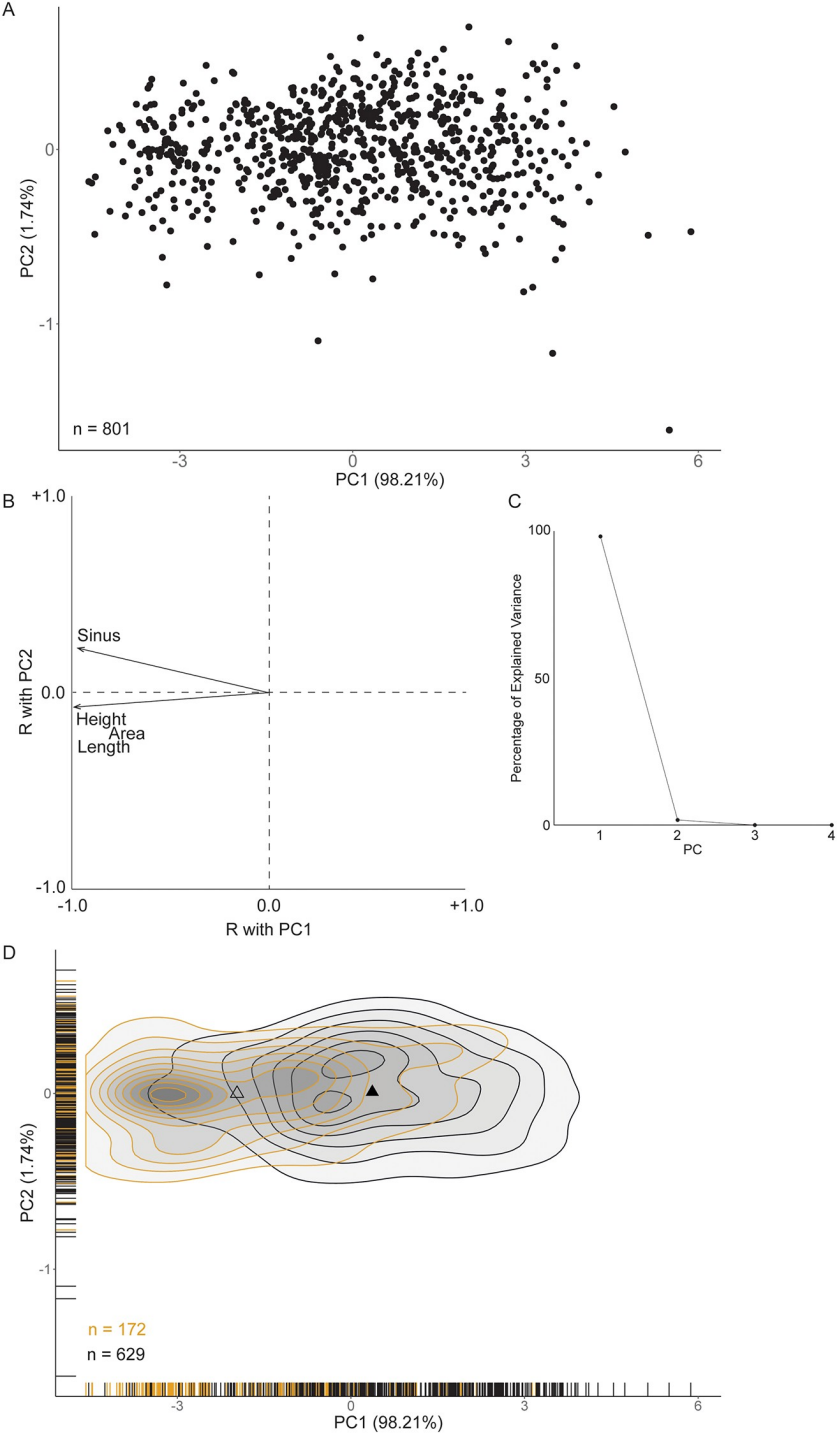
Principal Components Analysis of whole Holocene *C*. *gallina* from the Po coastal plain, Italy. A) PC1 vs PC2 (n = 801). B) Loadings plot. Values are Pearson correlation coefficients between PC1/PC2 and valve length, height, area, and pallial sinus depth. C) Scree plot relating principal components and the amount of variation explained. D) Two-dimensional density and rug plots of PC1 v PC2 by infected (yellow) and uninfected (black) valves. The triangles (hollow = infected, solid = uninfected) indicate median PC1 and PC2 values.

The first principal component (PC1) accounts for 98.21% of the variation of measurements of the 801 valves and is strongly, negatively correlated with all measured variables (Fig 4). PC2 accounts for 1.74% of the variation and is most strongly, positively correlated with pallial sinus depth (Fig 4). PC3 and PC4 account for no more than a cumulative 0.05% of the variation and are not further considered.

**Fig 5:**
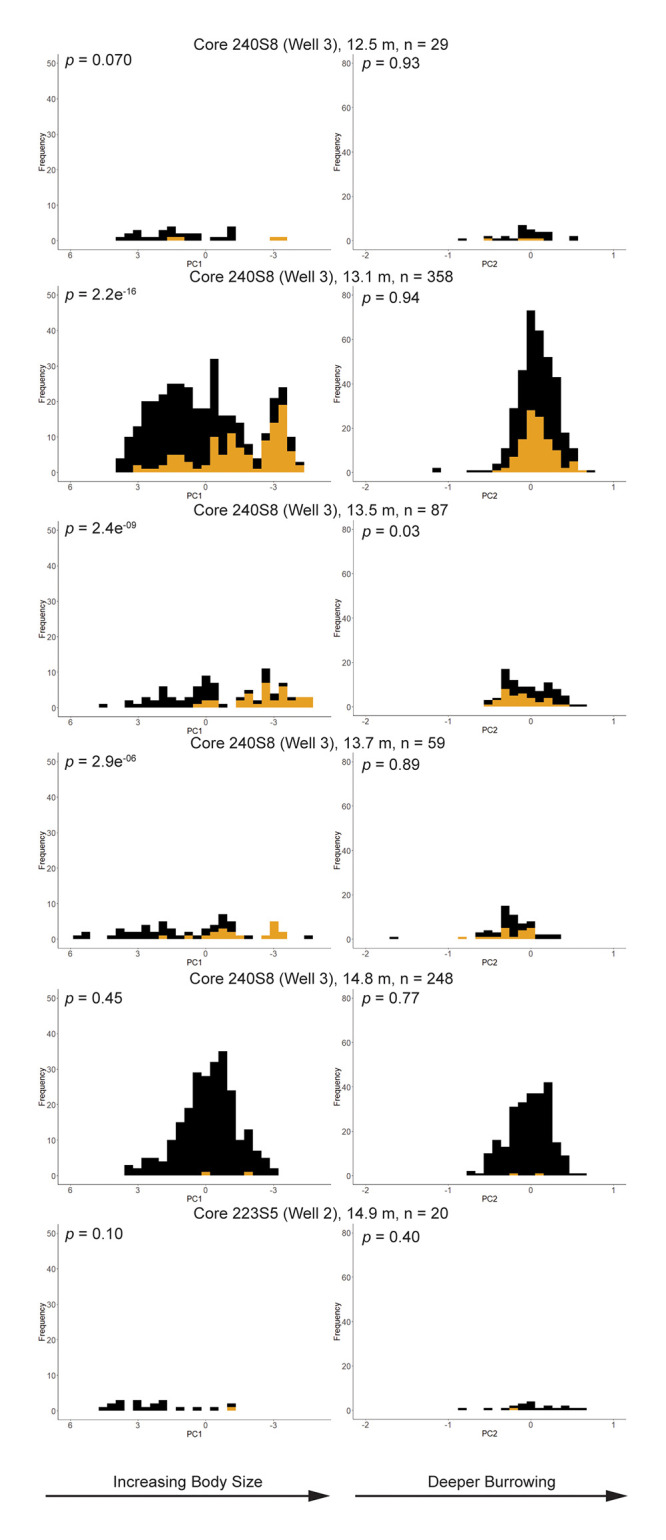
Frequency distributions of PC1 (left column) and PC2 (right column) scores by sample. The p-values are for Wilcoxon tests comparing median values of PC scores for infected (yellow) and uninfected (black) *C*. *gallina* valves.

The median PC1 score for all infected valves (-1.969) is significantly lower (*p* = 2.2e-16) than the median PC1 score for uninfected valves (+0.368). This pattern of infected valves having lower PC1 values than uninfected valves is not consistent across individual samples (Fig 5, Table 2). The samples from 13.1 m (n = 358), 13.5 m (n = 87), and 13.7 m (n = 59) display significant differences in median PC1 values between infected and uninfected valves. In contrast, median PC1 values are statistically indistinguishable between these two groups for samples 12.5 m (n = 29), 14.8 m (n = 248), 14.9 m (n = 20).

**Table 2:** Median PC1 and PC2 values for infected and uninfected valves in each sample and the *p*-values of Wilcoxon tests.

	PC1			PC2		
Sample	Infected	Uninfected	Wilcoxon	Infected	Uninfected	Wilcoxon
12.5 m	-1.053	+1.432	*p* = 0.070	-0.045	-0.038	*p* = 0.927
13.1 m	-1.741	+0.900	*p* = 2.2e-16	+0.064	+0.079	*p* = 0.936
13.5 m	-2.727	+0.381	*p* = 2.4e-09	-0.129	-0.002	*p* = 0.027
13.7 m	-1.011	+1.815	*p* = 2.9e-06	-0.200	-0.234	*p* = 0.891
14.8 m	-0.892	-0.267	*p* = 0.449	-0.068	-0.020	*p* = 0.770
14.9 m	-1.278	+2.707	*p* = 0.100	-0.257	+0.013	*p* = 0.440

There is no significant difference (*p* = 0.56) between median PC2 values of infected (-0.001) and uninfected (+0.008) valves when all samples are pooled together. This dearth of difference between groups is found in samples 12.5 m, 13.1 m, 13.7 m, 14.8 m, and 14.9 m. However, infected valves in sample 13.5 m have significantly lower PC2 values than their uninfected counterparts (Fig 5, Table 2).

**Fig 6:**
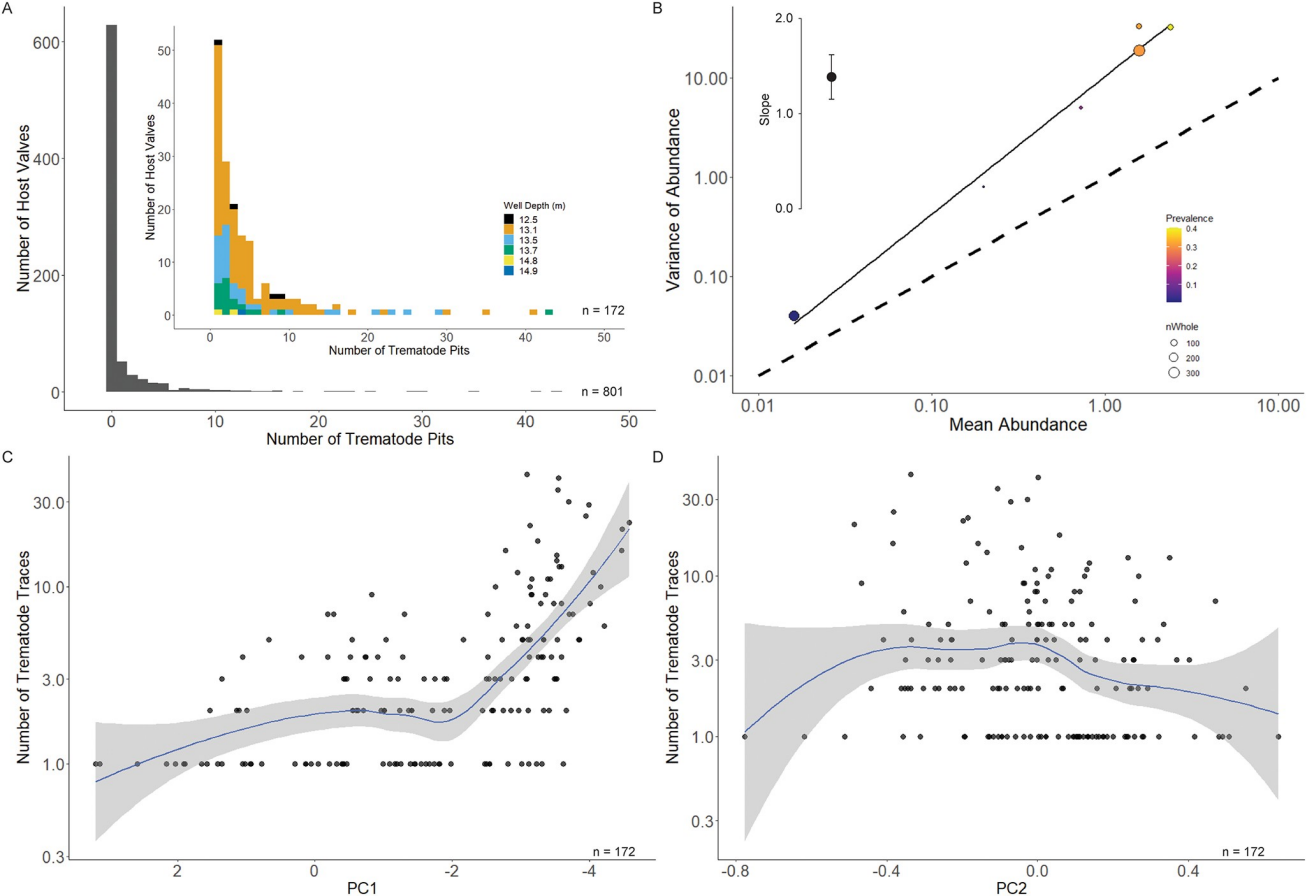
Analysis of trematode parasitism abundance. A) Frequency distribution of *C*. *gallina* valves based on the number of trematode traces per valve (abundance). Inset distribution does not include specimens without trematode pits (intensity) for clarity and is color-coded by sample. B) Sample-level analysis of mean abundance of pits and variance of the abundance of pits. Dashed line indicates the 1:1 ratio of mean: Variance. Samples on the 1:1 line would display an equal distribution of trematode pits among hosts. Values greater than a slope of one indicate aggregation of parasites. The inset figure shows the slope and 95% confidence intervals of the least square regression model. Note log_10_ scale of the x- and y-axes. C) PC1 (inverse proxy for body size, note reversed x-axis) and trematode abundance. Note the log_10_ scale of the y-axis. D) PC2 (proxy for relative pallial sinus length/relative burrowing depth) and trematode abundance. Note the log_10_ scale of the y-axis. The blue lines and gray fields in C and D are derived from the LOESS function and confidence intervals.

The frequency distribution of trematode-induced traces is strongly right-skewed, a pattern common among all samples (Fig 6). There is a significant correlation between mean abundance and variance of abundance among the six samples (R_Pearson_ = +0.929, *p* = 0.008; Fig 6). The slope of the least squares regression of log_10_-transformed mean abundance and log_10_-transformed variance of abundance is 1.39 and is significantly higher than a slope of 1.00 (Fig 6).

Trematode pit abundance is negatively correlated with PC1 values at the specimen level of observation (R_Pearson_ = -0.45, *p* = 5.40e-10). These data do not meet the assumption of homoscedasticity, so we calculated and plotted a locally estimated scatterplot smoothing (LOESS) function to better understand the relationship between PC1 and abundance (Fig 6). The negative relationship is also apparent in the LOESS function with hints of a stairstep-like pattern with lowest abundance values ~PC1 > 2, intermediate abundance values between PC1 scores of 2 and -2, and the highest abundance values (perhaps still climbing) ~PC1 < -2. There is a negative correlation between abundance and PC2 values (R_Pearson_ = -0.17, *p* = 0.027) and these data also fail to meet the assumption of homoscedasticity. The LOESS function suggests a negative relationship between the two variables, but only for valves with PC2 scores greater than zero (Fig 6).

## Discussion

### Temporal trends in prevalence and potential driving factors

In Well 3(240S8), trematode prevalence fluctuated significantly across the targeted succession, showing an overall v-shaped trajectory within the 4m thick, apparently homogenous shoreface silty to sandy unit, recording the transition from an outer estuary setting (sample 14.8 m; [53]) to the onset of a deltaic depositional system (sample 11.2 m or even as low as 13.1 m; Fig 2).

Specifically, the lowermost investigated sample is from a transgressive surface of erosion (i.e., ravinement surface) that, c. 50 km north of the study area, was dated around 8.0 kyr [48]; though, the age is likely younger here as that northern location was, unlike Well 3, located in a shallowly incised valley [47]. The associated shell lag represents an erosional and reworking phase testifying the backstepping of a barrier-lagoon complex due to the last phase of the post-LGM eustatic sea-level rise. Conversely, the uppermost sample was deposited in a shoreface setting situated at comparable water depth but belonging to a completely different geomorphologic context. Indeed, based on the regional sequence stratigraphic framework, the topmost portion of the investigated sandy unit belongs to a time-interval where the northern Adriatic coastal area already transitioned from a wave dominated estuary to a wave dominated deltaic system [46].

Huntley and Scarponi (2012) [16] noted a difference in prevalence values of pooled observations between transgressive (TST) and regressive (HST) shallow marine deposits. In sedimentary units characterized by an overall retrogradational stratal stacking pattern, prevalence values were significantly higher than in units showing a progradational pattern. Such offsets were related to the different geomorphologic configuration of the study area in response to climate-driven sea-level variations. During the early and middle Holocene, the northwestern Adriatic coastline was characterized by estuarine systems bounded seaward by a series of sandbars that isolated coastal lagoons and limited riverine influence into the Adriatic, allowing for more stable shoreface depositional settings. Indeed, in the reconstructed estuarine system, mixing between freshwater and marine waters occurred mainly in the back barrier settings, that is landward with respect to the position of the core.

Such variation in trematode prevalence within systems tracts is not unusual. Huntley et al. ([17] S1 Fig) demonstrated significantly higher trematode prevalence values among the estuarine bivalve *Potamocorbula amurensis* during the first 300 y of Holocene sea-level rise in the modern Pearl River Delta, China. Huntley and Scarponi (2015) [18] saw similar variation in prevalence in a survey of modern analog environments, with high infection values in sediment starved, oligotrophic, and overall more stable environments that characterize the modern Adriatic coastline north of the Po Delta (i.e., seaward of Venice and Grado lagoons, [18] Fig 2). Whereas, in nearshore settings around and immediately south of the Po Delta, characterized by more unpredictable environmental conditions (e.g., riverine plumes, a strong and variable sedimentary input), no or reduced trematode infection was retrieved. In addition, [19], in a higher stratigraphic resolution survey of Holocene brackish bivalves, found a strong correspondence between flooding surfaces and elevated trematode prevalence.

The consistent link between environmental modification driven by relative sea-level rise and increasing trematode prevalence (and therefore changes in trophic interactions), is documented but calls for a better understanding of the specific biotic and abiotic variables driving this pattern. Determining the nature of these factors can be difficult. Temperature and salinity are two of the most obvious abiotic environmental factors that could influence trematode-bivalve interactions. Elevated temperatures are known to increase the reproductive output and infectivity of a variety of pathogens and parasites [61,62], which could potentially explain the observed patterns. In addition, the variable temperature acts also as a complex gradient that could affect physiologic response of both the host and the parasite, but also indirectly (via sea-level rise) by influencing the geomorphologic configuration of the study area and environmental parameters (e.g., riverine plumes or higher distribution of unpredictable events). Stable isotope and trace element composition of both marine and estuarine bivalves from modern and Holocene environments could shed light on bottom-water temperature for the sampled intervals (e.g., δ^18^O and Δ_47_, Sr:Ca, Mg:Ca, Li:Ca, Li:Mg, U:Ca, and B:Ca where salinity was not stable [63–65]). The production and survival of intertidal trematode cercariae have been shown to positively correlate with salinity [66,67], which indicates that salinity could also drive prevalence among intermediate hosts. Salinity values can vary greatly in coastal settings depending upon temperature, humidity, and precipitation and overall geomorphological configuration (i.e., estuarine *vs* deltaic settings). On longer time scales, and more relevant to our samples, freshwater can be delivered greater distances into the marine environment via deltas (riverine plumes) during the late Holocene relative to longshore currents feeding shoreface settings that operated until the early part of the middle Holocene [16]. Similarly, our ongoing efforts to analyze trace element composition should give us a better understanding of freshwater input (Mn:Ca) and its influence on parasite-host interactions.

There are a number of biotic factors that could be influencing temporal trends in trematode prevalence as well. Among the most fundamental is host availability. Parasites are likely to be rare if their hosts are rare. We found no significant correlation between trematode prevalence and the number of whole *C*. *gallina* valves or the proportion of the bivalve assemblage composed of *C*. *gallina*. Though we should be cautious as we are only able to compare the results of five samples, variations in host availability do not seem to be controlling parasite prevalence.

Similarly, trends in median bivalve body size, which could be controlled by a variety of taphonomic and ecological processes, could influence temporal trends in trematode prevalence. Within samples, infected bivalves are often significantly larger than uninfected bivalves, likely due to ontogenetic accumulation of parasites [16,18,19,68]. If median body size was a driving factor of trematode prevalence trends, we would expect to see a positive correlation between the two variables. Again, with the caveat of a small sample size, we do not see such a relationship. As it stands, we can rule out the influence of host availability and temporal patterns of body size as drivers of the temporal trend in trematode prevalence.

### Size and trematode parasitism

The majority (>98%) of morphological variation among *C*. *gallina* is explained by the first principal component, which is negatively correlated with all size variables (Fig 4). We interpret PC1 as a negative proxy for body size. When considering all individuals pooled together, infected *C*. *gallina* valves are significantly larger than uninfected valves. This result is consistent with previous work on fossil [16,18,23,69] and modern bivalves [68,70–72]. Hosts tend to accumulate parasites through ontogeny; older individuals are more likely to host parasites than juveniles. The pattern becomes less clear when we compare size of infected and uninfected individuals through time by individual samples. In the sample from Well 2 (223-S5) and the lowermost sample of Well 3, traces were scanty (one and two, respectively), and testing for differences was not considered. Up-core where samples show larger sizes (Fig 5 at 13.7 m, 13.5 m, and 13.1 m core depth), infected valves were significantly larger than uninfected ones. In the samples we retrieved 59, 87, and 358 valves of which 19, 35, and 111 showed trematode traces. The youngest sample in Well 3 (12.5 m core depth) displays a substantial difference in average body size between infected and uninfected valves, but the sample size is quite small (n = 29) and the difference is not statistically significant. In sum, when prevalence is not sporadic and sample size is at least of the order of several dozen valves, infected valves tend to be larger than their uninfected counterparts, likely due to the ontogenetic accumulation of parasites.

### Shape, functional morphology, and trematode parasitism

The second principal component is strongly, positively correlated with pallial sinus depth (Fig 4). We interpret PC2 as a proxy for relative siphon length and burrowing depth [72–74].

The only significant difference in median PC2 value between infected and uninfected valves occurred in Well 3, at 13.5 m core depth (Fig 5). In this case, valves with trematode-induced pits had significantly shorter relative pallial sinus depth than those without traces of parasites. We interpret this to mean that parasitized bivalves were, on average, shallower burrowers than their non-parasitized counterparts in this sample. Why would this be the case? In order for the trematodes to complete their life cycle, the bivalve intermediate host must be preyed upon by a vertebrate predator, the trematodes’ definitive host. Shallower burrowing, in principal, causes bivalves to be at greater risk to durophagous predators.

What do we know from parasite-host interactions in modern environments? [75] examined typically infaunal, deposit feeding *Macoma balthica* individuals in a sandy intertidal flat along the Dutch North Sea coast. A number of individuals were crawling on the sediment surface at low tide, producing visible furrows. The author compared these unusual epifaunal individuals with their infaunal counterparts at two sampling locations. In both locations, 100% of epifaunal individuals were infected by trematodes while only a small proportion of infaunal individuals (5 and 13%) were infected. Swennen (1969) [75] interpreted the trematode sporocysts to be the trigger for the risky epifaunal behavior of their hosts. Contrary to [75] results, [76] examined *M*. *balthica* from tidal flats on the Russian White Sea coast and found crawlers and burrowers, all without trematode parasites. The crawling bivalves tended to be females with suppressed growth rates and elevated organic material in intestinal content relative to the burrowers, interpreted as a relocation behavior in search for more reliable food sources. Edelaar et al. (2003) [77] noted that non-parasitized *M*. *balthica* burrowed deeper in the winter to evade seasonal bird predation and reasoned that parasitized individuals, whose behavior is hypothesized to be parasite-manipulated, should remain shallowly-burrowed during the winter. However, this was not the case. In general, non-parasitized bivalves burrowed deeper than parasitized individuals but the parasitized *M*. *balthica* were not most exposed to the surface during peak avian predator activity. Edelaar et al. (2003) [77] interpreted these results as the inevitable effect of parasites draining the host’s energy stores causing the bivalves to maintain closer contact to the surface for feeding. While informative, this well-studied example may not be most comparable to our case study. The trematodes in this case infect *M*. *balthica* as a first intermediate host, castrate the bivalve, and severely reduce their energy budget.

Perhaps a more apt comparison would be [78] work on the cockle *Austrovenus stutchburyi*, a second intermediate host, whose trematode parasites encyst within its foot. The authors demonstrated that increased parasite load correlated with a diminished foot and increased predation pressure from shorebirds due to suppressed ability to burrow. Similarly, [79] demonstrated that the stout razor clam *Tagelus plebius* was more prone to avian predation when infected by trematodes. However, the clams’ ability to burrow and escape predators was not influenced by parasite intensity, which indicates a more complex mechanism driving this relationship.

A more complex scenario emerges when we consider the two aforementioned taxa infecting *C*. *gallina* today: the gymnophallid *G*. *rostratus* and the echinostomid *H*. *quissetensis*. It is possible that the traces are caused by the gymnophallids but host burrowing is limited by the echinostomids accumulating in the foot. Simply put, the trace fossil record and physiological damage are caused by two different parasite families whose abundance values are positively correlated through time. This scenario is possible as echinostomids and gymnophallids are often positively correlated among living bivalve hosts [21,80].

A cursory review of the literature illustrates the currently unsettled state of debate on whether trematode parasites directly manipulate their bivalve hosts’ behavior to make them more susceptible to predation by the definitive hosts, however, whether through manipulation or not, hosts seem to become more susceptible to their predators. Such is the case with one of our samples, but this effect is ephemeral. We cannot determine if trematode parasites were manipulating their hosts’ behavior to complete their complex life cycles from our data from the fossil record. There is sufficient evidence, however, that when host availability and parasite prevalence are both high, parasitized individuals are likely more exposed to their durophagous predators.

### Aggregation of trematode-induced pits

The frequency distribution of the number of trematode-induced pits on individual *C*. *gallina* valves is strongly right skewed (Fig 6) and this is consistent with the aggregation of parasites among bivalve hosts. Parasitologists often test for aggregation by comparing the variance: mean ratio of abundance. Here we estimate abundance as the number of trematode induced pits per individual. A variance: mean ratio equal to one indicates a random distribution of parasites across hosts and a value significantly greater than one suggests that parasites are aggregated among hosts [22,81]. When testing for aggregation among multiple samples the slope of the regression model line comparing log_10_ variance and log_10_ mean abundance can be used in the same way. This slope for our data (1.39) is significantly higher than a slope of one, which we interpret as strong evidence for aggregation of parasitic traces among *C*. *gallina*.

It is important to address the assumption we have made in all of our previous analyses of prevalence, which is that the trace fossil record of trematode-induced pits is a reliable proxy of the presence/abundance of the trematode parasites that infected living bivalves. We know of no work that has directly compared parasite load in the extrapallial space to traces on valves in living bivalves, but an examination of living bivalves often identifies far more individual trematodes than the number of pits found on (sub-)fossil valves. Therefore, it is doubtful that the abundance of pits directly corresponds to the number of parasites. The aggregation of trematode-induced pits in these samples is consistent with the aggregation of parasites found in a variety of modern hosts. For example, in their classic meta-analysis of macroparasite abundance and aggregation including platyhelminths, nematodes, acanthocephalans, and arthropods, [81] found a slope of 1.551 when comparing log_10_ variance and log_10_ mean parasite burden. Taylor et al. (1978) [82] uncovered a similar pattern among free-living taxa across very broad taxonomic, body size, and geographic distribution ranges. Though the number of trematode-induced pits in a valve is certainly not a one-to-one record for the parasite load of the host bivalve *in vivo*, we interpret the result of trace aggregation to suggest that they are likely a reasonable proxy.

Shaw and Dobson (1995) [81] stated that the end result of aggregation was to increase the effective population density experienced by parasites. Most host individuals have few parasites while a small number of hosts harbor a great many. This heterogeneity is the result of some combination of variation of host exposure to infective life stages of parasites; host susceptibility once exposed; and can be strongly influenced by factors such as sex, age, health, and behavior of the host, and seasonality or genetic variation of parasites [22,83]. It is often the case with parasite-host interactions that host morbidity and mortality are dependent upon the density of parasites. When highly aggregated among definitive hosts, this density is focused on a small proportion of potential hosts, whereas with a random distribution of parasites, the burden is shared among a larger portion of the host population and the chances of parasites encountering one another is reduced. In the former, mortality is likely spread across a small proportion of the hosts and we can see how parasites can serve as a selective evolutionary force and regulate host populations [22]. The ubiquity of parasite aggregation is certainly part of why parasites are key components of healthy ecosystems [84] that promote diversity [85] and population stability [86]. In what we think is the first analysis of this kind over such a temporal scale, trematode parasite/bivalve host interactions show a remarkable stability in parasite aggregation through what is likely a few hundred years.

Similar to prevalence, there is a positive relationship between host body size and abundance. The negative relationship between PC1 and abundance is heteroscedastic and therefore we cannot use simple Pearson linear regression. However, the LOESS function enables us to investigate the general trend of this relationship. There seems to be almost a stairstep pattern between body size and the number of trematode pits. Valves with PC1 scores of approximately 2.0 or greater only have one trematode pit. The maximum number of pits increases to around six for PC1 scores between +2.0 and -2.0. Valves with PC1 scores of -2.0 or smaller (the largest body sizes) have up to 43 trematode induced pits. This relationship is not linear and does not suggest that larger valves have more parasites, only that to have a lot of parasites, a valve must be large. The lack of correlation between abundance and PC2 reveals little relationship between burrowing depth and number of parasites across the full number of valves considered.

## Conclusions

This study quantifies trematode prevalence and dynamics retrieved in the economically valuable *C*. *gallina* during the Holocene. We conclude that:

1. The v-shaped profile in infection is associated with the transition from a backstepping estuarine system to a wave-dominated deltaic setting, all occurring within the bathymetric range of shoreface deposits. Such variation in trematode prevalence within systems tracts is not unusual and the pattern is consistent with previously identified relationships between elevated prevalence and sea-level rise.
2. Infected bivalves are significantly larger than non-infected bivalves and this likely reflects the ontogenetic accumulation of parasites.
3. Temporal changes in median host body size and abundance cannot explain temporal trends in prevalence, but ongoing studies will address the roles of temperature, salinity, and productivity.
4. In one of six samples, valves with trematode-induced pits had significantly shorter relative pallial sinus depth than those without traces of parasites, meaning that parasitized bivalves were, on average, shallower burrowers than their non-parasitized counterparts. Whether trematode parasites directly manipulate their bivalve hosts’ behavior to make them more susceptible to predation continues to be debated in the literature. Our results suggest that hosts can be more susceptible to their predators through shallower burrowing, however this effect is ephemeral.
5. The distribution of trematode-induced pits among individual *C*. *gallina* valves is strongly right skewed and this is consistent through time. The regression of mean abundance and variance of abundance on a log_10_-log_10_ plot has a slope significantly greater than one, which is consistent with the aggregation of parasites and is found in a variety of modern hosts. We interpret trace aggregation to indicate that traces are a reliable proxy for trematode parasitism in the fossil record.

## Supporting information

S1 Table Data for individual specimens of *Chamelea gallina*. (DOCX)

S2 Table Summary data by sample. (DOCX)

S1 Appendix R code used for data analysis and figures. (DOCX)
