# Supplementary material for: Parasitism and host behavior in the context of a changing environment: The Holocene record of the commercially important bivalve Chamelea gallina, northern Italy
Source: PLoS One. 2021 Apr 1;16(4):e0247790. doi: 10.1371/journal.pone.0247790 (PMC8016236; doi:10.1371/journal.pone.0247790)
Supplement: S1 Table — (DOCX) [file pone.0247790.s001.docx]

**Supporting Information: Data Tables and *R* script**

**S1 Table. Data for individual specimens of *Chamalea gallina*.** HST: highstand systems tract. TST: transgressive systems tract. Reference #: identifying number for individual valve within a sample (designated by Well Depth). Length, Height, and Sinus are all reported in mm. Area is reported in mm^2^. Infestation: 0 = trematode pits absent, 1 = trematode pits present. #Trematodes = number of trematode pits on valve. This table is referred to as “Chamalea” in the *R* script appended below.

| Locality # | Well Depth (m) | Systems Tract | Taxon | Reference # | Length | Height | Sinus | Area | Infestation | #Trematodes |
| --- | --- | --- | --- | --- | --- | --- | --- | --- | --- | --- |
| 223S5 | 14.9 | HST | Chamalea_gallina | 1 | 11.61 | 10.4 | 1.35 | 86.94 | 1 | 4 |
| 223S5 | 14.9 | HST | Chamalea_gallina | 2 | 10.93 | 9.89 | 1.48 | 78.68 | 0 | 0 |
| 223S5 | 14.9 | HST | Chamalea_gallina | 3 | 8.6 | 7.61 | 1.26 | 46.87 | 0 | 0 |
| 223S5 | 14.9 | HST | Chamalea_gallina | 4 | 7.57 | 6.58 | 1.1 | 34.91 | 0 | 0 |
| 223S5 | 14.9 | HST | Chamalea_gallina | 5 | 6.1 | 5.39 | 0.99 | 23.11 | 0 | 0 |
| 223S5 | 14.9 | HST | Chamalea_gallina | 6 | 5.41 | 4.72 | 0.76 | 18.39 | 0 | 0 |
| 223S5 | 14.9 | HST | Chamalea_gallina | 7 | 5.11 | 4.67 | 0.82 | 16.73 | 0 | 0 |
| 223S5 | 14.9 | HST | Chamalea_gallina | 8 | 5.55 | 4.78 | 0.67 | 18.95 | 0 | 0 |
| 223S5 | 14.9 | HST | Chamalea_gallina | 9 | 4.63 | 4.11 | 0.61 | 13.35 | 0 | 0 |
| 223S5 | 14.9 | HST | Chamalea_gallina | 10 | 4.94 | 4.52 | 0.54 | 15.79 | 0 | 0 |
| 223S5 | 14.9 | HST | Chamalea_gallina | 11 | 4.01 | 3.45 | 0.74 | 10.13 | 0 | 0 |
| 223S5 | 14.9 | HST | Chamalea_gallina | 12 | 4.22 | 3.59 | 0.5 | 10.82 | 0 | 0 |
| 223S5 | 14.9 | HST | Chamalea_gallina | 13 | 4.55 | 4 | 0.37 | 12.9 | 0 | 0 |
| 223S5 | 14.9 | HST | Chamalea_gallina | 14 | 3.74 | 3.26 | 0.35 | 8.93 | 0 | 0 |
| 223S5 | 14.9 | HST | Chamalea_gallina | 15 | 3.6 | 3.16 | 0.61 | 8.28 | 0 | 0 |
| 223S5 | 14.9 | HST | Chamalea_gallina | 16 | 3.02 | 2.73 | 0.52 | 5.75 | 0 | 0 |
| 223S5 | 14.9 | HST | Chamalea_gallina | 17 | 3.04 | 2.81 | 0.41 | 6.01 | 0 | 0 |
| 223S5 | 14.9 | HST | Chamalea_gallina | 18 | 3.26 | 2.97 | 0.38 | 7.03 | 0 | 0 |
| 223S5 | 14.9 | HST | Chamalea_gallina | 19 | 3.01 | 2.71 | 0.36 | 5.66 | 0 | 0 |
| 223S5 | 14.9 | HST | Chamalea_gallina | 20 | 2.67 | 2.34 | 0.34 | 4.43 | 0 | 0 |
| 240S8 | 13.5 | TST | Chamalea_gallina | 1 | 26.64 | 22.61 | 2.69 | 433.17 | 1 | 21 |
| 240S8 | 13.5 | TST | Chamalea_gallina | 2 | 22.23 | 19.45 | 2.5 | 307.72 | 0 | 0 |
| 240S8 | 13.5 | TST | Chamalea_gallina | 3 | 21.32 | 18.29 | 2.32 | 284.88 | 1 | 2 |
| 240S8 | 13.5 | TST | Chamalea_gallina | 4 | 25.28 | 21.53 | 3.1 | 399.66 | 1 | 16 |
| 240S8 | 13.5 | TST | Chamalea_gallina | 5 | 25.84 | 22.6 | 3.15 | 417.92 | 1 | 23 |
| 240S8 | 13.5 | TST | Chamalea_gallina | 6 | 16.61 | 14.88 | 2.27 | 179.12 | 0 | 0 |
| 240S8 | 13.5 | TST | Chamalea_gallina | 7 | 17.78 | 15.58 | 2.32 | 198.22 | 1 | 2 |
| 240S8 | 13.5 | TST | Chamalea_gallina | 8 | 15.89 | 13.57 | 1.73 | 156.93 | 1 | 2 |
| 240S8 | 13.5 | TST | Chamalea_gallina | 9 | 22.38 | 20.31 | 2.47 | 322.85 | 1 | 25 |
| 240S8 | 13.5 | TST | Chamalea_gallina | 10 | 19.7 | 17.26 | 2.28 | 240.49 | 1 | 2 |
| 240S8 | 13.5 | TST | Chamalea_gallina | 11 | 23.95 | 21.31 | 2.67 | 369.45 | 1 | 6 |
| 240S8 | 13.5 | TST | Chamalea_gallina | 12 | 15.43 | 13.65 | 1.86 | 148.16 | 0 | 0 |
| 240S8 | 13.5 | TST | Chamalea_gallina | 13 | 21.9 | 19.29 | 2.86 | 308.02 | 1 | 29 |
| 240S8 | 13.5 | TST | Chamalea_gallina | 14 | 19.58 | 17.07 | 2.08 | 239.35 | 1 | 4 |
| 240S8 | 13.5 | TST | Chamalea_gallina | 15 | 16.11 | 14 | 1.9 | 163.26 | 1 | 2 |
| 240S8 | 13.5 | TST | Chamalea_gallina | 16 | 20.13 | 17.47 | 2.23 | 252.46 | 1 | 4 |
| 240S8 | 13.5 | TST | Chamalea_gallina | 17 | 22.19 | 20.3 | 3.12 | 322.27 | 1 | 10 |
| 240S8 | 13.5 | TST | Chamalea_gallina | 18 | 19.69 | 16.59 | 2.6 | 233.58 | 1 | 8 |
| 240S8 | 13.5 | TST | Chamalea_gallina | 19 | 15.77 | 14.3 | 1.93 | 161.51 | 0 | 0 |
| 240S8 | 13.5 | TST | Chamalea_gallina | 20 | 15.64 | 13.39 | 2.48 | 144.81 | 0 | 0 |
| 240S8 | 13.5 | TST | Chamalea_gallina | 21 | 16.29 | 14.76 | 2.51 | 172.37 | 1 | 2 |
| 240S8 | 13.5 | TST | Chamalea_gallina | 22 | 16.22 | 13.94 | 2.51 | 159.57 | 0 | 0 |
| 240S8 | 13.5 | TST | Chamalea_gallina | 23 | 15.68 | 13.91 | 1.82 | 157.58 | 1 | 3 |
| 240S8 | 13.5 | TST | Chamalea_gallina | 24 | 7.63 | 7.09 | 1.31 | 38.53 | 0 | 0 |
| 240S8 | 13.5 | TST | Chamalea_gallina | 25 | 15.86 | 14.26 | 1.8 | 160.59 | 1 | 1 |
| 240S8 | 13.5 | TST | Chamalea_gallina | 26 | 12.16 | 10.43 | 1.37 | 90.46 | 1 | 2 |
| 240S8 | 13.5 | TST | Chamalea_gallina | 27 | 16.5 | 14.86 | 1.96 | 172.55 | 1 | 2 |
| 240S8 | 13.5 | TST | Chamalea_gallina | 28 | 8.74 | 7.81 | 1.12 | 48.18 | 1 | 4 |
| 240S8 | 13.5 | TST | Chamalea_gallina | 29 | 13.24 | 11.44 | 1.63 | 108.15 | 1 | 1 |
| 240S8 | 13.5 | TST | Chamalea_gallina | 30 | 15.69 | 13.98 | 2.43 | 157.17 | 0 | 0 |
| 240S8 | 13.5 | TST | Chamalea_gallina | 31 | 16.17 | 14.43 | 2.08 | 165.04 | 1 | 5 |
| 240S8 | 13.5 | TST | Chamalea_gallina | 32 | 15.75 | 13.75 | 2.09 | 154.09 | 1 | 3 |
| 240S8 | 13.5 | TST | Chamalea_gallina | 33 | 13.84 | 12.06 | 1.65 | 118.71 | 1 | 1 |
| 240S8 | 13.5 | TST | Chamalea_gallina | 34 | 19.8 | 17 | 2.58 | 242.21 | 1 | 15 |
| 240S8 | 13.5 | TST | Chamalea_gallina | 35 | 15.5 | 13.54 | 1.95 | 148.45 | 1 | 1 |
| 240S8 | 13.5 | TST | Chamalea_gallina | 36 | 12.63 | 11.13 | 1.97 | 100.18 | 1 | 2 |
| 240S8 | 13.5 | TST | Chamalea_gallina | 37 | 18.91 | 15.9 | 2.89 | 211.9 | 0 | 0 |
| 240S8 | 13.5 | TST | Chamalea_gallina | 38 | 18.87 | 15.64 | 3.11 | 208.56 | 1 | 3 |
| 240S8 | 13.5 | TST | Chamalea_gallina | 39 | 16.31 | 14.11 | 1.72 | 161.62 | 0 | 0 |
| 240S8 | 13.5 | TST | Chamalea_gallina | 40 | 13.5 | 11.36 | 1.44 | 109.1 | 1 | 2 |
| 240S8 | 13.5 | TST | Chamalea_gallina | 41 | 12 | 10.76 | 1.38 | 91.4 | 0 | 0 |
| 240S8 | 13.5 | TST | Chamalea_gallina | 42 | 9.67 | 8.72 | 1.13 | 60.36 | 0 | 0 |
| 240S8 | 13.5 | TST | Chamalea_gallina | 43 | 11.95 | 10.7 | 1.84 | 91.22 | 1 | 1 |
| 240S8 | 13.5 | TST | Chamalea_gallina | 44 | 12.22 | 11.09 | 1.8 | 95.96 | 0 | 0 |
| 240S8 | 13.5 | TST | Chamalea_gallina | 45 | 8.46 | 7.55 | 0.84 | 45.25 | 0 | 0 |
| 240S8 | 13.5 | TST | Chamalea_gallina | 46 | 9.78 | 8.66 | 1.19 | 59.6 | 0 | 0 |
| 240S8 | 13.5 | TST | Chamalea_gallina | 47 | 8.84 | 7.62 | 1.01 | 47.5 | 0 | 0 |
| 240S8 | 13.5 | TST | Chamalea_gallina | 48 | 8.29 | 7.29 | 1.17 | 43.13 | 1 | 1 |
| 240S8 | 13.5 | TST | Chamalea_gallina | 49 | 8.75 | 8.2 | 1.46 | 51.79 | 0 | 0 |
| 240S8 | 13.5 | TST | Chamalea_gallina | 50 | 8.88 | 8.03 | 1.44 | 50.9 | 0 | 0 |
| 240S8 | 13.5 | TST | Chamalea_gallina | 51 | 8.59 | 7.15 | 0.99 | 43.73 | 0 | 0 |
| 240S8 | 13.5 | TST | Chamalea_gallina | 52 | 6.69 | 6.22 | 0.99 | 30.05 | 0 | 0 |
| 240S8 | 13.5 | TST | Chamalea_gallina | 53 | 7.73 | 7.04 | 0.89 | 38.46 | 0 | 0 |
| 240S8 | 13.5 | TST | Chamalea_gallina | 54 | 7.7 | 6.98 | 0.93 | 37.71 | 0 | 0 |
| 240S8 | 13.5 | TST | Chamalea_gallina | 55 | 8.6 | 7.39 | 1.01 | 44.61 | 0 | 0 |
| 240S8 | 13.5 | TST | Chamalea_gallina | 56 | 9.46 | 8.25 | 1.2 | 55.41 | 0 | 0 |
| 240S8 | 13.5 | TST | Chamalea_gallina | 57 | 8.79 | 8.14 | 1.12 | 52 | 0 | 0 |
| 240S8 | 13.5 | TST | Chamalea_gallina | 58 | 7.62 | 6.81 | 1.26 | 36.72 | 0 | 0 |
| 240S8 | 13.5 | TST | Chamalea_gallina | 59 | 8.2 | 7.47 | 1.26 | 44.01 | 0 | 0 |
| 240S8 | 13.5 | TST | Chamalea_gallina | 60 | 8.4 | 7.31 | 1.29 | 43.2 | 0 | 0 |
| 240S8 | 13.5 | TST | Chamalea_gallina | 61 | 6.87 | 6.22 | 1.11 | 31.04 | 0 | 0 |
| 240S8 | 13.5 | TST | Chamalea_gallina | 62 | 5.62 | 5.02 | 0.73 | 19.53 | 0 | 0 |
| 240S8 | 13.5 | TST | Chamalea_gallina | 63 | 5.75 | 5.06 | 0.55 | 20.8 | 0 | 0 |
| 240S8 | 13.5 | TST | Chamalea_gallina | 64 | 7.35 | 6.76 | 1.14 | 35.48 | 1 | 1 |
| 240S8 | 13.5 | TST | Chamalea_gallina | 65 | 8.86 | 7.99 | 1.32 | 51.41 | 1 | 1 |
| 240S8 | 13.5 | TST | Chamalea_gallina | 66 | 8.88 | 8.1 | 1.27 | 51.77 | 1 | 1 |
| 240S8 | 13.5 | TST | Chamalea_gallina | 67 | 6.08 | 5.65 | 0.69 | 24.56 | 0 | 0 |
| 240S8 | 13.5 | TST | Chamalea_gallina | 68 | 6.89 | 5.95 | 1.19 | 29.45 | 0 | 0 |
| 240S8 | 13.5 | TST | Chamalea_gallina | 69 | 6.4 | 5.77 | 0.95 | 25.59 | 0 | 0 |
| 240S8 | 13.5 | TST | Chamalea_gallina | 70 | 6.12 | 5.39 | 1.03 | 23.21 | 0 | 0 |
| 240S8 | 13.5 | TST | Chamalea_gallina | 71 | 4.41 | 4.3 | 0.6 | 13.34 | 0 | 0 |
| 240S8 | 13.5 | TST | Chamalea_gallina | 72 | 4.46 | 4.15 | 0.49 | 13.03 | 0 | 0 |
| 240S8 | 13.5 | TST | Chamalea_gallina | 73 | 6.07 | 5.75 | 1.03 | 24.67 | 0 | 0 |
| 240S8 | 13.5 | TST | Chamalea_gallina | 74 | 5.26 | 4.67 | 0.73 | 17.05 | 0 | 0 |
| 240S8 | 13.5 | TST | Chamalea_gallina | 75 | 5.73 | 5 | 0.63 | 20.37 | 0 | 0 |
| 240S8 | 13.5 | TST | Chamalea_gallina | 76 | 5.3 | 4.7 | 0.72 | 17.94 | 0 | 0 |
| 240S8 | 13.5 | TST | Chamalea_gallina | 77 | 5.92 | 5.27 | 0.78 | 22.73 | 0 | 0 |
| 240S8 | 13.5 | TST | Chamalea_gallina | 78 | 6.31 | 5.62 | 0.83 | 25.7 | 0 | 0 |
| 240S8 | 13.5 | TST | Chamalea_gallina | 79 | 4.93 | 4.48 | 0.74 | 15.94 | 0 | 0 |
| 240S8 | 13.5 | TST | Chamalea_gallina | 80 | 4.66 | 4.15 | 0.65 | 13.69 | 0 | 0 |
| 240S8 | 13.5 | TST | Chamalea_gallina | 81 | 4.32 | 3.76 | 0.65 | 11.99 | 0 | 0 |
| 240S8 | 13.5 | TST | Chamalea_gallina | 82 | 4.28 | 3.95 | 0.64 | 11.88 | 0 | 0 |
| 240S8 | 13.5 | TST | Chamalea_gallina | 83 | 3.66 | 3.37 | 0.41 | 8.74 | 0 | 0 |
| 240S8 | 13.5 | TST | Chamalea_gallina | 84 | 5.33 | 4.82 | 0.6 | 18.06 | 0 | 0 |
| 240S8 | 13.5 | TST | Chamalea_gallina | 85 | 3.2 | 2.98 | 0.6 | 6.81 | 0 | 0 |
| 240S8 | 13.5 | TST | Chamalea_gallina | 86 | 4.28 | 3.7 | 0.46 | 11.56 | 0 | 0 |
| 240S8 | 13.5 | TST | Chamalea_gallina | 87 | 2.66 | 2.42 | 0.4 | 4.48 | 0 | 0 |
| 240S8 | 14.8 | TST | Chamalea_gallina | 1 | 5.94 | 5.29 | 0.64 | 22.13 | 0 | 0 |
| 240S8 | 14.8 | TST | Chamalea_gallina | 2 | 6.27 | 5.63 | 0.72 | 24.8 | 0 | 0 |
| 240S8 | 14.8 | TST | Chamalea_gallina | 3 | 5.56 | 4.94 | 0.71 | 19.47 | 0 | 0 |
| 240S8 | 14.8 | TST | Chamalea_gallina | 4 | 5.54 | 4.96 | 0.77 | 19.22 | 0 | 0 |
| 240S8 | 14.8 | TST | Chamalea_gallina | 5 | 4.61 | 4.05 | 0.52 | 13.4 | 0 | 0 |
| 240S8 | 14.8 | TST | Chamalea_gallina | 6 | 4.96 | 4.46 | 0.83 | 15.54 | 0 | 0 |
| 240S8 | 14.8 | TST | Chamalea_gallina | 7 | 5.48 | 4.9 | 0.88 | 19.5 | 0 | 0 |
| 240S8 | 14.8 | TST | Chamalea_gallina | 8 | 6.21 | 5.25 | 0.6 | 22.74 | 0 | 0 |
| 240S8 | 14.8 | TST | Chamalea_gallina | 9 | 5.69 | 4.91 | 0.66 | 19.52 | 0 | 0 |
| 240S8 | 14.8 | TST | Chamalea_gallina | 10 | 5.21 | 4.64 | 0.96 | 17.65 | 0 | 0 |
| 240S8 | 14.8 | TST | Chamalea_gallina | 11 | 5.98 | 5.24 | 0.8 | 22.01 | 0 | 0 |
| 240S8 | 14.8 | TST | Chamalea_gallina | 12 | 5.32 | 4.96 | 0.88 | 19.01 | 0 | 0 |
| 240S8 | 14.8 | TST | Chamalea_gallina | 13 | 5.13 | 4.61 | 0.48 | 16.71 | 0 | 0 |
| 240S8 | 14.8 | TST | Chamalea_gallina | 14 | 5.65 | 5.17 | 0.71 | 20.83 | 0 | 0 |
| 240S8 | 14.8 | TST | Chamalea_gallina | 15 | 5.09 | 4.67 | 0.79 | 17.11 | 0 | 0 |
| 240S8 | 14.8 | TST | Chamalea_gallina | 16 | 5.36 | 4.63 | 0.57 | 17.96 | 0 | 0 |
| 240S8 | 14.8 | TST | Chamalea_gallina | 17 | 4.34 | 3.77 | 0.63 | 11.81 | 0 | 0 |
| 240S8 | 14.8 | TST | Chamalea_gallina | 18 | 4.3 | 3.8 | 0.62 | 11.55 | 0 | 0 |
| 240S8 | 14.8 | TST | Chamalea_gallina | 19 | 4.95 | 4.26 | 0.69 | 15.63 | 0 | 0 |
| 240S8 | 14.8 | TST | Chamalea_gallina | 20 | 3.79 | 3.27 | 0.49 | 9.42 | 0 | 0 |
| 240S8 | 14.8 | TST | Chamalea_gallina | 21 | 4.86 | 4.19 | 0.75 | 14.24 | 0 | 0 |
| 240S8 | 14.8 | TST | Chamalea_gallina | 22 | 4.86 | 4.4 | 0.47 | 15.16 | 0 | 0 |
| 240S8 | 14.8 | TST | Chamalea_gallina | 23 | 4.65 | 4.12 | 0.72 | 14.17 | 0 | 0 |
| 240S8 | 14.8 | TST | Chamalea_gallina | 24 | 4.4 | 3.84 | 0.59 | 12.12 | 0 | 0 |
| 240S8 | 14.8 | TST | Chamalea_gallina | 25 | 4.44 | 3.89 | 0.43 | 12.43 | 0 | 0 |
| 240S8 | 14.8 | TST | Chamalea_gallina | 26 | 3.45 | 3.1 | 0.57 | 7.67 | 0 | 0 |
| 240S8 | 14.8 | TST | Chamalea_gallina | 27 | 3.73 | 3.41 | 0.55 | 9.33 | 0 | 0 |
| 240S8 | 14.8 | TST | Chamalea_gallina | 28 | 3.21 | 3.02 | 0.56 | 7.03 | 0 | 0 |
| 240S8 | 14.8 | TST | Chamalea_gallina | 29 | 4.73 | 4.2 | 0.58 | 14.34 | 0 | 0 |
| 240S8 | 14.8 | TST | Chamalea_gallina | 30 | 9.56 | 8.54 | 1.45 | 58.7 | 0 | 0 |
| 240S8 | 14.8 | TST | Chamalea_gallina | 31 | 8.65 | 7.63 | 1.26 | 47.54 | 0 | 0 |
| 240S8 | 14.8 | TST | Chamalea_gallina | 32 | 18.16 | 15.46 | 2.38 | 201.3 | 0 | 0 |
| 240S8 | 14.8 | TST | Chamalea_gallina | 33 | 15.99 | 14.48 | 2.06 | 164.19 | 0 | 0 |
| 240S8 | 14.8 | TST | Chamalea_gallina | 34 | 16.28 | 14.3 | 2.06 | 171.35 | 0 | 0 |
| 240S8 | 14.8 | TST | Chamalea_gallina | 35 | 15.27 | 13.01 | 2.23 | 143.22 | 0 | 0 |
| 240S8 | 14.8 | TST | Chamalea_gallina | 36 | 16.22 | 14.71 | 2 | 167.34 | 0 | 0 |
| 240S8 | 14.8 | TST | Chamalea_gallina | 37 | 14.62 | 13.03 | 1.46 | 138.39 | 0 | 0 |
| 240S8 | 14.8 | TST | Chamalea_gallina | 38 | 14.54 | 12.96 | 1.94 | 135.09 | 0 | 0 |
| 240S8 | 14.8 | TST | Chamalea_gallina | 39 | 15.1 | 13.03 | 1.87 | 139.28 | 0 | 0 |
| 240S8 | 14.8 | TST | Chamalea_gallina | 40 | 16.6 | 14.42 | 1.61 | 171.14 | 0 | 0 |
| 240S8 | 14.8 | TST | Chamalea_gallina | 41 | 16.75 | 15.53 | 2.24 | 188 | 0 | 0 |
| 240S8 | 14.8 | TST | Chamalea_gallina | 42 | 13.6 | 11.99 | 1.76 | 117.01 | 0 | 0 |
| 240S8 | 14.8 | TST | Chamalea_gallina | 43 | 13.14 | 11.6 | 1.39 | 109.52 | 0 | 0 |
| 240S8 | 14.8 | TST | Chamalea_gallina | 44 | 13.84 | 11.83 | 1.6 | 117.07 | 1 | 3 |
| 240S8 | 14.8 | TST | Chamalea_gallina | 45 | 13.41 | 11.65 | 1.88 | 116.06 | 0 | 0 |
| 240S8 | 14.8 | TST | Chamalea_gallina | 46 | 12 | 10.55 | 1.24 | 90.06 | 0 | 0 |
| 240S8 | 14.8 | TST | Chamalea_gallina | 47 | 12.49 | 10.96 | 1.53 | 99.33 | 0 | 0 |
| 240S8 | 14.8 | TST | Chamalea_gallina | 48 | 10.43 | 8.99 | 1.35 | 68.66 | 0 | 0 |
| 240S8 | 14.8 | TST | Chamalea_gallina | 49 | 8.34 | 7.32 | 1.08 | 44.54 | 0 | 0 |
| 240S8 | 14.8 | TST | Chamalea_gallina | 50 | 13.88 | 12.28 | 1.94 | 123.58 | 0 | 0 |
| 240S8 | 14.8 | TST | Chamalea_gallina | 51 | 8.57 | 7.53 | 1.08 | 46.12 | 0 | 0 |
| 240S8 | 14.8 | TST | Chamalea_gallina | 52 | 11.87 | 10.52 | 1.24 | 89.89 | 0 | 0 |
| 240S8 | 14.8 | TST | Chamalea_gallina | 53 | 12.86 | 11.31 | 1.74 | 103.91 | 0 | 0 |
| 240S8 | 14.8 | TST | Chamalea_gallina | 54 | 10.83 | 10.11 | 1.63 | 78.03 | 0 | 0 |
| 240S8 | 14.8 | TST | Chamalea_gallina | 55 | 7.82 | 6.76 | 0.99 | 37.76 | 0 | 0 |
| 240S8 | 14.8 | TST | Chamalea_gallina | 56 | 11.06 | 9.78 | 1.54 | 78.86 | 0 | 0 |
| 240S8 | 14.8 | TST | Chamalea_gallina | 57 | 13.12 | 11.47 | 2.03 | 107.92 | 0 | 0 |
| 240S8 | 14.8 | TST | Chamalea_gallina | 58 | 7.19 | 6.08 | 1.09 | 31.39 | 0 | 0 |
| 240S8 | 14.8 | TST | Chamalea_gallina | 59 | 12.48 | 11.09 | 1.98 | 100.4 | 0 | 0 |
| 240S8 | 14.8 | TST | Chamalea_gallina | 60 | 12.51 | 10.82 | 1.85 | 97.06 | 0 | 0 |
| 240S8 | 14.8 | TST | Chamalea_gallina | 61 | 12.52 | 11.18 | 1.89 | 99.88 | 0 | 0 |
| 240S8 | 14.8 | TST | Chamalea_gallina | 62 | 9.63 | 8.56 | 1.33 | 58.67 | 0 | 0 |
| 240S8 | 14.8 | TST | Chamalea_gallina | 63 | 9.83 | 8.56 | 1.13 | 62.1 | 0 | 0 |
| 240S8 | 14.8 | TST | Chamalea_gallina | 64 | 8.61 | 7.57 | 1.28 | 48.34 | 0 | 0 |
| 240S8 | 14.8 | TST | Chamalea_gallina | 65 | 10.4 | 9.33 | 1.18 | 70.16 | 0 | 0 |
| 240S8 | 14.8 | TST | Chamalea_gallina | 66 | 9.61 | 8.49 | 1.25 | 56.91 | 0 | 0 |
| 240S8 | 14.8 | TST | Chamalea_gallina | 67 | 8.42 | 7.59 | 1.08 | 46.79 | 0 | 0 |
| 240S8 | 14.8 | TST | Chamalea_gallina | 68 | 13.36 | 11.84 | 1.63 | 112.64 | 0 | 0 |
| 240S8 | 14.8 | TST | Chamalea_gallina | 69 | 10.41 | 9.1 | 1.25 | 68.36 | 0 | 0 |
| 240S8 | 14.8 | TST | Chamalea_gallina | 70 | 8.79 | 8.13 | 1.15 | 51.11 | 0 | 0 |
| 240S8 | 14.8 | TST | Chamalea_gallina | 71 | 11.18 | 9.95 | 1.46 | 79.9 | 0 | 0 |
| 240S8 | 14.8 | TST | Chamalea_gallina | 72 | 9.77 | 8.39 | 1.12 | 57.96 | 0 | 0 |
| 240S8 | 14.8 | TST | Chamalea_gallina | 73 | 9.75 | 8.68 | 1.35 | 60.23 | 0 | 0 |
| 240S8 | 14.8 | TST | Chamalea_gallina | 74 | 10.74 | 9.54 | 1.49 | 73.76 | 0 | 0 |
| 240S8 | 14.8 | TST | Chamalea_gallina | 75 | 12.43 | 10.79 | 1.87 | 95.08 | 0 | 0 |
| 240S8 | 14.8 | TST | Chamalea_gallina | 76 | 7.96 | 6.94 | 1.11 | 40.3 | 1 | 1 |
| 240S8 | 14.8 | TST | Chamalea_gallina | 77 | 6.33 | 5.49 | 0.93 | 25.22 | 0 | 0 |
| 240S8 | 14.8 | TST | Chamalea_gallina | 78 | 7.38 | 6.36 | 1.04 | 34.02 | 0 | 0 |
| 240S8 | 14.8 | TST | Chamalea_gallina | 79 | 6.71 | 5.99 | 0.85 | 28.66 | 0 | 0 |
| 240S8 | 14.8 | TST | Chamalea_gallina | 80 | 10.26 | 9.05 | 1.67 | 65.43 | 0 | 0 |
| 240S8 | 14.8 | TST | Chamalea_gallina | 81 | 7.02 | 6.2 | 0.95 | 30.9 | 0 | 0 |
| 240S8 | 14.8 | TST | Chamalea_gallina | 82 | 8.06 | 6.97 | 1.04 | 39.66 | 0 | 0 |
| 240S8 | 14.8 | TST | Chamalea_gallina | 83 | 6.7 | 6.17 | 0.89 | 29.34 | 0 | 0 |
| 240S8 | 14.8 | TST | Chamalea_gallina | 84 | 7.4 | 6.63 | 0.84 | 34.6 | 0 | 0 |
| 240S8 | 14.8 | TST | Chamalea_gallina | 85 | 8.96 | 7.91 | 1.32 | 50.15 | 0 | 0 |
| 240S8 | 14.8 | TST | Chamalea_gallina | 86 | 10.51 | 9.24 | 1.45 | 69.61 | 0 | 0 |
| 240S8 | 14.8 | TST | Chamalea_gallina | 87 | 10.03 | 9.08 | 1.64 | 66.55 | 0 | 0 |
| 240S8 | 14.8 | TST | Chamalea_gallina | 88 | 10.45 | 8.89 | 1.07 | 66.33 | 0 | 0 |
| 240S8 | 14.8 | TST | Chamalea_gallina | 89 | 7.5 | 6.78 | 1.14 | 36.2 | 0 | 0 |
| 240S8 | 14.8 | TST | Chamalea_gallina | 90 | 11.21 | 10.06 | 1.15 | 81.88 | 0 | 0 |
| 240S8 | 14.8 | TST | Chamalea_gallina | 91 | 14.47 | 12.11 | 2.03 | 123.65 | 0 | 0 |
| 240S8 | 14.8 | TST | Chamalea_gallina | 92 | 13.22 | 11.48 | 1.92 | 108.98 | 0 | 0 |
| 240S8 | 14.8 | TST | Chamalea_gallina | 93 | 6.94 | 6.21 | 0.91 | 30.96 | 0 | 0 |
| 240S8 | 14.8 | TST | Chamalea_gallina | 94 | 7.18 | 6.44 | 1.06 | 33.36 | 0 | 0 |
| 240S8 | 14.8 | TST | Chamalea_gallina | 95 | 7.1 | 6.36 | 1.2 | 32.4 | 0 | 0 |
| 240S8 | 14.8 | TST | Chamalea_gallina | 96 | 6.59 | 5.8 | 0.81 | 27.72 | 0 | 0 |
| 240S8 | 14.8 | TST | Chamalea_gallina | 97 | 6.7 | 5.82 | 0.96 | 27.47 | 0 | 0 |
| 240S8 | 14.8 | TST | Chamalea_gallina | 98 | 8.86 | 7.93 | 1.24 | 50.9 | 0 | 0 |
| 240S8 | 14.8 | TST | Chamalea_gallina | 99 | 6.94 | 6.26 | 0.92 | 31.01 | 0 | 0 |
| 240S8 | 14.8 | TST | Chamalea_gallina | 100 | 7.04 | 6.14 | 0.85 | 30.78 | 0 | 0 |
| 240S8 | 14.8 | TST | Chamalea_gallina | 101 | 6.51 | 5.74 | 0.96 | 26.73 | 0 | 0 |
| 240S8 | 14.8 | TST | Chamalea_gallina | 102 | 6.42 | 5.69 | 0.95 | 25.96 | 0 | 0 |
| 240S8 | 14.8 | TST | Chamalea_gallina | 103 | 6.94 | 6.12 | 1.14 | 30.53 | 0 | 0 |
| 240S8 | 14.8 | TST | Chamalea_gallina | 104 | 6.69 | 5.81 | 0.97 | 27.36 | 0 | 0 |
| 240S8 | 14.8 | TST | Chamalea_gallina | 105 | 6.8 | 5.93 | 0.7 | 28.36 | 0 | 0 |
| 240S8 | 14.8 | TST | Chamalea_gallina | 106 | 6.81 | 5.82 | 0.99 | 28.08 | 0 | 0 |
| 240S8 | 14.8 | TST | Chamalea_gallina | 107 | 7.59 | 6.79 | 1.34 | 36.53 | 0 | 0 |
| 240S8 | 14.8 | TST | Chamalea_gallina | 108 | 6.29 | 5.49 | 0.9 | 24.86 | 0 | 0 |
| 240S8 | 14.8 | TST | Chamalea_gallina | 109 | 9.96 | 8.84 | 1.32 | 63.79 | 0 | 0 |
| 240S8 | 14.8 | TST | Chamalea_gallina | 110 | 9.14 | 8.4 | 1.31 | 55.4 | 0 | 0 |
| 240S8 | 14.8 | TST | Chamalea_gallina | 111 | 8.72 | 8.03 | 1.37 | 50.14 | 0 | 0 |
| 240S8 | 14.8 | TST | Chamalea_gallina | 112 | 8.76 | 7.34 | 1.23 | 45.87 | 0 | 0 |
| 240S8 | 14.8 | TST | Chamalea_gallina | 113 | 8.03 | 7.29 | 0.91 | 42.17 | 0 | 0 |
| 240S8 | 14.8 | TST | Chamalea_gallina | 114 | 9.71 | 8.35 | 1.19 | 58.37 | 0 | 0 |
| 240S8 | 14.8 | TST | Chamalea_gallina | 115 | 6 | 5.43 | 0.74 | 23.74 | 0 | 0 |
| 240S8 | 14.8 | TST | Chamalea_gallina | 116 | 10.26 | 9.22 | 1.34 | 67.33 | 0 | 0 |
| 240S8 | 14.8 | TST | Chamalea_gallina | 117 | 9.85 | 8.65 | 1.34 | 60.97 | 0 | 0 |
| 240S8 | 14.8 | TST | Chamalea_gallina | 119 | 10.29 | 8.76 | 1.09 | 65.1 | 0 | 0 |
| 240S8 | 14.8 | TST | Chamalea_gallina | 120 | 10.3 | 8.86 | 1.23 | 65.15 | 0 | 0 |
| 240S8 | 14.8 | TST | Chamalea_gallina | 121 | 10.71 | 9.43 | 1.37 | 73.16 | 0 | 0 |
| 240S8 | 14.8 | TST | Chamalea_gallina | 122 | 9.26 | 8.09 | 1.2 | 53.78 | 0 | 0 |
| 240S8 | 14.8 | TST | Chamalea_gallina | 123 | 9.83 | 8.71 | 0.87 | 61.8 | 0 | 0 |
| 240S8 | 14.8 | TST | Chamalea_gallina | 124 | 10.6 | 9.4 | 1.61 | 73.17 | 0 | 0 |
| 240S8 | 14.8 | TST | Chamalea_gallina | 125 | 10.67 | 9.11 | 1.28 | 69.71 | 0 | 0 |
| 240S8 | 14.8 | TST | Chamalea_gallina | 126 | 6.74 | 5.72 | 1.09 | 27.77 | 0 | 0 |
| 240S8 | 14.8 | TST | Chamalea_gallina | 127 | 8.44 | 7.43 | 0.73 | 45.41 | 0 | 0 |
| 240S8 | 14.8 | TST | Chamalea_gallina | 128 | 7.43 | 6.67 | 1.12 | 35.51 | 0 | 0 |
| 240S8 | 14.8 | TST | Chamalea_gallina | 129 | 7.38 | 6.32 | 0.99 | 33 | 0 | 0 |
| 240S8 | 14.8 | TST | Chamalea_gallina | 130 | 8.69 | 7.63 | 0.94 | 47.3 | 0 | 0 |
| 240S8 | 14.8 | TST | Chamalea_gallina | 131 | 9.86 | 8.46 | 1.35 | 59.79 | 0 | 0 |
| 240S8 | 14.8 | TST | Chamalea_gallina | 132 | 9.26 | 7.92 | 1.1 | 52.39 | 0 | 0 |
| 240S8 | 14.8 | TST | Chamalea_gallina | 133 | 10.7 | 9 | 1.23 | 68.78 | 0 | 0 |
| 240S8 | 14.8 | TST | Chamalea_gallina | 134 | 10.4 | 9.06 | 1.23 | 67.82 | 0 | 0 |
| 240S8 | 14.8 | TST | Chamalea_gallina | 135 | 9.81 | 8.78 | 1.4 | 62.92 | 0 | 0 |
| 240S8 | 14.8 | TST | Chamalea_gallina | 136 | 9.74 | 8.67 | 1.16 | 60.59 | 0 | 0 |
| 240S8 | 14.8 | TST | Chamalea_gallina | 137 | 9.55 | 8.43 | 1.14 | 57.61 | 0 | 0 |
| 240S8 | 14.8 | TST | Chamalea_gallina | 138 | 7.69 | 6.79 | 1.2 | 36.82 | 0 | 0 |
| 240S8 | 14.8 | TST | Chamalea_gallina | 139 | 9.45 | 8.35 | 0.96 | 56.66 | 0 | 0 |
| 240S8 | 14.8 | TST | Chamalea_gallina | 140 | 9.1 | 7.8 | 0.94 | 51.84 | 0 | 0 |
| 240S8 | 14.8 | TST | Chamalea_gallina | 141 | 10.04 | 9.5 | 1.44 | 68.76 | 0 | 0 |
| 240S8 | 14.8 | TST | Chamalea_gallina | 142 | 8.6 | 7.47 | 1.29 | 45.57 | 0 | 0 |
| 240S8 | 14.8 | TST | Chamalea_gallina | 143 | 7.16 | 6.58 | 0.99 | 33.87 | 0 | 0 |
| 240S8 | 14.8 | TST | Chamalea_gallina | 144 | 8.65 | 7.09 | 1.17 | 44.09 | 0 | 0 |
| 240S8 | 14.8 | TST | Chamalea_gallina | 145 | 7.88 | 7.08 | 0.96 | 39.63 | 0 | 0 |
| 240S8 | 14.8 | TST | Chamalea_gallina | 146 | 8.16 | 7.26 | 0.92 | 42.34 | 0 | 0 |
| 240S8 | 14.8 | TST | Chamalea_gallina | 147 | 10.31 | 9.35 | 1.26 | 69.44 | 0 | 0 |
| 240S8 | 14.8 | TST | Chamalea_gallina | 148 | 8.75 | 7.35 | 1.17 | 46.11 | 0 | 0 |
| 240S8 | 14.8 | TST | Chamalea_gallina | 149 | 9.8 | 8.53 | 1.43 | 60.19 | 0 | 0 |
| 240S8 | 14.8 | TST | Chamalea_gallina | 150 | 9.81 | 8.43 | 1 | 58.17 | 0 | 0 |
| 240S8 | 14.8 | TST | Chamalea_gallina | 151 | 9.31 | 8.26 | 1.16 | 56.44 | 0 | 0 |
| 240S8 | 14.8 | TST | Chamalea_gallina | 152 | 12.68 | 11.23 | 1.4 | 102.72 | 0 | 0 |
| 240S8 | 14.8 | TST | Chamalea_gallina | 153 | 8.46 | 7.18 | 1.18 | 43.3 | 0 | 0 |
| 240S8 | 14.8 | TST | Chamalea_gallina | 154 | 9.93 | 9.17 | 1.4 | 64.92 | 0 | 0 |
| 240S8 | 14.8 | TST | Chamalea_gallina | 155 | 13.99 | 12.14 | 1.87 | 120.5 | 0 | 0 |
| 240S8 | 14.8 | TST | Chamalea_gallina | 156 | 6.82 | 6.09 | 0.84 | 29.74 | 0 | 0 |
| 240S8 | 14.8 | TST | Chamalea_gallina | 157 | 10.61 | 9.18 | 1.05 | 70.36 | 0 | 0 |
| 240S8 | 14.8 | TST | Chamalea_gallina | 158 | 11.9 | 10.2 | 1.51 | 87.22 | 0 | 0 |
| 240S8 | 14.8 | TST | Chamalea_gallina | 159 | 10.3 | 8.84 | 1.46 | 65.72 | 0 | 0 |
| 240S8 | 14.8 | TST | Chamalea_gallina | 160 | 13.84 | 12.5 | 2.37 | 126.78 | 0 | 0 |
| 240S8 | 14.8 | TST | Chamalea_gallina | 161 | 8.01 | 7.13 | 1.21 | 41.35 | 0 | 0 |
| 240S8 | 14.8 | TST | Chamalea_gallina | 162 | 9.59 | 8.51 | 1.21 | 58.08 | 0 | 0 |
| 240S8 | 14.8 | TST | Chamalea_gallina | 163 | 7.56 | 6.58 | 1.03 | 35.43 | 0 | 0 |
| 240S8 | 14.8 | TST | Chamalea_gallina | 164 | 8.31 | 7.32 | 1.28 | 42.99 | 0 | 0 |
| 240S8 | 14.8 | TST | Chamalea_gallina | 165 | 7.66 | 6.7 | 1.01 | 37.3 | 0 | 0 |
| 240S8 | 14.8 | TST | Chamalea_gallina | 166 | 6.06 | 5.51 | 0.91 | 24.02 | 0 | 0 |
| 240S8 | 14.8 | TST | Chamalea_gallina | 167 | 7.59 | 6.8 | 1.13 | 37.33 | 0 | 0 |
| 240S8 | 14.8 | TST | Chamalea_gallina | 168 | 6.32 | 5.78 | 0.84 | 26.01 | 0 | 0 |
| 240S8 | 14.8 | TST | Chamalea_gallina | 169 | 6.98 | 6.24 | 0.93 | 31.56 | 0 | 0 |
| 240S8 | 14.8 | TST | Chamalea_gallina | 170 | 7.76 | 6.55 | 1.1 | 36.58 | 0 | 0 |
| 240S8 | 14.8 | TST | Chamalea_gallina | 171 | 6.76 | 5.85 | 0.8 | 28.64 | 0 | 0 |
| 240S8 | 14.8 | TST | Chamalea_gallina | 172 | 8 | 7.1 | 0.97 | 40.45 | 0 | 0 |
| 240S8 | 14.8 | TST | Chamalea_gallina | 173 | 8 | 7.25 | 1.2 | 41.67 | 0 | 0 |
| 240S8 | 14.8 | TST | Chamalea_gallina | 174 | 6.84 | 6.01 | 0.7 | 29.32 | 0 | 0 |
| 240S8 | 14.8 | TST | Chamalea_gallina | 175 | 6.38 | 6.03 | 0.76 | 27.18 | 0 | 0 |
| 240S8 | 14.8 | TST | Chamalea_gallina | 176 | 7.63 | 6.88 | 0.99 | 37.22 | 0 | 0 |
| 240S8 | 14.8 | TST | Chamalea_gallina | 177 | 10.13 | 9 | 1.66 | 64.8 | 0 | 0 |
| 240S8 | 14.8 | TST | Chamalea_gallina | 178 | 6.88 | 6.21 | 0.87 | 30.77 | 0 | 0 |
| 240S8 | 14.8 | TST | Chamalea_gallina | 179 | 7.11 | 6.4 | 1.02 | 32.48 | 0 | 0 |
| 240S8 | 14.8 | TST | Chamalea_gallina | 180 | 7.74 | 6.92 | 0.8 | 38.72 | 0 | 0 |
| 240S8 | 14.8 | TST | Chamalea_gallina | 181 | 8.55 | 7.83 | 1.11 | 48.21 | 0 | 0 |
| 240S8 | 14.8 | TST | Chamalea_gallina | 182 | 8.47 | 7.52 | 0.92 | 46.11 | 0 | 0 |
| 240S8 | 14.8 | TST | Chamalea_gallina | 183 | 6.46 | 5.99 | 0.9 | 27.28 | 0 | 0 |
| 240S8 | 14.8 | TST | Chamalea_gallina | 184 | 7.23 | 6.93 | 1.14 | 35.3 | 0 | 0 |
| 240S8 | 14.8 | TST | Chamalea_gallina | 185 | 6.55 | 5.96 | 1.08 | 27.89 | 0 | 0 |
| 240S8 | 14.8 | TST | Chamalea_gallina | 186 | 7.83 | 7.05 | 1.06 | 38.84 | 0 | 0 |
| 240S8 | 14.8 | TST | Chamalea_gallina | 187 | 9.46 | 8.61 | 1.23 | 58.19 | 0 | 0 |
| 240S8 | 14.8 | TST | Chamalea_gallina | 188 | 10.69 | 9.38 | 1.4 | 71.54 | 0 | 0 |
| 240S8 | 14.8 | TST | Chamalea_gallina | 189 | 8.32 | 7.45 | 0.9 | 43.85 | 0 | 0 |
| 240S8 | 14.8 | TST | Chamalea_gallina | 190 | 8.99 | 7.98 | 1.22 | 51.37 | 0 | 0 |
| 240S8 | 14.8 | TST | Chamalea_gallina | 191 | 7.22 | 6.51 | 0.75 | 33.87 | 0 | 0 |
| 240S8 | 14.8 | TST | Chamalea_gallina | 192 | 7.07 | 6.42 | 0.83 | 32.57 | 0 | 0 |
| 240S8 | 14.8 | TST | Chamalea_gallina | 193 | 7.7 | 6.84 | 1.3 | 38.8 | 0 | 0 |
| 240S8 | 14.8 | TST | Chamalea_gallina | 194 | 6.61 | 5.88 | 0.78 | 27.61 | 0 | 0 |
| 240S8 | 14.8 | TST | Chamalea_gallina | 195 | 7.44 | 6.7 | 0.91 | 35.67 | 0 | 0 |
| 240S8 | 14.8 | TST | Chamalea_gallina | 196 | 8.82 | 7.65 | 1.29 | 48.28 | 0 | 0 |
| 240S8 | 14.8 | TST | Chamalea_gallina | 197 | 7.58 | 6.96 | 1 | 37.73 | 0 | 0 |
| 240S8 | 14.8 | TST | Chamalea_gallina | 198 | 9.14 | 8.44 | 0.9 | 55.07 | 0 | 0 |
| 240S8 | 14.8 | TST | Chamalea_gallina | 199 | 10.27 | 9.15 | 1.11 | 67.79 | 0 | 0 |
| 240S8 | 14.8 | TST | Chamalea_gallina | 200 | 7.71 | 6.82 | 1.29 | 37.87 | 0 | 0 |
| 240S8 | 14.8 | TST | Chamalea_gallina | 201 | 7.6 | 6.73 | 0.88 | 36.81 | 0 | 0 |
| 240S8 | 14.8 | TST | Chamalea_gallina | 202 | 8.12 | 7.65 | 1.25 | 44.49 | 0 | 0 |
| 240S8 | 14.8 | TST | Chamalea_gallina | 203 | 8.15 | 7.34 | 0.95 | 42.71 | 0 | 0 |
| 240S8 | 14.8 | TST | Chamalea_gallina | 204 | 8.41 | 7.74 | 1.13 | 47.16 | 0 | 0 |
| 240S8 | 14.8 | TST | Chamalea_gallina | 205 | 6.39 | 5.9 | 0.69 | 26.92 | 0 | 0 |
| 240S8 | 14.8 | TST | Chamalea_gallina | 206 | 12.25 | 10.89 | 1.45 | 96.14 | 0 | 0 |
| 240S8 | 14.8 | TST | Chamalea_gallina | 207 | 11.33 | 11.34 | 1.49 | 91.84 | 0 | 0 |
| 240S8 | 14.8 | TST | Chamalea_gallina | 208 | 8.99 | 8.17 | 1.31 | 52.45 | 0 | 0 |
| 240S8 | 14.8 | TST | Chamalea_gallina | 209 | 9.27 | 8.09 | 0.99 | 53.42 | 0 | 0 |
| 240S8 | 14.8 | TST | Chamalea_gallina | 210 | 8.3 | 7.51 | 0.92 | 44.23 | 0 | 0 |
| 240S8 | 14.8 | TST | Chamalea_gallina | 211 | 9.39 | 8.26 | 1.31 | 55.6 | 0 | 0 |
| 240S8 | 14.8 | TST | Chamalea_gallina | 212 | 8.25 | 7.06 | 1.24 | 40.99 | 0 | 0 |
| 240S8 | 14.8 | TST | Chamalea_gallina | 213 | 9.6 | 8.59 | 1.34 | 59.25 | 0 | 0 |
| 240S8 | 14.8 | TST | Chamalea_gallina | 214 | 8.64 | 7.84 | 1.03 | 49.09 | 0 | 0 |
| 240S8 | 14.8 | TST | Chamalea_gallina | 215 | 8.05 | 7.12 | 1.1 | 41.41 | 0 | 0 |
| 240S8 | 14.8 | TST | Chamalea_gallina | 216 | 9.48 | 8.53 | 1.08 | 58.06 | 0 | 0 |
| 240S8 | 14.8 | TST | Chamalea_gallina | 217 | 9.6 | 8.13 | 1.22 | 56.17 | 0 | 0 |
| 240S8 | 14.8 | TST | Chamalea_gallina | 218 | 8.09 | 7.3 | 0.84 | 41.87 | 0 | 0 |
| 240S8 | 14.8 | TST | Chamalea_gallina | 219 | 10.1 | 9.08 | 1.63 | 66.24 | 0 | 0 |
| 240S8 | 14.8 | TST | Chamalea_gallina | 220 | 7.62 | 6.99 | 1.14 | 38.55 | 0 | 0 |
| 240S8 | 14.8 | TST | Chamalea_gallina | 221 | 9.79 | 8.6 | 1.36 | 60.07 | 0 | 0 |
| 240S8 | 14.8 | TST | Chamalea_gallina | 222 | 10.12 | 9.07 | 1.24 | 66.49 | 0 | 0 |
| 240S8 | 14.8 | TST | Chamalea_gallina | 223 | 9.77 | 8.67 | 1.22 | 60.66 | 0 | 0 |
| 240S8 | 14.8 | TST | Chamalea_gallina | 224 | 9.16 | 7.9 | 1.21 | 51.83 | 0 | 0 |
| 240S8 | 14.8 | TST | Chamalea_gallina | 225 | 10.33 | 8.83 | 1.36 | 66.39 | 0 | 0 |
| 240S8 | 14.8 | TST | Chamalea_gallina | 226 | 11.97 | 10.27 | 1.56 | 88.8 | 0 | 0 |
| 240S8 | 14.8 | TST | Chamalea_gallina | 227 | 14.13 | 12.24 | 2.04 | 125.48 | 0 | 0 |
| 240S8 | 14.8 | TST | Chamalea_gallina | 228 | 10.05 | 8.28 | 1.57 | 59.76 | 0 | 0 |
| 240S8 | 14.8 | TST | Chamalea_gallina | 229 | 12.14 | 10.4 | 1.66 | 91.59 | 0 | 0 |
| 240S8 | 14.8 | TST | Chamalea_gallina | 230 | 6.5 | 5.82 | 0.83 | 27.38 | 0 | 0 |
| 240S8 | 14.8 | TST | Chamalea_gallina | 231 | 10.71 | 8.97 | 1.55 | 68.87 | 0 | 0 |
| 240S8 | 14.8 | TST | Chamalea_gallina | 232 | 11.03 | 9.82 | 1.65 | 77.83 | 0 | 0 |
| 240S8 | 14.8 | TST | Chamalea_gallina | 233 | 9.71 | 8.4 | 1.24 | 59.35 | 0 | 0 |
| 240S8 | 14.8 | TST | Chamalea_gallina | 234 | 9.52 | 8.24 | 1.21 | 57.91 | 0 | 0 |
| 240S8 | 14.8 | TST | Chamalea_gallina | 235 | 11.97 | 10.88 | 1.39 | 93.44 | 0 | 0 |
| 240S8 | 14.8 | TST | Chamalea_gallina | 236 | 10.12 | 9.28 | 1.45 | 67.34 | 0 | 0 |
| 240S8 | 14.8 | TST | Chamalea_gallina | 237 | 10.83 | 10.08 | 1.24 | 78.37 | 0 | 0 |
| 240S8 | 14.8 | TST | Chamalea_gallina | 238 | 11.37 | 9.76 | 1.09 | 80.16 | 0 | 0 |
| 240S8 | 14.8 | TST | Chamalea_gallina | 239 | 11.36 | 10.01 | 1.33 | 82.14 | 0 | 0 |
| 240S8 | 14.8 | TST | Chamalea_gallina | 240 | 8.89 | 7.97 | 1.16 | 50.97 | 0 | 0 |
| 240S8 | 14.8 | TST | Chamalea_gallina | 241 | 11.54 | 10.34 | 1.73 | 86.97 | 0 | 0 |
| 240S8 | 14.8 | TST | Chamalea_gallina | 242 | 13.18 | 11.49 | 2.04 | 107.59 | 0 | 0 |
| 240S8 | 14.8 | TST | Chamalea_gallina | 243 | 11.34 | 10.08 | 1.37 | 82.89 | 0 | 0 |
| 240S8 | 14.8 | TST | Chamalea_gallina | 244 | 9.66 | 8.8 | 1.49 | 61.16 | 0 | 0 |
| 240S8 | 14.8 | TST | Chamalea_gallina | 245 | 9.61 | 8.7 | 1.21 | 60.91 | 0 | 0 |
| 240S8 | 14.8 | TST | Chamalea_gallina | 246 | 10.91 | 9.8 | 1.46 | 76.72 | 0 | 0 |
| 240S8 | 14.8 | TST | Chamalea_gallina | 247 | 9.73 | 8.67 | 1.28 | 61.86 | 0 | 0 |
| 240S8 | 14.8 | TST | Chamalea_gallina | 248 | 6.24 | 5.78 | 0.74 | 25.68 | 0 | 0 |
| 240S8 | 14.8 | TST | Chamalea_gallina | 249 | 10.96 | 9.84 | 1.67 | 78.72 | 0 | 0 |
| 240S8 | 12.5 | TST | Chamalea_gallina | 1 | 3.67 | 3.31 | 0.38 | 8.71 | 0 | 0 |
| 240S8 | 12.5 | TST | Chamalea_gallina | 2 | 4.04 | 3.66 | 0.46 | 10.61 | 0 | 0 |
| 240S8 | 12.5 | TST | Chamalea_gallina | 3 | 3.58 | 3.22 | 0.4 | 8.2 | 0 | 0 |
| 240S8 | 12.5 | TST | Chamalea_gallina | 4 | 3.61 | 3.37 | 0.44 | 8.62 | 0 | 0 |
| 240S8 | 12.5 | TST | Chamalea_gallina | 5 | 4.22 | 3.97 | 0.36 | 11.84 | 0 | 0 |
| 240S8 | 12.5 | TST | Chamalea_gallina | 6 | 3.88 | 3.59 | 0.49 | 9.95 | 0 | 0 |
| 240S8 | 12.5 | TST | Chamalea_gallina | 7 | 4.38 | 3.93 | 0.64 | 12.47 | 0 | 0 |
| 240S8 | 12.5 | TST | Chamalea_gallina | 8 | 4.55 | 4.27 | 0.58 | 13.97 | 0 | 0 |
| 240S8 | 12.5 | TST | Chamalea_gallina | 9 | 5.37 | 4.81 | 0.73 | 18.36 | 0 | 0 |
| 240S8 | 12.5 | TST | Chamalea_gallina | 10 | 5.5 | 4.84 | 0.97 | 18.79 | 0 | 0 |
| 240S8 | 12.5 | TST | Chamalea_gallina | 11 | 5.47 | 4.84 | 0.67 | 19.24 | 0 | 0 |
| 240S8 | 12.5 | TST | Chamalea_gallina | 12 | 5.54 | 4.81 | 0.67 | 19.12 | 0 | 0 |
| 240S8 | 12.5 | TST | Chamalea_gallina | 13 | 5.65 | 5.07 | 0.99 | 20.48 | 0 | 0 |
| 240S8 | 12.5 | TST | Chamalea_gallina | 14 | 5.75 | 5.08 | 0.75 | 20.7 | 0 | 0 |
| 240S8 | 12.5 | TST | Chamalea_gallina | 15 | 6.05 | 5.34 | 0.76 | 23.4 | 1 | 3 |
| 240S8 | 12.5 | TST | Chamalea_gallina | 16 | 6.5 | 5.44 | 0.9 | 25.15 | 1 | 1 |
| 240S8 | 12.5 | TST | Chamalea_gallina | 17 | 6.2 | 5.67 | 0.63 | 25.15 | 0 | 0 |
| 240S8 | 12.5 | TST | Chamalea_gallina | 18 | 6.66 | 5.95 | 0.87 | 28.7 | 0 | 0 |
| 240S8 | 12.5 | TST | Chamalea_gallina | 19 | 6.79 | 6.08 | 0.97 | 29.46 | 0 | 0 |
| 240S8 | 12.5 | TST | Chamalea_gallina | 20 | 7.12 | 6.42 | 1.08 | 32.45 | 0 | 0 |
| 240S8 | 12.5 | TST | Chamalea_gallina | 21 | 7.8 | 6.88 | 1.01 | 38.3 | 0 | 0 |
| 240S8 | 12.5 | TST | Chamalea_gallina | 22 | 9.45 | 8.44 | 1.19 | 56.57 | 0 | 0 |
| 240S8 | 12.5 | TST | Chamalea_gallina | 23 | 10.37 | 9.54 | 1.52 | 70.08 | 0 | 0 |
| 240S8 | 12.5 | TST | Chamalea_gallina | 24 | 10.5 | 9.02 | 1.47 | 68.4 | 0 | 0 |
| 240S8 | 12.5 | TST | Chamalea_gallina | 25 | 10.22 | 8.82 | 1.51 | 65.45 | 0 | 0 |
| 240S8 | 12.5 | TST | Chamalea_gallina | 26 | 10.47 | 9.38 | 1.59 | 69.77 | 0 | 0 |
| 240S8 | 12.5 | TST | Chamalea_gallina | 27 | 10.87 | 9.69 | 1.36 | 75.27 | 0 | 0 |
| 240S8 | 12.5 | TST | Chamalea_gallina | 28 | 18.6 | 17.15 | 1.96 | 225.59 | 1 | 9 |
| 240S8 | 12.5 | TST | Chamalea_gallina | 29 | 19.94 | 17.26 | 2.67 | 249.38 | 1 | 8 |
| 240S8 | 13.1 | TST | Chamalea_gallina | 1 | 6.53 | 5.58 | 1.09 | 26.58 | 0 | 0 |
| 240S8 | 13.1 | TST | Chamalea_gallina | 2 | 10.56 | 9.35 | 1.47 | 70.26 | 1 | 5 |
| 240S8 | 13.1 | TST | Chamalea_gallina | 3 | 12.01 | 11.12 | 1.57 | 96.95 | 1 | 3 |
| 240S8 | 13.1 | TST | Chamalea_gallina | 4 | 12.09 | 10.46 | 1.76 | 92.81 | 1 | 1 |
| 240S8 | 13.1 | TST | Chamalea_gallina | 5 | 12.43 | 10.86 | 1.74 | 97.65 | 0 | 0 |
| 240S8 | 13.1 | TST | Chamalea_gallina | 6 | 12.93 | 11.29 | 2.22 | 105.44 | 0 | 0 |
| 240S8 | 13.1 | TST | Chamalea_gallina | 7 | 14.82 | 13.36 | 2.08 | 141.56 | 1 | 1 |
| 240S8 | 13.1 | TST | Chamalea_gallina | 8 | 12.37 | 11.26 | 1.63 | 99.79 | 0 | 0 |
| 240S8 | 13.1 | TST | Chamalea_gallina | 9 | 12.18 | 10.51 | 1.49 | 90.41 | 1 | 1 |
| 240S8 | 13.1 | TST | Chamalea_gallina | 10 | 9.62 | 8.78 | 1.24 | 60.03 | 1 | 2 |
| 240S8 | 13.1 | TST | Chamalea_gallina | 11 | 11.32 | 10.05 | 1.76 | 81.9 | 0 | 0 |
| 240S8 | 13.1 | TST | Chamalea_gallina | 12 | 11.88 | 10.5 | 1.69 | 89.18 | 0 | 0 |
| 240S8 | 13.1 | TST | Chamalea_gallina | 13 | 11.09 | 10.09 | 1.74 | 80.76 | 1 | 2 |
| 240S8 | 13.1 | TST | Chamalea_gallina | 14 | 11.24 | 9.9 | 1.58 | 79.75 | 1 | 7 |
| 240S8 | 13.1 | TST | Chamalea_gallina | 15 | 10.98 | 9.57 | 1.6 | 76.63 | 1 | 2 |
| 240S8 | 13.1 | TST | Chamalea_gallina | 16 | 11.06 | 9.75 | 1.6 | 78.8 | 0 | 0 |
| 240S8 | 13.1 | TST | Chamalea_gallina | 17 | 13.68 | 11.78 | 1.71 | 115.61 | 1 | 2 |
| 240S8 | 13.1 | TST | Chamalea_gallina | 18 | 13.37 | 12.09 | 2.02 | 116.35 | 0 | 0 |
| 240S8 | 13.1 | TST | Chamalea_gallina | 19 | 13.59 | 11.87 | 1.2 | 116.68 | 0 | 0 |
| 240S8 | 13.1 | TST | Chamalea_gallina | 20 | 11.59 | 9.96 | 1.63 | 83.44 | 1 | 1 |
| 240S8 | 13.1 | TST | Chamalea_gallina | 21 | 9.96 | 8.98 | 1.46 | 65.15 | 0 | 0 |
| 240S8 | 13.1 | TST | Chamalea_gallina | 22 | 12.63 | 11.07 | 1.71 | 100.89 | 1 | 1 |
| 240S8 | 13.1 | TST | Chamalea_gallina | 23 | 12.46 | 11.4 | 1.94 | 101.74 | 1 | 3 |
| 240S8 | 13.1 | TST | Chamalea_gallina | 24 | 6.28 | 5.61 | 0.92 | 26.17 | 0 | 0 |
| 240S8 | 13.1 | TST | Chamalea_gallina | 25 | 11.41 | 9.64 | 1.46 | 77.58 | 0 | 0 |
| 240S8 | 13.1 | TST | Chamalea_gallina | 26 | 13.77 | 12.49 | 1.88 | 123.72 | 1 | 5 |
| 240S8 | 13.1 | TST | Chamalea_gallina | 27 | 11.45 | 10.56 | 1.59 | 88.12 | 1 | 1 |
| 240S8 | 13.1 | TST | Chamalea_gallina | 28 | 14.52 | 12.46 | 2.55 | 132.57 | 1 | 1 |
| 240S8 | 13.1 | TST | Chamalea_gallina | 29 | 13.67 | 11.53 | 1.86 | 113.2 | 0 | 0 |
| 240S8 | 13.1 | TST | Chamalea_gallina | 30 | 12.67 | 11.02 | 1.85 | 99.85 | 1 | 1 |
| 240S8 | 13.1 | TST | Chamalea_gallina | 31 | 10.85 | 9.71 | 1.39 | 74.65 | 1 | 1 |
| 240S8 | 13.1 | TST | Chamalea_gallina | 32 | 11.81 | 10.68 | 1.61 | 90.15 | 0 | 0 |
| 240S8 | 13.1 | TST | Chamalea_gallina | 33 | 11.02 | 9.37 | 1.55 | 74.38 | 1 | 1 |
| 240S8 | 13.1 | TST | Chamalea_gallina | 34 | 13.66 | 11.64 | 2.05 | 114.73 | 0 | 0 |
| 240S8 | 13.1 | TST | Chamalea_gallina | 35 | 15.05 | 13.3 | 2.37 | 145.06 | 1 | 10 |
| 240S8 | 13.1 | TST | Chamalea_gallina | 36 | 7.88 | 7.65 | 1.42 | 43.29 | 0 | 0 |
| 240S8 | 13.1 | TST | Chamalea_gallina | 37 | 7.85 | 7.15 | 1.04 | 39.92 | 0 | 0 |
| 240S8 | 13.1 | TST | Chamalea_gallina | 38 | 10.21 | 9.39 | 1.46 | 69.21 | 1 | 1 |
| 240S8 | 13.1 | TST | Chamalea_gallina | 39 | 6.64 | 6.03 | 0.84 | 28.33 | 1 | 1 |
| 240S8 | 13.1 | TST | Chamalea_gallina | 40 | 6.51 | 5.96 | 1.11 | 28.03 | 0 | 0 |
| 240S8 | 13.1 | TST | Chamalea_gallina | 41 | 6.78 | 6.01 | 1.07 | 28.93 | 0 | 0 |
| 240S8 | 13.1 | TST | Chamalea_gallina | 42 | 7.6 | 6.81 | 1.22 | 36.88 | 0 | 0 |
| 240S8 | 13.1 | TST | Chamalea_gallina | 43 | 7.27 | 6.81 | 1.07 | 34.98 | 0 | 0 |
| 240S8 | 13.1 | TST | Chamalea_gallina | 44 | 10.78 | 9.68 | 1.42 | 75.57 | 0 | 0 |
| 240S8 | 13.1 | TST | Chamalea_gallina | 45 | 11.45 | 10.31 | 1.39 | 85.69 | 0 | 0 |
| 240S8 | 13.1 | TST | Chamalea_gallina | 46 | 6 | 5.5 | 0.96 | 23.42 | 0 | 0 |
| 240S8 | 13.1 | TST | Chamalea_gallina | 47 | 6.81 | 6.14 | 0.88 | 29.69 | 0 | 0 |
| 240S8 | 13.1 | TST | Chamalea_gallina | 48 | 7.01 | 6.13 | 1.14 | 30.8 | 0 | 0 |
| 240S8 | 13.1 | TST | Chamalea_gallina | 49 | 6.49 | 5.84 | 0.81 | 27.64 | 0 | 0 |
| 240S8 | 13.1 | TST | Chamalea_gallina | 50 | 7.49 | 6.6 | 1.09 | 35.31 | 0 | 0 |
| 240S8 | 13.1 | TST | Chamalea_gallina | 51 | 6.79 | 6.01 | 0.88 | 29.23 | 0 | 0 |
| 240S8 | 13.1 | TST | Chamalea_gallina | 52 | 6.39 | 5.71 | 0.73 | 26.03 | 0 | 0 |
| 240S8 | 13.1 | TST | Chamalea_gallina | 53 | 7 | 6.12 | 0.94 | 31.13 | 0 | 0 |
| 240S8 | 13.1 | TST | Chamalea_gallina | 54 | 6.59 | 5.93 | 1.02 | 27.92 | 0 | 0 |
| 240S8 | 13.1 | TST | Chamalea_gallina | 55 | 6.83 | 6.31 | 0.86 | 30.8 | 0 | 0 |
| 240S8 | 13.1 | TST | Chamalea_gallina | 56 | 9.05 | 7.95 | 1.16 | 51.67 | 0 | 0 |
| 240S8 | 13.1 | TST | Chamalea_gallina | 57 | 5.82 | 5.28 | 0.78 | 21.84 | 1 | 1 |
| 240S8 | 13.1 | TST | Chamalea_gallina | 58 | 7.5 | 6.9 | 0.95 | 37.38 | 0 | 0 |
| 240S8 | 13.1 | TST | Chamalea_gallina | 59 | 8.08 | 7.28 | 0.95 | 42.62 | 1 | 4 |
| 240S8 | 13.1 | TST | Chamalea_gallina | 60 | 9.47 | 8.21 | 1.15 | 56.5 | 1 | 3 |
| 240S8 | 13.1 | TST | Chamalea_gallina | 61 | 10.24 | 9.29 | 1.44 | 68.96 | 1 | 2 |
| 240S8 | 13.1 | TST | Chamalea_gallina | 62 | 8.46 | 7.58 | 1.06 | 45.87 | 0 | 0 |
| 240S8 | 13.1 | TST | Chamalea_gallina | 63 | 9.08 | 8.4 | 1.17 | 54.92 | 1 | 3 |
| 240S8 | 13.1 | TST | Chamalea_gallina | 64 | 10.95 | 9.59 | 1.66 | 75.9 | 0 | 0 |
| 240S8 | 13.1 | TST | Chamalea_gallina | 65 | 7.68 | 6.84 | 1.18 | 37.57 | 0 | 0 |
| 240S8 | 13.1 | TST | Chamalea_gallina | 66 | 6.38 | 5.71 | 0.85 | 25.84 | 0 | 0 |
| 240S8 | 13.1 | TST | Chamalea_gallina | 67 | 7.25 | 6.28 | 0.83 | 33.34 | 0 | 0 |
| 240S8 | 13.1 | TST | Chamalea_gallina | 68 | 7.75 | 6.87 | 1.16 | 38.33 | 0 | 0 |
| 240S8 | 13.1 | TST | Chamalea_gallina | 69 | 6.93 | 6.19 | 0.93 | 30.88 | 0 | 0 |
| 240S8 | 13.1 | TST | Chamalea_gallina | 70 | 6.79 | 5.95 | 1.03 | 29.32 | 0 | 0 |
| 240S8 | 13.1 | TST | Chamalea_gallina | 71 | 6.5 | 5.84 | 0.96 | 27.37 | 0 | 0 |
| 240S8 | 13.1 | TST | Chamalea_gallina | 72 | 7.79 | 6.85 | 1.17 | 38.58 | 0 | 0 |
| 240S8 | 13.1 | TST | Chamalea_gallina | 73 | 8.32 | 7.42 | 1.22 | 44 | 0 | 0 |
| 240S8 | 13.1 | TST | Chamalea_gallina | 74 | 9.56 | 8.35 | 1.16 | 57.25 | 0 | 0 |
| 240S8 | 13.1 | TST | Chamalea_gallina | 75 | 6.71 | 5.87 | 1.18 | 28.18 | 0 | 0 |
| 240S8 | 13.1 | TST | Chamalea_gallina | 76 | 6.77 | 6.33 | 1.26 | 30.78 | 0 | 0 |
| 240S8 | 13.1 | TST | Chamalea_gallina | 77 | 7.25 | 6.51 | 1 | 33.73 | 0 | 0 |
| 240S8 | 13.1 | TST | Chamalea_gallina | 78 | 6.85 | 6.18 | 1.09 | 30.84 | 0 | 0 |
| 240S8 | 13.1 | TST | Chamalea_gallina | 79 | 6.91 | 6.06 | 0.91 | 29.75 | 1 | 1 |
| 240S8 | 13.1 | TST | Chamalea_gallina | 80 | 6.53 | 5.79 | 1.12 | 27.19 | 0 | 0 |
| 240S8 | 13.1 | TST | Chamalea_gallina | 81 | 9.67 | 8.62 | 1.24 | 60.95 | 0 | 0 |
| 240S8 | 13.1 | TST | Chamalea_gallina | 82 | 10.39 | 9.34 | 1.35 | 68.99 | 0 | 0 |
| 240S8 | 13.1 | TST | Chamalea_gallina | 83 | 9.14 | 8.34 | 1.11 | 54.99 | 0 | 0 |
| 240S8 | 13.1 | TST | Chamalea_gallina | 84 | 7 | 5.91 | 0.99 | 30.12 | 0 | 0 |
| 240S8 | 13.1 | TST | Chamalea_gallina | 85 | 8.23 | 7.49 | 1.34 | 44.86 | 0 | 0 |
| 240S8 | 13.1 | TST | Chamalea_gallina | 86 | 8 | 6.85 | 1.08 | 39.37 | 0 | 0 |
| 240S8 | 13.1 | TST | Chamalea_gallina | 87 | 6.72 | 6.1 | 1.05 | 29.68 | 0 | 0 |
| 240S8 | 13.1 | TST | Chamalea_gallina | 88 | 8.65 | 7.7 | 1.27 | 47.83 | 0 | 0 |
| 240S8 | 13.1 | TST | Chamalea_gallina | 89 | 6.67 | 6.12 | 1.08 | 29.12 | 0 | 0 |
| 240S8 | 13.1 | TST | Chamalea_gallina | 90 | 6.73 | 5.89 | 0.89 | 27.48 | 0 | 0 |
| 240S8 | 13.1 | TST | Chamalea_gallina | 91 | 7.75 | 6.68 | 0.98 | 37.74 | 0 | 0 |
| 240S8 | 13.1 | TST | Chamalea_gallina | 92 | 8.35 | 7.48 | 1.3 | 45.73 | 1 | 1 |
| 240S8 | 13.1 | TST | Chamalea_gallina | 93 | 7.5 | 6.5 | 1.09 | 34.37 | 0 | 0 |
| 240S8 | 13.1 | TST | Chamalea_gallina | 94 | 7.8 | 7.13 | 1.03 | 40.03 | 0 | 0 |
| 240S8 | 13.1 | TST | Chamalea_gallina | 95 | 7.4 | 6.48 | 1.1 | 33.61 | 0 | 0 |
| 240S8 | 13.1 | TST | Chamalea_gallina | 96 | 9.13 | 7.97 | 1.32 | 52.55 | 1 | 3 |
| 240S8 | 13.1 | TST | Chamalea_gallina | 97 | 11.42 | 9.73 | 1.27 | 80.75 | 1 | 3 |
| 240S8 | 13.1 | TST | Chamalea_gallina | 98 | 8.21 | 7.41 | 1.24 | 44.43 | 0 | 0 |
| 240S8 | 13.1 | TST | Chamalea_gallina | 99 | 9.76 | 8.71 | 1.26 | 59.89 | 0 | 0 |
| 240S8 | 13.1 | TST | Chamalea_gallina | 100 | 7.23 | 6.39 | 0.81 | 33.06 | 0 | 0 |
| 240S8 | 13.1 | TST | Chamalea_gallina | 101 | 7.56 | 6.36 | 1.2 | 34.28 | 0 | 0 |
| 240S8 | 13.1 | TST | Chamalea_gallina | 102 | 9.25 | 8.26 | 1.5 | 56.01 | 0 | 0 |
| 240S8 | 13.1 | TST | Chamalea_gallina | 103 | 9.24 | 8.05 | 1.39 | 52.98 | 0 | 0 |
| 240S8 | 13.1 | TST | Chamalea_gallina | 104 | 11.29 | 9.53 | 1.57 | 78.08 | 1 | 1 |
| 240S8 | 13.1 | TST | Chamalea_gallina | 105 | 11.06 | 9.89 | 0.79 | 78.76 | 0 | 0 |
| 240S8 | 13.1 | TST | Chamalea_gallina | 106 | 7.42 | 6.31 | 1.09 | 34.06 | 0 | 0 |
| 240S8 | 13.1 | TST | Chamalea_gallina | 107 | 8.41 | 7.79 | 1.42 | 46.88 | 0 | 0 |
| 240S8 | 13.1 | TST | Chamalea_gallina | 108 | 9.2 | 7.88 | 1.44 | 52.28 | 0 | 0 |
| 240S8 | 13.1 | TST | Chamalea_gallina | 109 | 8.94 | 7.85 | 1.5 | 50.4 | 0 | 0 |
| 240S8 | 13.1 | TST | Chamalea_gallina | 110 | 8.84 | 7.55 | 1.14 | 48.71 | 1 | 7 |
| 240S8 | 13.1 | TST | Chamalea_gallina | 111 | 8.7 | 7.62 | 1.13 | 46.39 | 0 | 0 |
| 240S8 | 13.1 | TST | Chamalea_gallina | 112 | 6.34 | 5.73 | 0.95 | 25.88 | 0 | 0 |
| 240S8 | 13.1 | TST | Chamalea_gallina | 113 | 9.36 | 8.12 | 1.11 | 53.37 | 0 | 0 |
| 240S8 | 13.1 | TST | Chamalea_gallina | 114 | 6.44 | 5.9 | 0.77 | 27.42 | 0 | 0 |
| 240S8 | 13.1 | TST | Chamalea_gallina | 115 | 8.36 | 7.27 | 1.45 | 44.42 | 1 | 7 |
| 240S8 | 13.1 | TST | Chamalea_gallina | 116 | 9.58 | 8.78 | 1.4 | 60.22 | 1 | 4 |
| 240S8 | 13.1 | TST | Chamalea_gallina | 117 | 7.47 | 6.43 | 1.41 | 34.16 | 1 | 1 |
| 240S8 | 13.1 | TST | Chamalea_gallina | 118 | 7.19 | 6.3 | 1.1 | 32.33 | 0 | 0 |
| 240S8 | 13.1 | TST | Chamalea_gallina | 119 | 7.54 | 6.75 | 1.09 | 36.78 | 0 | 0 |
| 240S8 | 13.1 | TST | Chamalea_gallina | 120 | 7.7 | 6.88 | 1.01 | 38.07 | 0 | 0 |
| 240S8 | 13.1 | TST | Chamalea_gallina | 121 | 8.96 | 8.43 | 1.09 | 53.52 | 0 | 0 |
| 240S8 | 13.1 | TST | Chamalea_gallina | 122 | 11.99 | 10.8 | 1.59 | 93.4 | 1 | 1 |
| 240S8 | 13.1 | TST | Chamalea_gallina | 123 | 7.39 | 6.52 | 0.96 | 34.45 | 0 | 0 |
| 240S8 | 13.1 | TST | Chamalea_gallina | 124 | 6.61 | 5.93 | 0.81 | 27.52 | 0 | 0 |
| 240S8 | 13.1 | TST | Chamalea_gallina | 125 | 6.65 | 6.02 | 1.08 | 28.88 | 1 | 5 |
| 240S8 | 13.1 | TST | Chamalea_gallina | 126 | 11.12 | 10 | 1.42 | 79.51 | 1 | 1 |
| 240S8 | 13.1 | TST | Chamalea_gallina | 127 | 9.44 | 8.08 | 1.52 | 54.37 | 0 | 0 |
| 240S8 | 13.1 | TST | Chamalea_gallina | 128 | 9.15 | 8.38 | 1.31 | 55.58 | 1 | 5 |
| 240S8 | 13.1 | TST | Chamalea_gallina | 129 | 8.97 | 7.96 | 1.44 | 51.7 | 1 | 4 |
| 240S8 | 13.1 | TST | Chamalea_gallina | 130 | 8.54 | 7.69 | 1.42 | 46.63 | 0 | 0 |
| 240S8 | 13.1 | TST | Chamalea_gallina | 131 | 6.46 | 5.57 | 0.85 | 25.4 | 0 | 0 |
| 240S8 | 13.1 | TST | Chamalea_gallina | 132 | 6.65 | 5.68 | 0.77 | 26.66 | 0 | 0 |
| 240S8 | 13.1 | TST | Chamalea_gallina | 133 | 7.96 | 6.79 | 1.24 | 39.22 | 0 | 0 |
| 240S8 | 13.1 | TST | Chamalea_gallina | 134 | 8.48 | 7.54 | 1.42 | 45.94 | 0 | 0 |
| 240S8 | 13.1 | TST | Chamalea_gallina | 135 | 7.93 | 7.08 | 1.08 | 39.58 | 0 | 0 |
| 240S8 | 13.1 | TST | Chamalea_gallina | 136 | 8.92 | 7.81 | 1.13 | 50.37 | 0 | 0 |
| 240S8 | 13.1 | TST | Chamalea_gallina | 137 | 9.83 | 8.89 | 1.56 | 63.62 | 1 | 4 |
| 240S8 | 13.1 | TST | Chamalea_gallina | 138 | 6.51 | 5.97 | 0.77 | 28.22 | 1 | 4 |
| 240S8 | 13.1 | TST | Chamalea_gallina | 139 | 7.77 | 6.75 | 1.36 | 37.52 | 0 | 0 |
| 240S8 | 13.1 | TST | Chamalea_gallina | 140 | 8.66 | 7.37 | 1.27 | 45.89 | 0 | 0 |
| 240S8 | 13.1 | TST | Chamalea_gallina | 141 | 6.28 | 5.68 | 0.84 | 25.78 | 1 | 2 |
| 240S8 | 13.1 | TST | Chamalea_gallina | 142 | 7.8 | 6.86 | 1.01 | 37.82 | 0 | 0 |
| 240S8 | 13.1 | TST | Chamalea_gallina | 143 | 6.91 | 6.07 | 1.09 | 30.54 | 0 | 0 |
| 240S8 | 13.1 | TST | Chamalea_gallina | 144 | 6.59 | 5.76 | 0.8 | 27.07 | 0 | 0 |
| 240S8 | 13.1 | TST | Chamalea_gallina | 145 | 6.28 | 5.68 | 0.95 | 25.34 | 0 | 0 |
| 240S8 | 13.1 | TST | Chamalea_gallina | 146 | 9.88 | 8.49 | 1.41 | 59.9 | 0 | 0 |
| 240S8 | 13.1 | TST | Chamalea_gallina | 147 | 8.59 | 7.35 | 1.53 | 45.83 | 0 | 0 |
| 240S8 | 13.1 | TST | Chamalea_gallina | 148 | 9.98 | 8.39 | 1.5 | 59.53 | 1 | 3 |
| 240S8 | 13.1 | TST | Chamalea_gallina | 149 | 8.58 | 7.63 | 1.28 | 46.37 | 0 | 0 |
| 240S8 | 13.1 | TST | Chamalea_gallina | 150 | 8.37 | 7.15 | 1.34 | 43.07 | 0 | 0 |
| 240S8 | 13.1 | TST | Chamalea_gallina | 151 | 8.03 | 7.08 | 1.14 | 40.71 | 0 | 0 |
| 240S8 | 13.1 | TST | Chamalea_gallina | 152 | 8.49 | 7.76 | 1.2 | 47.56 | 0 | 0 |
| 240S8 | 13.1 | TST | Chamalea_gallina | 153 | 8.44 | 7.58 | 1.2 | 45.77 | 0 | 0 |
| 240S8 | 13.1 | TST | Chamalea_gallina | 154 | 8.8 | 8.11 | 1.27 | 51.55 | 1 | 1 |
| 240S8 | 13.1 | TST | Chamalea_gallina | 155 | 9.24 | 8.17 | 1.32 | 54.68 | 0 | 0 |
| 240S8 | 13.1 | TST | Chamalea_gallina | 156 | 8.25 | 7.55 | 1.34 | 45.29 | 0 | 0 |
| 240S8 | 13.1 | TST | Chamalea_gallina | 157 | 8.7 | 7.72 | 1.37 | 48.68 | 0 | 0 |
| 240S8 | 13.1 | TST | Chamalea_gallina | 158 | 8.87 | 8.09 | 1.16 | 51.71 | 1 | 1 |
| 240S8 | 13.1 | TST | Chamalea_gallina | 159 | 9.82 | 8.64 | 1.23 | 61.02 | 0 | 0 |
| 240S8 | 13.1 | TST | Chamalea_gallina | 160 | 9 | 7.93 | 1.37 | 51.15 | 1 | 1 |
| 240S8 | 13.1 | TST | Chamalea_gallina | 161 | 9.5 | 8.77 | 1.35 | 60.81 | 1 | 2 |
| 240S8 | 13.1 | TST | Chamalea_gallina | 162 | 10.27 | 9.07 | 1.44 | 66.83 | 0 | 0 |
| 240S8 | 13.1 | TST | Chamalea_gallina | 163 | 9.55 | 8.62 | 1.4 | 59.1 | 0 | 0 |
| 240S8 | 13.1 | TST | Chamalea_gallina | 164 | 12.38 | 10.97 | 1.94 | 99 | 0 | 0 |
| 240S8 | 13.1 | TST | Chamalea_gallina | 165 | 11 | 9.64 | 1.56 | 75.67 | 1 | 3 |
| 240S8 | 13.1 | TST | Chamalea_gallina | 166 | 12.01 | 10.4 | 1.39 | 91.32 | 0 | 0 |
| 240S8 | 13.1 | TST | Chamalea_gallina | 167 | 12.92 | 11.38 | 2.24 | 106.92 | 0 | 0 |
| 240S8 | 13.1 | TST | Chamalea_gallina | 168 | 12.9 | 10.81 | 1.73 | 99.59 | 1 | 3 |
| 240S8 | 13.1 | TST | Chamalea_gallina | 169 | 14.86 | 13.59 | 2.07 | 148.18 | 1 | 4 |
| 240S8 | 13.1 | TST | Chamalea_gallina | 170 | 16.94 | 15.6 | 2.54 | 190.57 | 1 | 5 |
| 240S8 | 13.1 | TST | Chamalea_gallina | 171 | 18.31 | 15.17 | 2.39 | 203.34 | 0 | 0 |
| 240S8 | 13.1 | TST | Chamalea_gallina | 172 | 17.87 | 15.76 | 2.5 | 202.24 | 0 | 0 |
| 240S8 | 13.1 | TST | Chamalea_gallina | 173 | 17.39 | 15.22 | 2.52 | 193.21 | 1 | 10 |
| 240S8 | 13.1 | TST | Chamalea_gallina | 174 | 18.08 | 16.03 | 2.41 | 208.94 | 0 | 0 |
| 240S8 | 13.1 | TST | Chamalea_gallina | 175 | 16.88 | 15.47 | 2.3 | 188.69 | 1 | 1 |
| 240S8 | 13.1 | TST | Chamalea_gallina | 176 | 17.53 | 15 | 2.32 | 190.19 | 0 | 0 |
| 240S8 | 13.1 | TST | Chamalea_gallina | 177 | 18.39 | 16.61 | 2.51 | 215.96 | 1 | 5 |
| 240S8 | 13.1 | TST | Chamalea_gallina | 178 | 16.83 | 14.74 | 2.14 | 177.84 | 0 | 0 |
| 240S8 | 13.1 | TST | Chamalea_gallina | 179 | 15.67 | 13.56 | 2.06 | 152.71 | 1 | 2 |
| 240S8 | 13.1 | TST | Chamalea_gallina | 180 | 15.55 | 13.77 | 2.43 | 155.7 | 0 | 0 |
| 240S8 | 13.1 | TST | Chamalea_gallina | 181 | 18 | 15.31 | 2.44 | 199.18 | 1 | 11 |
| 240S8 | 13.1 | TST | Chamalea_gallina | 182 | 17.72 | 15.13 | 2.23 | 192.4 | 1 | 5 |
| 240S8 | 13.1 | TST | Chamalea_gallina | 183 | 17.5 | 14.86 | 2.58 | 188 | 1 | 4 |
| 240S8 | 13.1 | TST | Chamalea_gallina | 184 | 16.98 | 15.15 | 1.85 | 186.96 | 1 | 16 |
| 240S8 | 13.1 | TST | Chamalea_gallina | 185 | 15.98 | 14.14 | 2.69 | 163.71 | 0 | 0 |
| 240S8 | 13.1 | TST | Chamalea_gallina | 186 | 16.28 | 13.93 | 2.35 | 163.15 | 1 | 1 |
| 240S8 | 13.1 | TST | Chamalea_gallina | 187 | 17.91 | 15.3 | 2.29 | 198.24 | 1 | 3 |
| 240S8 | 13.1 | TST | Chamalea_gallina | 188 | 15.41 | 13.37 | 2.06 | 149.72 | 1 | 3 |
| 240S8 | 13.1 | TST | Chamalea_gallina | 189 | 17.86 | 15.68 | 2.36 | 201.76 | 1 | 9 |
| 240S8 | 13.1 | TST | Chamalea_gallina | 190 | 17.47 | 15.15 | 2.38 | 191.43 | 1 | 3 |
| 240S8 | 13.1 | TST | Chamalea_gallina | 191 | 15.31 | 13.31 | 2.07 | 146.24 | 1 | 7 |
| 240S8 | 13.1 | TST | Chamalea_gallina | 192 | 17.22 | 15.26 | 2.52 | 189.82 | 1 | 1 |
| 240S8 | 13.1 | TST | Chamalea_gallina | 193 | 18.13 | 15.78 | 2.52 | 207.52 | 1 | 18 |
| 240S8 | 13.1 | TST | Chamalea_gallina | 194 | 18.39 | 15.2 | 2.24 | 204.36 | 1 | 6 |
| 240S8 | 13.1 | TST | Chamalea_gallina | 195 | 18.13 | 15.95 | 2.51 | 207.42 | 1 | 4 |
| 240S8 | 13.1 | TST | Chamalea_gallina | 196 | 17.19 | 15.39 | 2.1 | 191.8 | 1 | 12 |
| 240S8 | 13.1 | TST | Chamalea_gallina | 197 | 18.21 | 15.9 | 2.19 | 211.09 | 1 | 22 |
| 240S8 | 13.1 | TST | Chamalea_gallina | 198 | 15.36 | 13.47 | 2.11 | 150.27 | 0 | 0 |
| 240S8 | 13.1 | TST | Chamalea_gallina | 199 | 16.6 | 14.4 | 2.53 | 171.57 | 0 | 0 |
| 240S8 | 13.1 | TST | Chamalea_gallina | 200 | 5.85 | 5.2 | 0.75 | 21.4 | 0 | 0 |
| 240S8 | 13.1 | TST | Chamalea_gallina | 201 | 6.36 | 5.43 | 0.8 | 24.74 | 0 | 0 |
| 240S8 | 13.1 | TST | Chamalea_gallina | 202 | 5.66 | 5.07 | 0.76 | 20.53 | 0 | 0 |
| 240S8 | 13.1 | TST | Chamalea_gallina | 203 | 5.64 | 5.13 | 0.84 | 20.76 | 0 | 0 |
| 240S8 | 13.1 | TST | Chamalea_gallina | 204 | 6.09 | 5.39 | 1.06 | 24.03 | 0 | 0 |
| 240S8 | 13.1 | TST | Chamalea_gallina | 205 | 6.45 | 5.68 | 0.89 | 26.41 | 0 | 0 |
| 240S8 | 13.1 | TST | Chamalea_gallina | 206 | 5.73 | 5.21 | 0.86 | 21.55 | 1 | 1 |
| 240S8 | 13.1 | TST | Chamalea_gallina | 207 | 5.53 | 4.99 | 0.86 | 19.75 | 0 | 0 |
| 240S8 | 13.1 | TST | Chamalea_gallina | 208 | 5.21 | 4.64 | 0.76 | 17.52 | 0 | 0 |
| 240S8 | 13.1 | TST | Chamalea_gallina | 209 | 6.07 | 5.53 | 0.77 | 23.88 | 0 | 0 |
| 240S8 | 13.1 | TST | Chamalea_gallina | 210 | 5.9 | 5.47 | 1.07 | 23.4 | 1 | 1 |
| 240S8 | 13.1 | TST | Chamalea_gallina | 211 | 5.51 | 4.88 | 0.81 | 19.69 | 1 | 1 |
| 240S8 | 13.1 | TST | Chamalea_gallina | 212 | 5.66 | 5.12 | 0.97 | 21.06 | 0 | 0 |
| 240S8 | 13.1 | TST | Chamalea_gallina | 213 | 6.37 | 5.46 | 0.98 | 25.14 | 1 | 2 |
| 240S8 | 13.1 | TST | Chamalea_gallina | 214 | 5.53 | 4.9 | 0.9 | 19.78 | 0 | 0 |
| 240S8 | 13.1 | TST | Chamalea_gallina | 215 | 5.78 | 5.21 | 0.76 | 21.3 | 0 | 0 |
| 240S8 | 13.1 | TST | Chamalea_gallina | 216 | 5.71 | 5.13 | 0.9 | 20.96 | 0 | 0 |
| 240S8 | 13.1 | TST | Chamalea_gallina | 217 | 6.11 | 5.32 | 0.8 | 23.47 | 0 | 0 |
| 240S8 | 13.1 | TST | Chamalea_gallina | 218 | 5.26 | 4.6 | 0.69 | 17.3 | 0 | 0 |
| 240S8 | 13.1 | TST | Chamalea_gallina | 219 | 6.15 | 5.54 | 1.03 | 24.67 | 0 | 0 |
| 240S8 | 13.1 | TST | Chamalea_gallina | 220 | 6.24 | 5.43 | 0.97 | 24.63 | 0 | 0 |
| 240S8 | 13.1 | TST | Chamalea_gallina | 221 | 5.83 | 5.06 | 0.74 | 21.17 | 0 | 0 |
| 240S8 | 13.1 | TST | Chamalea_gallina | 222 | 5.06 | 4.64 | 0.6 | 16.89 | 0 | 0 |
| 240S8 | 13.1 | TST | Chamalea_gallina | 223 | 5.21 | 4.79 | 0.75 | 17.83 | 0 | 0 |
| 240S8 | 13.1 | TST | Chamalea_gallina | 224 | 5.63 | 5.04 | 0.69 | 19.99 | 0 | 0 |
| 240S8 | 13.1 | TST | Chamalea_gallina | 225 | 5.76 | 5.19 | 0.61 | 21.47 | 0 | 0 |
| 240S8 | 13.1 | TST | Chamalea_gallina | 226 | 5.26 | 4.87 | 0.82 | 18.4 | 0 | 0 |
| 240S8 | 13.1 | TST | Chamalea_gallina | 227 | 5.38 | 4.72 | 0.91 | 18.23 | 0 | 0 |
| 240S8 | 13.1 | TST | Chamalea_gallina | 228 | 5.37 | 4.84 | 0.76 | 18.42 | 0 | 0 |
| 240S8 | 13.1 | TST | Chamalea_gallina | 229 | 5.44 | 4.96 | 0.7 | 19.16 | 0 | 0 |
| 240S8 | 13.1 | TST | Chamalea_gallina | 230 | 6.01 | 5.3 | 0.72 | 22.54 | 0 | 0 |
| 240S8 | 13.1 | TST | Chamalea_gallina | 231 | 5.65 | 4.92 | 0.81 | 19.99 | 0 | 0 |
| 240S8 | 13.1 | TST | Chamalea_gallina | 232 | 5.68 | 5 | 0.85 | 20.46 | 0 | 0 |
| 240S8 | 13.1 | TST | Chamalea_gallina | 233 | 5.63 | 4.94 | 0.86 | 20.31 | 0 | 0 |
| 240S8 | 13.1 | TST | Chamalea_gallina | 234 | 5.75 | 5.09 | 0.57 | 20.88 | 0 | 0 |
| 240S8 | 13.1 | TST | Chamalea_gallina | 235 | 5.54 | 5.01 | 0.81 | 19.76 | 0 | 0 |
| 240S8 | 13.1 | TST | Chamalea_gallina | 236 | 5.2 | 4.78 | 0.69 | 18.1 | 0 | 0 |
| 240S8 | 13.1 | TST | Chamalea_gallina | 237 | 5.4 | 4.82 | 0.79 | 18.88 | 1 | 1 |
| 240S8 | 13.1 | TST | Chamalea_gallina | 238 | 5.82 | 5.19 | 1.06 | 21.77 | 1 | 2 |
| 240S8 | 13.1 | TST | Chamalea_gallina | 239 | 5.6 | 4.93 | 0.66 | 20.05 | 0 | 0 |
| 240S8 | 13.1 | TST | Chamalea_gallina | 240 | 4.91 | 4.33 | 0.52 | 14.9 | 0 | 0 |
| 240S8 | 13.1 | TST | Chamalea_gallina | 241 | 4.68 | 4.22 | 0.56 | 13.85 | 0 | 0 |
| 240S8 | 13.1 | TST | Chamalea_gallina | 242 | 5.02 | 4.4 | 0.56 | 15.48 | 0 | 0 |
| 240S8 | 13.1 | TST | Chamalea_gallina | 243 | 5.32 | 4.88 | 0.86 | 18.93 | 0 | 0 |
| 240S8 | 13.1 | TST | Chamalea_gallina | 244 | 5.26 | 4.59 | 0.82 | 17.7 | 0 | 0 |
| 240S8 | 13.1 | TST | Chamalea_gallina | 245 | 5.27 | 4.7 | 0.66 | 18.01 | 0 | 0 |
| 240S8 | 13.1 | TST | Chamalea_gallina | 246 | 5.21 | 4.5 | 0.67 | 17.04 | 0 | 0 |
| 240S8 | 13.1 | TST | Chamalea_gallina | 247 | 5.41 | 4.7 | 0.63 | 18.37 | 0 | 0 |
| 240S8 | 13.1 | TST | Chamalea_gallina | 248 | 4.86 | 4.53 | 0.68 | 15.88 | 0 | 0 |
| 240S8 | 13.1 | TST | Chamalea_gallina | 249 | 5.44 | 4.75 | 0.75 | 18.55 | 0 | 0 |
| 240S8 | 13.1 | TST | Chamalea_gallina | 250 | 5.28 | 4.61 | 0.56 | 17.54 | 0 | 0 |
| 240S8 | 13.1 | TST | Chamalea_gallina | 251 | 5.09 | 4.5 | 0.62 | 15.8 | 0 | 0 |
| 240S8 | 13.1 | TST | Chamalea_gallina | 252 | 4.55 | 4.01 | 0.49 | 12.98 | 0 | 0 |
| 240S8 | 13.1 | TST | Chamalea_gallina | 253 | 5.02 | 4.43 | 0.68 | 15.97 | 0 | 0 |
| 240S8 | 13.1 | TST | Chamalea_gallina | 254 | 5.07 | 4.68 | 0.49 | 17.07 | 0 | 0 |
| 240S8 | 13.1 | TST | Chamalea_gallina | 255 | 5.22 | 4.54 | 0.66 | 16.76 | 0 | 0 |
| 240S8 | 13.1 | TST | Chamalea_gallina | 256 | 4.57 | 4.06 | 0.52 | 13.06 | 0 | 0 |
| 240S8 | 13.1 | TST | Chamalea_gallina | 257 | 5.51 | 4.97 | 0.8 | 19.4 | 0 | 0 |
| 240S8 | 13.1 | TST | Chamalea_gallina | 258 | 4.82 | 4.26 | 0.55 | 14.79 | 0 | 0 |
| 240S8 | 13.1 | TST | Chamalea_gallina | 259 | 3.87 | 3.5 | 0.45 | 9.94 | 0 | 0 |
| 240S8 | 13.1 | TST | Chamalea_gallina | 260 | 4.82 | 4.24 | 0.65 | 14.64 | 0 | 0 |
| 240S8 | 13.1 | TST | Chamalea_gallina | 261 | 4.36 | 3.9 | 0.61 | 12.33 | 0 | 0 |
| 240S8 | 13.1 | TST | Chamalea_gallina | 262 | 4.77 | 4.19 | 0.52 | 14.2 | 0 | 0 |
| 240S8 | 13.1 | TST | Chamalea_gallina | 263 | 5.02 | 4.46 | 0.79 | 15.99 | 0 | 0 |
| 240S8 | 13.1 | TST | Chamalea_gallina | 264 | 4.72 | 4.19 | 0.73 | 14.35 | 1 | 1 |
| 240S8 | 13.1 | TST | Chamalea_gallina | 265 | 3.77 | 3.34 | 0.39 | 8.87 | 0 | 0 |
| 240S8 | 13.1 | TST | Chamalea_gallina | 266 | 4.47 | 4.09 | 0.54 | 12.85 | 0 | 0 |
| 240S8 | 13.1 | TST | Chamalea_gallina | 267 | 5.54 | 4.89 | 0.84 | 19.68 | 1 | 2 |
| 240S8 | 13.1 | TST | Chamalea_gallina | 268 | 5 | 4.43 | 0.6 | 15.84 | 0 | 0 |
| 240S8 | 13.1 | TST | Chamalea_gallina | 269 | 4.79 | 4.3 | 0.64 | 14.95 | 0 | 0 |
| 240S8 | 13.1 | TST | Chamalea_gallina | 270 | 4.92 | 4.21 | 0.59 | 14.57 | 0 | 0 |
| 240S8 | 13.1 | TST | Chamalea_gallina | 271 | 4.69 | 4.37 | 0.63 | 14.41 | 0 | 0 |
| 240S8 | 13.1 | TST | Chamalea_gallina | 272 | 4.54 | 3.84 | 0.63 | 12.77 | 0 | 0 |
| 240S8 | 13.1 | TST | Chamalea_gallina | 273 | 3.96 | 3.49 | 0.53 | 9.84 | 0 | 0 |
| 240S8 | 13.1 | TST | Chamalea_gallina | 274 | 3.82 | 3.38 | 0.43 | 9.31 | 0 | 0 |
| 240S8 | 13.1 | TST | Chamalea_gallina | 275 | 4.74 | 4.35 | 0.76 | 14.89 | 0 | 0 |
| 240S8 | 13.1 | TST | Chamalea_gallina | 276 | 4.88 | 4.15 | 0.8 | 14.75 | 1 | 1 |
| 240S8 | 13.1 | TST | Chamalea_gallina | 277 | 4.66 | 4.05 | 0.55 | 13.64 | 0 | 0 |
| 240S8 | 13.1 | TST | Chamalea_gallina | 278 | 3.71 | 3.38 | 0.53 | 8.79 | 0 | 0 |
| 240S8 | 13.1 | TST | Chamalea_gallina | 279 | 3.93 | 3.52 | 0.51 | 10.02 | 0 | 0 |
| 240S8 | 13.1 | TST | Chamalea_gallina | 280 | 3.55 | 3.06 | 0.56 | 7.74 | 0 | 0 |
| 240S8 | 13.1 | TST | Chamalea_gallina | 281 | 3.59 | 3.27 | 0.51 | 8.24 | 0 | 0 |
| 240S8 | 13.1 | TST | Chamalea_gallina | 282 | 4.89 | 4.44 | 0.8 | 15.64 | 0 | 0 |
| 240S8 | 13.1 | TST | Chamalea_gallina | 283 | 3.94 | 3.97 | 0.67 | 11.6 | 0 | 0 |
| 240S8 | 13.1 | TST | Chamalea_gallina | 284 | 4.42 | 4 | 0.65 | 13.02 | 0 | 0 |
| 240S8 | 13.1 | TST | Chamalea_gallina | 285 | 4.96 | 4.29 | 0.64 | 15.4 | 0 | 0 |
| 240S8 | 13.1 | TST | Chamalea_gallina | 286 | 3.26 | 2.9 | 0.44 | 7 | 0 | 0 |
| 240S8 | 13.1 | TST | Chamalea_gallina | 287 | 4.88 | 4.63 | 0.78 | 16.29 | 1 | 1 |
| 240S8 | 13.1 | TST | Chamalea_gallina | 288 | 4.23 | 3.66 | 0.59 | 11.15 | 0 | 0 |
| 240S8 | 13.1 | TST | Chamalea_gallina | 289 | 4.33 | 3.95 | 0.62 | 12.2 | 0 | 0 |
| 240S8 | 13.1 | TST | Chamalea_gallina | 290 | 4.88 | 4.49 | 0.74 | 15.65 | 0 | 0 |
| 240S8 | 13.1 | TST | Chamalea_gallina | 291 | 3.46 | 2.97 | 0.42 | 7.35 | 0 | 0 |
| 240S8 | 13.1 | TST | Chamalea_gallina | 292 | 3.88 | 3.5 | 0.46 | 10.09 | 1 | 1 |
| 240S8 | 13.1 | TST | Chamalea_gallina | 293 | 4.09 | 3.75 | 0.56 | 10.98 | 0 | 0 |
| 240S8 | 13.1 | TST | Chamalea_gallina | 294 | 3.68 | 3.38 | 0.53 | 9.06 | 0 | 0 |
| 240S8 | 13.1 | TST | Chamalea_gallina | 295 | 4.36 | 3.87 | 0.61 | 12.3 | 0 | 0 |
| 240S8 | 13.1 | TST | Chamalea_gallina | 296 | 3.57 | 3.14 | 0.46 | 8.08 | 0 | 0 |
| 240S8 | 13.1 | TST | Chamalea_gallina | 297 | 4.63 | 4.14 | 0.64 | 13.26 | 0 | 0 |
| 240S8 | 13.1 | TST | Chamalea_gallina | 298 | 3.44 | 3.11 | 0.6 | 7.59 | 0 | 0 |
| 240S8 | 13.1 | TST | Chamalea_gallina | 299 | 4.76 | 4.34 | 0.61 | 14.69 | 0 | 0 |
| 240S8 | 13.1 | TST | Chamalea_gallina | 300 | 4.31 | 3.88 | 0.49 | 12.04 | 0 | 0 |
| 240S8 | 13.1 | TST | Chamalea_gallina | 301 | 3.8 | 3.42 | 0.59 | 9.28 | 0 | 0 |
| 240S8 | 13.1 | TST | Chamalea_gallina | 302 | 3.65 | 3.33 | 0.53 | 8.75 | 0 | 0 |
| 240S8 | 13.1 | TST | Chamalea_gallina | 303 | 4 | 3.49 | 0.56 | 10.2 | 0 | 0 |
| 240S8 | 13.1 | TST | Chamalea_gallina | 304 | 4.41 | 3.92 | 0.53 | 12.19 | 0 | 0 |
| 240S8 | 13.1 | TST | Chamalea_gallina | 305 | 4.07 | 3.7 | 0.53 | 10.83 | 0 | 0 |
| 240S8 | 13.1 | TST | Chamalea_gallina | 306 | 4.28 | 3.64 | 0.28 | 11.34 | 0 | 0 |
| 240S8 | 13.1 | TST | Chamalea_gallina | 307 | 4.09 | 3.66 | 0.6 | 10.67 | 0 | 0 |
| 240S8 | 13.1 | TST | Chamalea_gallina | 308 | 4.28 | 3.75 | 0.46 | 11.34 | 0 | 0 |
| 240S8 | 13.1 | TST | Chamalea_gallina | 309 | 4.6 | 4.16 | 0.6 | 14.05 | 0 | 0 |
| 240S8 | 13.1 | TST | Chamalea_gallina | 310 | 3.65 | 3.19 | 0.63 | 8.56 | 1 | 1 |
| 240S8 | 13.1 | TST | Chamalea_gallina | 311 | 3.45 | 3.07 | 0.44 | 7.67 | 0 | 0 |
| 240S8 | 13.1 | TST | Chamalea_gallina | 312 | 4.64 | 4.15 | 0.58 | 13.72 | 0 | 0 |
| 240S8 | 13.1 | TST | Chamalea_gallina | 313 | 4.53 | 4.18 | 0.91 | 13.66 | 0 | 0 |
| 240S8 | 13.1 | TST | Chamalea_gallina | 314 | 3.72 | 3.33 | 0.47 | 8.89 | 0 | 0 |
| 240S8 | 13.1 | TST | Chamalea_gallina | 315 | 4.14 | 3.83 | 0.53 | 11.35 | 0 | 0 |
| 240S8 | 13.1 | TST | Chamalea_gallina | 316 | 3.73 | 3.38 | 0.63 | 9.19 | 0 | 0 |
| 240S8 | 13.1 | TST | Chamalea_gallina | 317 | 4.22 | 3.8 | 0.67 | 11.48 | 1 | 1 |
| 240S8 | 13.1 | TST | Chamalea_gallina | 318 | 3.82 | 3.47 | 0.47 | 9.37 | 0 | 0 |
| 240S8 | 13.1 | TST | Chamalea_gallina | 319 | 4.28 | 3.79 | 0.68 | 11.88 | 0 | 0 |
| 240S8 | 13.1 | TST | Chamalea_gallina | 320 | 4.45 | 3.98 | 0.6 | 12.75 | 0 | 0 |
| 240S8 | 13.1 | TST | Chamalea_gallina | 321 | 3.31 | 3.07 | 0.48 | 7.29 | 0 | 0 |
| 240S8 | 13.1 | TST | Chamalea_gallina | 322 | 4.33 | 3.85 | 0.65 | 11.98 | 0 | 0 |
| 240S8 | 13.1 | TST | Chamalea_gallina | 323 | 4.13 | 3.61 | 0.54 | 10.87 | 0 | 0 |
| 240S8 | 13.1 | TST | Chamalea_gallina | 324 | 18.12 | 15.95 | 2.59 | 207.9 | 1 | 8 |
| 240S8 | 13.1 | TST | Chamalea_gallina | 325 | 21.58 | 18.5 | 2.55 | 287.83 | 0 | 0 |
| 240S8 | 13.1 | TST | Chamalea_gallina | 326 | 19.8 | 17.51 | 2.94 | 252.9 | 0 | 0 |
| 240S8 | 13.1 | TST | Chamalea_gallina | 327 | 21.53 | 19.13 | 3.18 | 298.08 | 1 | 12 |
| 240S8 | 13.1 | TST | Chamalea_gallina | 328 | 22.82 | 20.13 | 3.3 | 330.99 | 0 | 0 |
| 240S8 | 13.1 | TST | Chamalea_gallina | 329 | 18.02 | 16.07 | 2.49 | 211.2 | 0 | 0 |
| 240S8 | 13.1 | TST | Chamalea_gallina | 330 | 21.41 | 18.05 | 2.89 | 277.56 | 1 | 5 |
| 240S8 | 13.1 | TST | Chamalea_gallina | 331 | 18.79 | 16.54 | 2.48 | 221.55 | 1 | 9 |
| 240S8 | 13.1 | TST | Chamalea_gallina | 332 | 19.98 | 17.24 | 2.52 | 249.28 | 1 | 35 |
| 240S8 | 13.1 | TST | Chamalea_gallina | 333 | 19.46 | 17.73 | 2.48 | 247.93 | 1 | 14 |
| 240S8 | 13.1 | TST | Chamalea_gallina | 334 | 18.89 | 17 | 2.97 | 232.83 | 1 | 13 |
| 240S8 | 13.1 | TST | Chamalea_gallina | 335 | 18.82 | 16.37 | 2.93 | 221.03 | 1 | 3 |
| 240S8 | 13.1 | TST | Chamalea_gallina | 336 | 17.83 | 16.48 | 2.09 | 211.93 | 1 | 5 |
| 240S8 | 13.1 | TST | Chamalea_gallina | 337 | 20 | 16.88 | 2.61 | 241.08 | 1 | 5 |
| 240S8 | 13.1 | TST | Chamalea_gallina | 338 | 18.85 | 16.54 | 2.54 | 226.75 | 1 | 11 |
| 240S8 | 13.1 | TST | Chamalea_gallina | 339 | 19.55 | 17.09 | 2.81 | 238.44 | 1 | 4 |
| 240S8 | 13.1 | TST | Chamalea_gallina | 340 | 18.93 | 16.19 | 3.09 | 221.59 | 1 | 13 |
| 240S8 | 13.1 | TST | Chamalea_gallina | 341 | 20.58 | 17.77 | 2.13 | 264.62 | 0 | 0 |
| 240S8 | 13.1 | TST | Chamalea_gallina | 342 | 19.48 | 17.25 | 2.65 | 243.46 | 1 | 41 |
| 240S8 | 13.1 | TST | Chamalea_gallina | 343 | 18.99 | 16.3 | 2.76 | 221.67 | 0 | 0 |
| 240S8 | 13.1 | TST | Chamalea_gallina | 344 | 20.59 | 18.48 | 2.54 | 273.38 | 1 | 7 |
| 240S8 | 13.1 | TST | Chamalea_gallina | 345 | 18.87 | 16.86 | 2.84 | 230.14 | 0 | 0 |
| 240S8 | 13.1 | TST | Chamalea_gallina | 346 | 21.2 | 18.04 | 2.89 | 277.72 | 1 | 5 |
| 240S8 | 13.1 | TST | Chamalea_gallina | 347 | 20.6 | 18.55 | 2.86 | 274.66 | 0 | 0 |
| 240S8 | 13.1 | TST | Chamalea_gallina | 348 | 21.71 | 18.77 | 3.11 | 292.22 | 1 | 8 |
| 240S8 | 13.1 | TST | Chamalea_gallina | 349 | 20.91 | 17.41 | 2.71 | 261.29 | 1 | 30 |
| 240S8 | 13.1 | TST | Chamalea_gallina | 350 | 18.76 | 16.06 | 2.5 | 218.88 | 1 | 4 |
| 240S8 | 13.1 | TST | Chamalea_gallina | 351 | 20.48 | 17.75 | 2.33 | 260.05 | 0 | 0 |
| 240S8 | 13.1 | TST | Chamalea_gallina | 352 | 18.51 | 16.54 | 2.72 | 220.44 | 1 | 11 |
| 240S8 | 13.1 | TST | Chamalea_gallina | 353 | 20.94 | 18.22 | 2.63 | 275.48 | 0 | 0 |
| 240S8 | 13.1 | TST | Chamalea_gallina | 354 | 19.85 | 17.42 | 3.12 | 251.25 | 1 | 7 |
| 240S8 | 13.1 | TST | Chamalea_gallina | 355 | 19.33 | 16.71 | 2.26 | 232.79 | 1 | 5 |
| 240S8 | 13.1 | TST | Chamalea_gallina | 356 | 18.39 | 16.32 | 3.09 | 217.01 | 1 | 3 |
| 240S8 | 13.1 | TST | Chamalea_gallina | 357 | 18.37 | 16.57 | 2.49 | 220.67 | 1 | 2 |
| 240S8 | 13.1 | TST | Chamalea_gallina | 358 | 19.72 | 16.96 | 2.85 | 242.37 | 1 | 1 |
| 240S8 | 13.7 | TST | Chamalea_gallina | 1 | 2.06 | 2 | 0.21 | 2.83 | 0 | 0 |
| 240S8 | 13.7 | TST | Chamalea_gallina | 2 | 2.54 | 2.6 | 0.14 | 4.79 | 0 | 0 |
| 240S8 | 13.7 | TST | Chamalea_gallina | 3 | 3.15 | 2.98 | 0.42 | 6.87 | 0 | 0 |
| 240S8 | 13.7 | TST | Chamalea_gallina | 4 | 3.18 | 2.85 | 0.35 | 6.56 | 0 | 0 |
| 240S8 | 13.7 | TST | Chamalea_gallina | 5 | 3.36 | 2.86 | 0.5 | 7 | 0 | 0 |
| 240S8 | 13.7 | TST | Chamalea_gallina | 6 | 2.5 | 2.33 | 0.25 | 4.22 | 0 | 0 |
| 240S8 | 13.7 | TST | Chamalea_gallina | 7 | 3.71 | 3.56 | 0.35 | 9.45 | 0 | 0 |
| 240S8 | 13.7 | TST | Chamalea_gallina | 8 | 3.28 | 3.84 | 0.53 | 6.66 | 0 | 0 |
| 240S8 | 13.7 | TST | Chamalea_gallina | 9 | 4.04 | 3.78 | 0.57 | 11 | 0 | 0 |
| 240S8 | 13.7 | TST | Chamalea_gallina | 10 | 4.19 | 3.76 | 0.51 | 11.6 | 0 | 0 |
| 240S8 | 13.7 | TST | Chamalea_gallina | 11 | 4.13 | 3.66 | 0.54 | 11.02 | 0 | 0 |
| 240S8 | 13.7 | TST | Chamalea_gallina | 12 | 3.58 | 3.25 | 0.37 | 8.52 | 0 | 0 |
| 240S8 | 13.7 | TST | Chamalea_gallina | 13 | 4.64 | 4.22 | 0.48 | 14.28 | 0 | 0 |
| 240S8 | 13.7 | TST | Chamalea_gallina | 14 | 4.23 | 3.84 | 0.58 | 12.03 | 0 | 0 |
| 240S8 | 13.7 | TST | Chamalea_gallina | 15 | 4.7 | 4.26 | 0.57 | 14.52 | 0 | 0 |
| 240S8 | 13.7 | TST | Chamalea_gallina | 16 | 4.62 | 4.11 | 0.51 | 13.79 | 0 | 0 |
| 240S8 | 13.7 | TST | Chamalea_gallina | 17 | 5.12 | 4.46 | 0.79 | 16.38 | 0 | 0 |
| 240S8 | 13.7 | TST | Chamalea_gallina | 18 | 5.21 | 4.6 | 0.61 | 17.17 | 0 | 0 |
| 240S8 | 13.7 | TST | Chamalea_gallina | 19 | 5.62 | 4.94 | 0.8 | 19.69 | 0 | 0 |
| 240S8 | 13.7 | TST | Chamalea_gallina | 20 | 5.37 | 4.8 | 0.65 | 18.52 | 0 | 0 |
| 240S8 | 13.7 | TST | Chamalea_gallina | 21 | 5.98 | 5.24 | 0.68 | 23.16 | 0 | 0 |
| 240S8 | 13.7 | TST | Chamalea_gallina | 22 | 6.05 | 5.31 | 0.72 | 23.02 | 0 | 0 |
| 240S8 | 13.7 | TST | Chamalea_gallina | 23 | 5.63 | 5.07 | 0.61 | 20.51 | 0 | 0 |
| 240S8 | 13.7 | TST | Chamalea_gallina | 24 | 4.85 | 4.2 | 0.52 | 14.82 | 0 | 0 |
| 240S8 | 13.7 | TST | Chamalea_gallina | 25 | 5.66 | 4.93 | 0.55 | 19.97 | 1 | 1 |
| 240S8 | 13.7 | TST | Chamalea_gallina | 26 | 6.77 | 5.94 | 0.76 | 28.85 | 0 | 0 |
| 240S8 | 13.7 | TST | Chamalea_gallina | 27 | 7.06 | 6.4 | 0.83 | 32.61 | 0 | 0 |
| 240S8 | 13.7 | TST | Chamalea_gallina | 28 | 6.74 | 5.9 | 0.83 | 28.82 | 1 | 1 |
| 240S8 | 13.7 | TST | Chamalea_gallina | 30 | 8.57 | 7.58 | 0.93 | 46.68 | 0 | 0 |
| 240S8 | 13.7 | TST | Chamalea_gallina | 31 | 8.35 | 7.08 | 0.82 | 42.81 | 0 | 0 |
| 240S8 | 13.7 | TST | Chamalea_gallina | 32 | 8.53 | 7.15 | 0.93 | 43.8 | 0 | 0 |
| 240S8 | 13.7 | TST | Chamalea_gallina | 33 | 9.17 | 8.09 | 1.09 | 53.29 | 0 | 0 |
| 240S8 | 13.7 | TST | Chamalea_gallina | 34 | 8.56 | 7.6 | 0.93 | 47.23 | 1 | 1 |
| 240S8 | 13.7 | TST | Chamalea_gallina | 35 | 10.36 | 8.76 | 1.35 | 64.86 | 1 | 9 |
| 240S8 | 13.7 | TST | Chamalea_gallina | 36 | 9.05 | 8.07 | 1.02 | 53.73 | 0 | 0 |
| 240S8 | 13.7 | TST | Chamalea_gallina | 37 | 10.94 | 9.25 | 1.35 | 74.43 | 1 | 2 |
| 240S8 | 13.7 | TST | Chamalea_gallina | 38 | 10.12 | 8.62 | 1.32 | 61.6 | 1 | 4 |
| 240S8 | 13.7 | TST | Chamalea_gallina | 39 | 9.74 | 8.77 | 1.17 | 61.98 | 1 | 2 |
| 240S8 | 13.7 | TST | Chamalea_gallina | 40 | 10.62 | 9.07 | 1.25 | 68.61 | 0 | 0 |
| 240S8 | 13.7 | TST | Chamalea_gallina | 41 | 10.37 | 8.87 | 1.15 | 67.19 | 0 | 0 |
| 240S8 | 13.7 | TST | Chamalea_gallina | 42 | 10.44 | 8.7 | 1.11 | 65.88 | 0 | 0 |
| 240S8 | 13.7 | TST | Chamalea_gallina | 43 | 11.96 | 10.1 | 1.09 | 86.39 | 0 | 0 |
| 240S8 | 13.7 | TST | Chamalea_gallina | 44 | 10.03 | 8.67 | 1.45 | 62.28 | 0 | 0 |
| 240S8 | 13.7 | TST | Chamalea_gallina | 45 | 9.85 | 8.76 | 1.09 | 63.58 | 1 | 2 |
| 240S8 | 13.7 | TST | Chamalea_gallina | 46 | 12.14 | 10.06 | 1.38 | 89.17 | 0 | 0 |
| 240S8 | 13.7 | TST | Chamalea_gallina | 47 | 11.98 | 10.27 | 1.48 | 89.98 | 1 | 2 |
| 240S8 | 13.7 | TST | Chamalea_gallina | 48 | 10.63 | 9.38 | 1.39 | 71.83 | 1 | 2 |
| 240S8 | 13.7 | TST | Chamalea_gallina | 49 | 11.97 | 10.15 | 1.37 | 87.49 | 0 | 0 |
| 240S8 | 13.7 | TST | Chamalea_gallina | 50 | 13.28 | 11.44 | 1.65 | 110.57 | 0 | 0 |
| 240S8 | 13.7 | TST | Chamalea_gallina | 51 | 16.84 | 14.93 | 2.25 | 180.93 | 1 | 6 |
| 240S8 | 13.7 | TST | Chamalea_gallina | 52 | 17.35 | 15.08 | 2.34 | 188.17 | 1 | 5 |
| 240S8 | 13.7 | TST | Chamalea_gallina | 53 | 18.68 | 15.96 | 2.04 | 209.97 | 1 | 43 |
| 240S8 | 13.7 | TST | Chamalea_gallina | 54 | 16.58 | 14.9 | 2.16 | 176.7 | 1 | 3 |
| 240S8 | 13.7 | TST | Chamalea_gallina | 55 | 16.75 | 14.58 | 1.84 | 179.11 | 1 | 3 |
| 240S8 | 13.7 | TST | Chamalea_gallina | 56 | 20.08 | 17.95 | 1.74 | 250.98 | 1 | 1 |
| 240S8 | 13.7 | TST | Chamalea_gallina | 57 | 17.56 | 14.85 | 1.95 | 188.71 | 1 | 2 |
| 240S8 | 13.7 | TST | Chamalea_gallina | 58 | 20 | 17.83 | 1.9 | 251.37 | 1 | 1 |
| 240S8 | 13.7 | TST | Chamalea_gallina | 59 | 10.15 | 8.8 | 1.04 | 64.53 | 1 | 2 |
| 240S8 | 13.7 | TST | Chamalea_gallina | 60 | 26.37 | 21.65 | 3.1 | 409.95 | 0 | 0 |
