# Supplementary material for: Parasitism and host behavior in the context of a changing environment: The Holocene record of the commercially important bivalve Chamelea gallina, northern Italy
Source: PLoS One. 2021 Apr 1;16(4):e0247790. doi: 10.1371/journal.pone.0247790 (PMC8016236; doi:10.1371/journal.pone.0247790)
Supplement: S2 Table — (DOCX) [file pone.0247790.s002.docx]

**S2 Table. Summary data by sample (indicated by WellDepth).** nWhole: number of whole *C. gallina* valves in a sample. nPits: number of whole *C. gallina* valves with at least one trematode pit in a sample. SumNoTrematodes: number of trematode-induced pits in a sample. Prevalence: nPits/nWhole. CI2.5 and CI97.5: upper and lower bounds of 95% confidence intervals of Prevalence. Mean.X.Trematodes: mean number of trematode pits per whole valve in a sample. Var.X.Trematodes: variance of trematode pits per whole valve in a sample. These data are referred to in “ChamaleaSummary” in *R* code appended below.

| Core | WellDepth | nWhole | nPits | SumNoTrematodes | Prevalence | CI2.5 | CI97.5 | Mean.X.Trematodes | Var.X.Trematodes |
| --- | --- | --- | --- | --- | --- | --- | --- | --- | --- |
| 223S5 | 14.9 | 20 | 1 | 4 | 0.050 | 0.003 | 0.269 | 0.200 | 0.800 |
| 240S8 | 13.5 | 87 | 35 | 208 | 0.402 | 0.300 | 0.513 | 2.391 | 32.194 |
| 240S8 | 14.8 | 249 | 2 | 4 | 0.008 | 0.001 | 0.032 | 0.016 | 0.040 |
| 240S8 | 12.5 | 29 | 4 | 21 | 0.138 | 0.045 | 0.326 | 0.724 | 4.993 |
| 240S8 | 13.1 | 358 | 111 | 563 | 0.310 | 0.263 | 0.361 | 1.573 | 18.654 |
| 240S8 | 13.7 | 59 | 19 | 92 | 0.322 | 0.21 | 0.458 | 1.559 | 33.001 |
