# Supplementary material for: Parasitism and host behavior in the context of a changing environment: The Holocene record of the commercially important bivalve Chamelea gallina, northern Italy
Source: PLoS One. 2021 Apr 1;16(4):e0247790. doi: 10.1371/journal.pone.0247790 (PMC8016236; doi:10.1371/journal.pone.0247790)
Supplement: S1 Appendix — (DOCX) [file pone.0247790.s003.docx]

**S1 Appendix. *R* script used for data analysis and figure construction.** “Chamalea” refers to data in Supporting Information Table 1. “ChamaleaSummary” refers to data in Supporting Information Table 2.

Chamalea <- read.csv("ChamaleaCores.csv", header = TRUE, sep = ",")

attach(Chamalea)

Chamalea$Length <- as.character(Chamalea$Length)

Chamalea$Width <- as.character(Chamalea$Height)

Chamalea$Sinus <- as.character(Chamalea$Sinus)

Chamalea$Area <- as.character(Chamalea$Area)

Chamalea$X.Trematodes <- as.character(Chamalea$X.Trematodes)

Chamalea$Well.Depth..m. <- as.factor(Chamalea$Well.Depth..m.)

Chamalea$Length <- as.numeric(Chamalea$Length)

Chamalea$Width <- as.numeric(Chamalea$Height)

Chamalea$Sinus <- as.numeric(Chamalea$Sinus)

Chamalea$Area <- as.numeric(Chamalea$Area)

Chamalea$X.Trematodes <- as.numeric(Chamalea$X.Trematodes)

attach(Chamalea)

library(ggplot2)

library(factoextra)

library(plyr)

library(dplyr)

library(ggthemes)

library(viridis)

library(boot)

library(lmodel2)

#Conduct a PCA of all specimens based on Length, Width, Area, and Sinus

ChamaleaPCA <- prcomp(log10(Chamalea[,c(6:9)]), scale = TRUE)

#Merge PC scores with WholeMulinia

TotalChamalea <- merge(Chamalea, ChamaleaPCA$x, by="row.names", all=TRUE)

attach(TotalChamalea)

#Create a scree plot for PCA

fviz_eig(ChamaleaPCA, pointsize=2, geom = "line") + theme_classic() + theme(text = element_text(size=15))

#Create loadings plots for PC1vPC2 etc.

#Update the axis values for each comparison!

fviz_pca_var(ChamaleaPCA, axes = c(1,2), geom = c("text","point"), labelsize=6, repel = TRUE) + theme_classic() + theme(text = element_text(size=15)) + ggtitle("Loadings Plot")

#Create a better loadings plot for PC1vPC2 by calculating Pearson correlation coefficients between PC1, PC2 and Length, Width, Area, and Sinus

#Create an object with these correlation coefficients

#Produce scatterplot from this new object

attach(TotalChamalea)

PC1L <- cor.test(PC1, Length)

PC1W <- cor.test(PC1, Width)

PC1A <- cor.test(PC1, Area)

PC1S <- cor.test(PC1, Sinus)

PC2L <- cor.test(PC2, Length)

PC2W <- cor.test(PC2, Width)

PC2A <- cor.test(PC2, Area)

PC2S <- cor.test(PC2, Sinus)

LoadingsCoefficients <- matrix(c(PC1L$estimate, PC1W$estimate, PC1A$estimate, PC1S$estimate, PC2L$estimate, PC2W$estimate, PC2A$estimate, PC2S$estimate), ncol = 2)

colnames(LoadingsCoefficients) <- c("PC1", "PC2")

rownames(LoadingsCoefficients) <- c("Length", "Width", "Area", "Sinus")

LoadingsCoefficients <- as.data.frame(LoadingsCoefficients)

attach(LoadingsCoefficients)

ggplot(data = LoadingsCoefficients) + geom_point(aes(x=PC1, y=PC2), size=0.5) + theme_classic() + xlim(c(-1,1)) + ylim(c(-1,1)) + geom_vline(xintercept = 0, linetype=2) + geom_hline(yintercept = 0, linetype=2) + theme(text = element_text(size=20))

#Plot PC1 v PC2 exploring relationships between size, morphology, trematode prevalence/intensity, strata, and systems tracts

attach(TotalChamalea)

PCAPlot <- ggplot(data = TotalChamalea) + geom_point(aes(PC1,PC2), size=3) + theme_classic() + xlab("PC1 (98.21%)") + ylab("PC2 (1.74%)") + theme(axis.text = element_text(size=20)) + theme(axis.title = element_text(size = 20))

ggplot(data = TotalChamalea) + geom_point(aes(PC1,PC2, color=Infestation, size=X.Trematodes)) + theme_classic() + xlab("PC1 (98.21%)") + ylab("PC2 (1.74%)") + scale_color_colorblind() + theme(axis.text = element_text(size=20)) + theme(axis.title = element_text(size = 20))

PCATrematode <- ggplot() + geom_rug(data=TotalChamalea, aes(x=PC1, y=PC2, color=as.factor(Infestation)), size=0.5) + geom_density2d(data=TotalChamalea, aes(PC1,PC2, group=Infestation)) + stat_density2d(data=TotalChamalea, aes(PC1, PC2, group=Infestation, color=as.factor(Infestation), fill=..level..), geom="polygon", alpha=0.1) + scale_fill_gradient(low = "dark grey", high = "black") + scale_color_colorblind() + geom_point(data = subset(TotalChamalea, Infestation=="1"), aes(median(PC1), median(PC2)), size=5, shape=2) + geom_point(data = subset(TotalChamalea, Infestation=="0"), aes(median(PC1), median(PC2)), size=5, shape=17) + scale_x_continuous() + scale_y_continuous() + xlab("PC1 (98.21%)") + ylab("PC2 (1.74%)") + theme_classic() + theme(text = element_text(size=20)) + theme(legend.position = "none")

wilcox.test(subset(PC1, Infestation=="1"), subset(PC1, Infestation=="0"))

wilcox.test(subset(PC2, Infestation=="1"), subset(PC2, Infestation=="0"))

attach(TotalChamalea)

#How does body size vary across strata by infestation?

TotalChamalea12.5 <- subset(TotalChamalea, Well.Depth..m.=="12.5")

TotalChamalea13.1 <- subset(TotalChamalea, Well.Depth..m.=="13.1")

TotalChamalea13.5 <- subset(TotalChamalea, Well.Depth..m.=="13.5")

TotalChamalea14.8 <- subset(TotalChamalea, Well.Depth..m.=="14.8")

TotalChamalea14.9 <- subset(TotalChamalea, Well.Depth..m.=="14.9")

TotalChamalea13.7 <- subset(TotalChamalea, Well.Depth..m.=="13.7")

attach(TotalChamalea12.5)

median(subset(TotalChamalea12.5$PC1, Infestation=="1"))

median(subset(TotalChamalea12.5$PC1, Infestation=="0"))

wilcox.test(subset(TotalChamalea12.5$PC1, Infestation=="0"), subset(TotalChamalea12.5$PC1, Infestation=="1"))

attach(TotalChamalea13.1)

median(subset(TotalChamalea13.1$PC1, Infestation=="1"))

median(subset(TotalChamalea13.1$PC1, Infestation=="0"))

wilcox.test(subset(TotalChamalea13.1$PC1, Infestation=="0"), subset(TotalChamalea13.1$PC1, Infestation=="1"))

attach(TotalChamalea13.5)

median(subset(TotalChamalea13.5$PC1, Infestation=="1"))

median(subset(TotalChamalea13.5$PC1, Infestation=="0"))

wilcox.test(subset(TotalChamalea13.5$PC1, Infestation=="0"), subset(TotalChamalea13.5$PC1, Infestation=="1"))

attach(TotalChamalea13.7)

median(subset(TotalChamalea13.7$PC1, Infestation=="1"))

median(subset(TotalChamalea13.7$PC1, Infestation=="0"))

wilcox.test(subset(TotalChamalea13.7$PC1, Infestation=="0"), subset(TotalChamalea13.7$PC1, Infestation=="1"))

attach(TotalChamalea14.8)

median(subset(TotalChamalea14.8$PC1, Infestation=="1"))

median(subset(TotalChamalea14.8$PC1, Infestation=="0"))

wilcox.test(subset(TotalChamalea14.8$PC1, Infestation=="0"), subset(TotalChamalea14.8$PC1, Infestation=="1"))

attach(TotalChamalea14.9)

median(subset(TotalChamalea14.9$PC1, Infestation=="1"))

median(subset(TotalChamalea14.9$PC1, Infestation=="0"))

wilcox.test(subset(TotalChamalea14.9$PC1, Infestation=="0"), subset(TotalChamalea14.9$PC1, Infestation=="1"))

#How does relative Sinus size vary across strata by infestation?

TotalChamalea$Infestation <- as.character(TotalChamalea$Infestation)

ggplot(data = subset(TotalChamalea, Well.Depth..m.=="12.5")) + geom_histogram(aes(x=PC2, fill=Infestation)) + theme_classic() + ylab("Frequency") + theme_classic() + xlim(c(-2,1)) + ylim(c(0,80)) + scale_fill_colorblind() + ggtitle(("12.5 m")) + theme(axis.text = element_text(size=20)) + theme(axis.title = element_text(size = 20))

ggplot(data = subset(TotalChamalea, Well.Depth..m.=="13.1")) + geom_histogram(aes(x=PC2, fill=Infestation)) + theme_classic() + ylab("Frequency") + theme_classic() + xlim(c(-2,1)) + ylim(c(0,80)) + scale_fill_colorblind() + ggtitle(("13.1 m")) + theme(axis.text = element_text(size=20)) + theme(axis.title = element_text(size = 20))

ggplot(data = subset(TotalChamalea, Well.Depth..m.=="13.5")) + geom_histogram(aes(x=PC2, fill=Infestation)) + theme_classic() + ylab("Frequency") + theme_classic() + xlim(c(-2,1)) + ylim(c(0,80)) + scale_fill_colorblind() + ggtitle(("13.5 m")) + theme(axis.text = element_text(size=20)) + theme(axis.title = element_text(size = 20))

ggplot(data = subset(TotalChamalea, Well.Depth..m.=="13.7")) + geom_histogram(aes(x=PC2, fill=Infestation)) + theme_classic() + ylab("Frequency") + theme_classic() + xlim(c(-2,1)) + ylim(c(0,80)) + scale_fill_colorblind() + ggtitle(("13.7 m")) + theme(axis.text = element_text(size=20)) + theme(axis.title = element_text(size = 20))

ggplot(data = subset(TotalChamalea, Well.Depth..m.=="14.8")) + geom_histogram(aes(x=PC2, fill=Infestation)) + theme_classic() + ylab("Frequency") + theme_classic() + xlim(c(-2,1)) + ylim(c(0,80)) + scale_fill_colorblind() + ggtitle(("14.8 m")) + theme(axis.text = element_text(size=20)) + theme(axis.title = element_text(size = 20))

ggplot(data = subset(TotalChamalea, Well.Depth..m.=="14.9")) + geom_histogram(aes(x=PC2, fill=Infestation)) + theme_classic() + ylab("Frequency") + theme_classic() + xlim(c(-2,1)) + ylim(c(0,80)) + scale_fill_colorblind() + ggtitle(("14.9 m")) + theme(axis.text = element_text(size=20)) + theme(axis.title = element_text(size = 20))

attach(TotalChamalea12.5)

median(subset(TotalChamalea12.5$PC2, Infestation=="1"))

median(subset(TotalChamalea12.5$PC2, Infestation=="0"))

wilcox.test(subset(TotalChamalea12.5$PC2, Infestation=="0"), subset(TotalChamalea12.5$PC2, Infestation=="1"))

attach(TotalChamalea13.1)

median(subset(TotalChamalea13.1$PC2, Infestation=="1"))

median(subset(TotalChamalea13.1$PC2, Infestation=="0"))

wilcox.test(subset(TotalChamalea13.1$PC2, Infestation=="0"), subset(TotalChamalea13.1$PC2, Infestation=="1"))

attach(TotalChamalea13.5)

median(subset(TotalChamalea13.5$PC2, Infestation=="1"))

median(subset(TotalChamalea13.5$PC2, Infestation=="0"))

wilcox.test(subset(TotalChamalea13.5$PC2, Infestation=="0"), subset(TotalChamalea13.5$PC2, Infestation=="1"))

attach(TotalChamalea13.7)

median(subset(TotalChamalea13.7$PC2, Infestation=="1"))

median(subset(TotalChamalea13.7$PC2, Infestation=="0"))

wilcox.test(subset(TotalChamalea13.7$PC2, Infestation=="0"), subset(TotalChamalea13.7$PC2, Infestation=="1"))

attach(TotalChamalea14.8)

median(subset(TotalChamalea14.8$PC2, Infestation=="1"))

median(subset(TotalChamalea14.8$PC2, Infestation=="0"))

wilcox.test(subset(TotalChamalea14.8$PC2, Infestation=="0"), subset(TotalChamalea14.8$PC2, Infestation=="1"))

attach(TotalChamalea14.9)

median(subset(TotalChamalea14.9$PC2, Infestation=="1"))

median(subset(TotalChamalea14.9$PC2, Infestation=="0"))

wilcox.test(subset(TotalChamalea14.9$PC2, Infestation=="0"), subset(TotalChamalea14.9$PC2, Infestation=="1"))

#How does trematode prevalence vary across strata?

ChamaleaSummary <- read.csv("ChamaleaSummary.csv", header = TRUE, sep = ",")

attach(ChamaleaSummary)

#Calculate 95% CI for prevalence values in each stratum

prop.test(subset(ChamaleaSummary$nPits, SampleNo=="57"), subset(ChamaleaSummary$nWhole, SampleNo=="57"))

prop.test(subset(ChamaleaSummary$nPits, SampleNo=="58"), subset(ChamaleaSummary$nWhole, SampleNo=="58"))

prop.test(subset(ChamaleaSummary$nPits, SampleNo=="59"), subset(ChamaleaSummary$nWhole, SampleNo=="59"))

prop.test(subset(ChamaleaSummary$nPits, SampleNo=="60"), subset(ChamaleaSummary$nWhole, SampleNo=="60"))

prop.test(subset(ChamaleaSummary$nPits, SampleNo=="78"), subset(ChamaleaSummary$nWhole, SampleNo=="78"))

#95% CI for prevalence value of new sample at 13.7 m

prop.test(19,59)

#Update ChamaleaSummary csv sheet with these values and reupload the .csv file

attach(ChamaleaSummary)

ggplot(data = subset(ChamaleaSummary, Core=="240S8")) + geom_point(aes(Prevalence,WellDepth), size=4) + geom_point(aes(CI2.5,WellDepth), shape=3) + geom_point(aes(CI97.5,WellDepth), shape=3) + theme_classic() + xlim(c(0,1)) + scale_y_reverse() + theme(axis.text = element_text(size=20)) + theme(axis.title = element_text(size = 20))

ggplot(data = subset(ChamaleaSummary, Core=="223S5")) + geom_point(aes(Prevalence,WellDepth), size=4) + geom_point(aes(CI2.5,WellDepth), shape=3) + geom_point(aes(CI97.5,WellDepth), shape=3) + xlim(c(0,1)) + theme_classic() + scale_y_reverse() + theme(axis.text = element_text(size=20)) + theme(axis.title = element_text(size = 20))

#How does mean intensity vary across strata?

attach(ChamaleaSummary)

ggplot(data = subset(ChamaleaSummary, Core=="240S8")) + geom_point(aes(Mean.X.Trematodes,WellDepth), size=4) + theme_classic() + xlim(c(0,3)) + scale_y_reverse() + theme(axis.text = element_text(size=20)) + theme(axis.title = element_text(size = 20))

ggplot(data = subset(ChamaleaSummary, Core=="223S5")) + geom_point(aes(Mean.X.Trematodes,WellDepth), size=4) + theme_classic() + xlim(c(0,3)) + scale_y_reverse() + theme(axis.text = element_text(size=20)) + theme(axis.title = element_text(size = 20))

#How does variance:mean ratio of trematode pits across strata?

attach(ChamaleaSummary)

ggplot(data = subset(ChamaleaSummary, LocNo=="3")) + geom_point(aes(VarMean,WellDepth), size=4) + theme_classic() + xlim(c(0,25)) + scale_y_reverse() + theme(axis.text = element_text(size=20)) + theme(axis.title = element_text(size = 20))

ggplot(data = subset(ChamaleaSummary, LocNo=="2")) + geom_point(aes(VarMean,WellDepth), size=4) + theme_classic() + xlim(c(0,25)) + scale_y_reverse() + theme(axis.text = element_text(size=20)) + theme(axis.title = element_text(size = 20))

#Are trematodes evenly distributed among host individuals?

attach(TotalChamalea)

ggplot(data = TotalChamalea) + geom_bar(aes(X.Trematodes), width = 1) + theme_classic() + xlim(c(-2,50)) + xlab("Number of Trematode Pits") + ylab("Number of Host Valves") + theme(axis.text = element_text(size=20)) + theme(axis.title = element_text(size = 20))

ggplot(data = subset(TotalChamalea, Infestation=="1")) + geom_bar(aes(X.Trematodes, fill=Well.Depth..m.), width = 1) + scale_fill_colorblind() + theme_classic() + labs(fill = "Well Depth (m)") + theme(legend.text=element_text(size=16)) + theme(legend.title = element_text(size = 16)) + theme(legend.position = c(0.8, 0.3))+ xlim(c(-2,50)) + xlab("Number of Trematode Pits") + ylab("Number of Host Valves") + theme(axis.text = element_text(size=20)) + theme(axis.title = element_text(size = 20))

#How does trematode intensity vary by body size and shape?

attach(TotalChamalea)

ggplot(data=subset(TotalChamalea, Infestation=="1")) + geom_point(aes(PC1,X.Trematodes), size=3, alpha=0.7) + geom_smooth(aes(PC1,X.Trematodes)) + theme_classic() + scale_x_reverse() + scale_y_log10() + ylab("Number of Trematode Traces") + theme(axis.text = element_text(size=20)) + theme(axis.title = element_text(size = 20))

cor.test(subset(PC1, Infestation=="1"), subset(X.Trematodes, Infestation=="1"))

ggplot(data=subset(TotalChamalea, Infestation=="1")) + geom_point(aes(PC2,X.Trematodes), size=3, alpha=0.7) + geom_smooth(aes(PC2,X.Trematodes)) + theme_classic() + scale_y_log10() + ylab("Number of Trematode Traces") + theme(axis.text = element_text(size=20)) + theme(axis.title = element_text(size = 20))

cor.test(subset(PC2, Infestation=="1"), subset(X.Trematodes, Infestation=="1"))

#Test for aggregation of parasites by sample by comparing mean intensity and variance of intensity

#Calculate these values for each sample, enter them into ChamaleaSummary, and re-import .csv

mean(TotalChamalea13.1$X.Trematodes)

var(TotalChamalea13.1$X.Trematodes)

ChamaleaSummary <- read.csv("ChamaleaSummary.csv", header = TRUE, sep = ",")

attach(ChamaleaSummary)

#Define endpoints of line segment to serve as 1:1 of var:mean for aggregation plot

linepoints <- data.frame(x1 = 0.01, x2 = 10.0, y1 = 0.01, y2 = 10.0)

#Plot mean intensity and variance of intensity

ggplot(data = ChamaleaSummary) + geom_smooth(data = ChamaleaSummary, aes(x=Mean.X.Trematodes, y=Var.X.Trematodes), method = lm, se=FALSE, color="black", size=1) +

geom_point(aes(x=Mean.X.Trematodes, y=Var.X.Trematodes, size=nWhole, fill=Prevalence), shape=21) +

geom_segment(data=linepoints, aes(x=x1, y=y1, xend=x2, yend=y2), linetype="dashed", size=2) +

scale_x_log10() + scale_y_log10() + xlab("Mean Number of Pits") + ylab("Variance Number of Pits") + theme_classic() +

theme(axis.text = element_text(size=20)) + theme(axis.title = element_text(size = 20)) + theme(legend.position = c(0.8, 0.25)) + scale_fill_viridis(option = "plasma")

AggregationRegression <- lmodel2(log10(Var.X.Trematodes) ~ log10(Mean.X.Trematodes), TotalChamalea)

#Compile slope and 95%CI values for OLS and plot as inset for aggregation plot to show how the slope differs from the 1:1 line

AggregationSlope <- data.frame(slope = 1.388971, CI2.5 = 1.151920, CI97.5 = 1.626023)

attach(AggregationSlope)

ggplot(AggregationSlope) + geom_point(aes(1, slope), size=4) + geom_point(aes(1, CI2.5), shape=3) + geom_point(aes(1, CI97.5), shape=3) + theme_classic() + ylim(c(-2,2))

#Create facet plots for each core with WaterDepth, Prevalence, Mean Intensity, Var:Mean, nWhole, ProportionBivalve

attach(ChamaleaSummary)

#Core 240S8/Well 3

ggplot(data = subset(ChamaleaSummary, Core=="240S8")) + geom_point(aes(WaterDepth,WellDepth), size=4) + theme_classic() + xlim(c(5,15)) + scale_y_reverse() + ylim(c(15,12)) + theme(axis.text = element_text(size=20) ) + theme(axis.title = element_text(size = 20))

ggplot(data = subset(ChamaleaSummary, Core=="240S8")) + geom_point(aes(Prevalence,WellDepth), size=4) + geom_point(aes(CI2.5,WellDepth), shape=3) + geom_point(aes(CI97.5,WellDepth), shape=3) + theme_classic() + xlim(c(0,1)) + scale_y_reverse() + ylim(c(15,12)) + theme(axis.text = element_text(size=20)) + theme(axis.title = element_text(size = 20))

ggplot(data = subset(ChamaleaSummary, Core=="240S8")) + geom_point(aes(Mean.X.Trematodes,WellDepth), size=4) + theme_classic() + xlim(c(0,3)) + scale_y_reverse() + ylim(c(15,12)) + theme(axis.text = element_text(size=20)) + theme(axis.title = element_text(size = 20))

ggplot(data = subset(ChamaleaSummary, Core=="240S8")) + geom_point(aes(VarMean,WellDepth), size=4) + theme_classic() + xlim(c(0,15)) + scale_y_reverse() + ylim(c(15,12)) + theme(axis.text = element_text(size=20)) + theme(axis.title = element_text(size = 20))

ggplot(data = subset(ChamaleaSummary, Core=="240S8")) + geom_point(aes(nWhole,WellDepth), size=4) + theme_classic() + xlim(c(0,400)) + scale_y_reverse() + ylim(c(15,12)) + theme(axis.text = element_text(size=20)) + theme(axis.title = element_text(size = 20))

ggplot(data = subset(ChamaleaSummary, Core=="240S8")) + geom_point(aes(BivalveProportion,WellDepth), size=4) + theme_classic() + xlim(c(0,1)) + scale_y_reverse() + ylim(c(15,12)) + theme(axis.text = element_text(size=20)) + theme(axis.title = element_text(size = 20))

#Core 223S5/Well 2

ggplot(data = subset(ChamaleaSummary, Core=="223S5")) + geom_point(aes(WaterDepth,WellDepth), size=4) + theme_classic() + xlim(c(5,15)) + scale_y_reverse() + ylim(c(15,12)) + theme(axis.text = element_text(size=20) ) + theme(axis.title = element_text(size = 20))

ggplot(data = subset(ChamaleaSummary, Core=="223S5")) + geom_point(aes(Prevalence,WellDepth), size=4) + geom_point(aes(CI2.5,WellDepth), shape=3) + geom_point(aes(CI97.5,WellDepth), shape=3) + theme_classic() + xlim(c(0,1)) + scale_y_reverse() + ylim(c(15,12)) + theme(axis.text = element_text(size=20)) + theme(axis.title = element_text(size = 20))

ggplot(data = subset(ChamaleaSummary, Core=="223S5")) + geom_point(aes(Mean.X.Trematodes,WellDepth), size=4) + theme_classic() + xlim(c(0,3)) + scale_y_reverse() + ylim(c(15,12)) + theme(axis.text = element_text(size=20)) + theme(axis.title = element_text(size = 20))

ggplot(data = subset(ChamaleaSummary, Core=="223S5")) + geom_point(aes(VarMean,WellDepth), size=4) + theme_classic() + xlim(c(0,15)) + scale_y_reverse() + ylim(c(15,12)) + theme(axis.text = element_text(size=20)) + theme(axis.title = element_text(size = 20))

ggplot(data = subset(ChamaleaSummary, Core=="223S5")) + geom_point(aes(nWhole,WellDepth), size=4) + theme_classic() + xlim(c(0,400)) + scale_y_reverse() + ylim(c(15,12)) + theme(axis.text = element_text(size=20)) + theme(axis.title = element_text(size = 20))

ggplot(data = subset(ChamaleaSummary, Core=="223S5")) + geom_point(aes(BivalveProportion,WellDepth), size=4) + theme_classic() + xlim(c(0,1)) + scale_y_reverse() + ylim(c(15,12)) + theme(axis.text = element_text(size=20)) + theme(axis.title = element_text(size = 20))

#Export as eps

ggsave(filename = "PCATrematode.eps",

plot = print(PCATrematode),

device = cairo_ps)
